# Polyyne [3]Rotaxanes: Synthesis via Dicobalt Carbonyl Complexes and Enhanced Stability

**DOI:** 10.1002/anie.202116897

**Published:** 2022-01-20

**Authors:** Connor W. Patrick, Joseph F. Woods, Przemyslaw Gawel, Claire E. Otteson, Amber L. Thompson, Tim D. W. Claridge, Ramesh Jasti, Harry L. Anderson

**Affiliations:** ^1^ Department of Chemistry University of Oxford Chemistry Research Laboratory Oxford OX1 3TA UK; ^2^ Department of Chemistry and Biochemistry Materials Science Institute University of Oregon Eugene OR 97403 USA

**Keywords:** Rotaxanes, Acetylene, Polyynes, Thermal Stability, Template-Directed Synthesis

## Abstract

New strategies for synthesizing polyyne polyrotaxanes are being developed as an approach to stable carbyne “insulated molecular wires”. Here we report an active metal template route to polyyne [3]rotaxanes, using dicobalt carbonyl masked alkyne equivalents. We synthesized two [3]rotaxanes, both with the same C_28_ polyyne dumbbell component, one with a phenanthroline‐based macrocycle and one using a 2,6‐pyridyl cycloparaphenylene nanohoop. The thermal stabilities of the two rotaxanes were compared with that of the naked polyyne dumbbell in decalin at 80 °C, and the nanohoop rotaxane was found to be 4.5 times more stable.

## Graphical abstract

Cobalt carbonyl complexes have been used as temporary masking group, and temporary stoppers, in the synthesis of polyyne [3]rotaxanes with two macrocycles threaded on a C_28_ thread. Comparison of the thermal stability of two [3]rotaxanes with different threaded macrocycles shows that the 2,6‐pyridyl cycloparaphenylene nanohoop effectively stabilizes the polyyne chain.

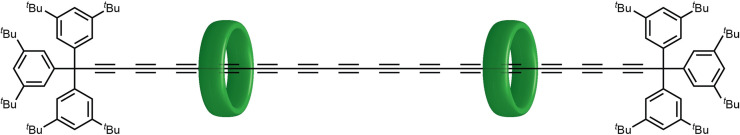

Reactive π‐systems can be stabilized by threading them through protective macrocycles to generate rotaxanes or polyrotaxanes, as “insulated molecular wires”.^[1]^ This concept has been used to enhance the properties of many organic semiconductors and dyes.[^1^, ^2^, ^3^] One of the most interesting π‐systems to select for stabilization in this way is carbyne, the 1D sp‐hybridized allotrope of carbon,^[4]^ because it seems unlikely that carbyne can exist as a pure carbon allotrope without some type of supramolecular encapsulation.^[5]^ Bulky terminal groups stabilize polyynes (i.e. oligomers of carbyne) with up to 24 contiguous alkyne units,^[6]^ but stabilization from the end groups is expected to diminish with increasing chain length, whereas polyrotaxane formation could stabilize polyynes of any length, making it possible to study the properties of long carbyne chains in solution. [2]Rotaxanes consisting of a single macrocycle threaded on a polyyne dumbbell are readily prepared using active metal templates;[^7^, ^8^, ^9^, ^10^] the challenge is to synthesize long polyynes with many threaded macrocycles. One potential solution to this problem is to use bulky masked alkyne equivalents (MAEs) which can subsequently be converted into alkynes, and which act as stoppers on a rotaxane intermediate.^[9]^ Rotaxanes with MAE stoppers are promising precursors to carbyne polyrotaxanes and cyclocarbon catenanes.^[9]^ Previously, we and others have investigated dicobalt carbonyl complexes as MAEs,[^11^, ^12^] but attempts at synthesizing rotaxanes with these stoppers were unsuccessful.^[11]^ Here we report the first synthesis of polyyne rotaxanes with dicobalt carbonyl MAE stoppers and the conversion of these [2]rotaxanes to polyyne [3]rotaxanes with 14 contiguous alkyne units, **1⋅**(**M1**)_2_ and **1⋅**(**M2**)_2_ (Scheme 1). We also report the enhanced thermal stability of the [3]rotaxane **1⋅**(**M2**)_2_, compared with the corresponding C_28_ dumbbell.

**Scheme 1:**
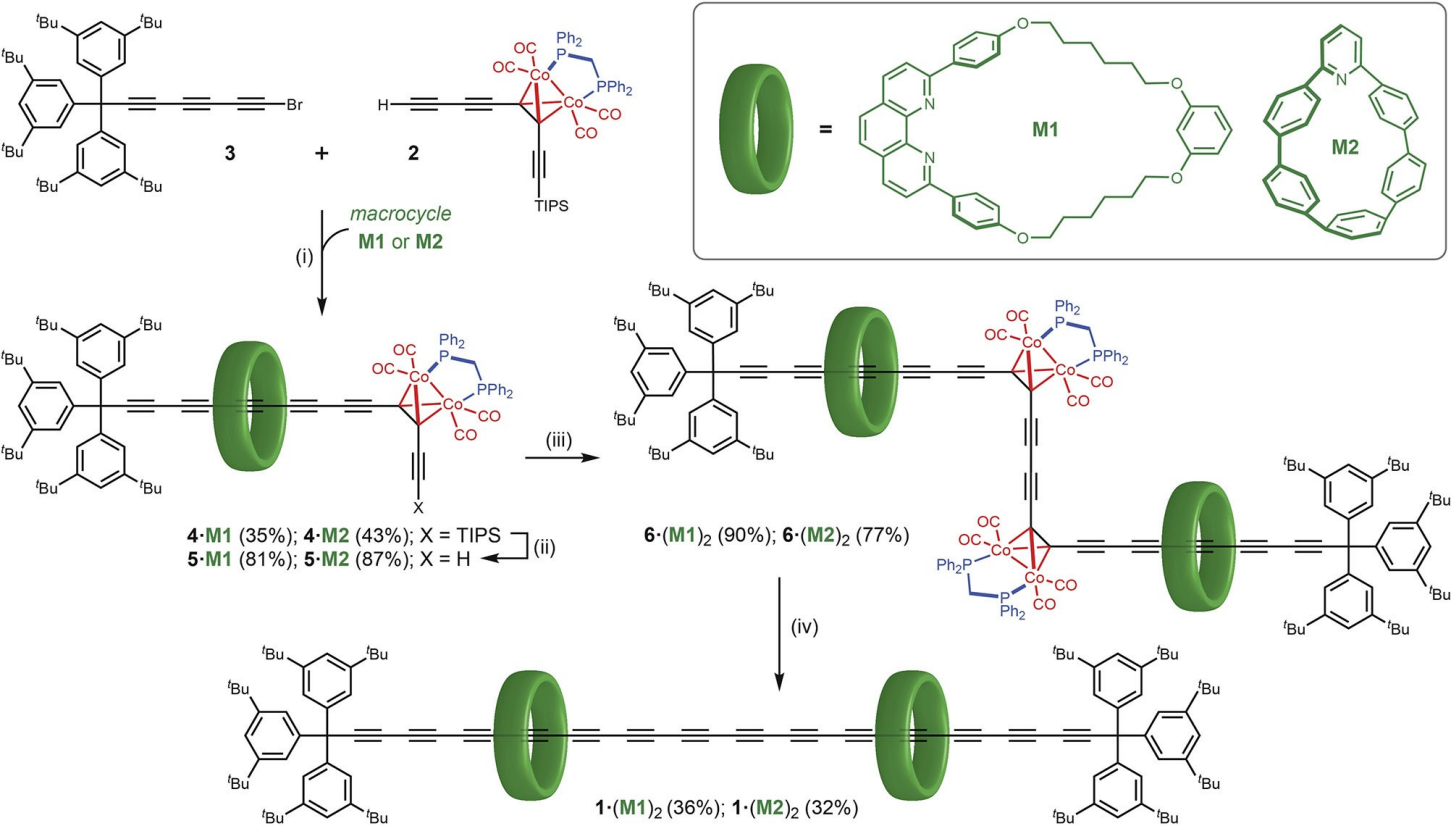
Synthesis of the polyyne [3]rotaxanes **1⋅**(**M1**)_2_ and **1⋅**(**M2**)_2_; i) **M1**⋅CuI, K_2_CO_3_, THF, 15 h, 60 °C; **M2**, [Cu(MeCN)_4_][PF_6_], *i‐*Pr_2_NEt, CHCl_3_, 18 h, 60 °C; ii) TBAF, THF, 30 min, 20 °C; iii) **M1**: CuCl, TMEDA, CH_2_Cl_2_, 30 min, 20 °C, O_2_; **M2**: CuCl, 4,4′‐di‐*t‐*butyl‐2,2′‐bipyridine, CH_2_Cl_2_, 20 h, 30 °C, O_2_; iv) **M1**: I_2_, THF, 3 h, 20 °C, **M2**: I_2_, THF, MeCN (1 : 1 *v*/*v*), 5 min, 20 °C.

Two [3]rotaxanes were targeted in this study: one based on a larger phenanthroline macrocycle **M1**, pioneered by Saito,^[7a]^ and the other using a smaller 2,6‐pyridyl cycloparaphenylene (nanohoop) **M2**, developed by Jasti and co‐workers.^[10]^ Many rotaxanes have been reported based on the Saito macrocycle **M1**, but molecular models indicate that it is too large and flexible to provide effective protection of a threaded polyyne. Crystal structures of rotaxanes based on **M1** also show that the 2,9‐diarylphenanthroline tends to form stacked aggregates,^[13]^ which could reduce the screening of the polyyne thread in these [3]rotaxanes. In contrast, the nanohoop is expected to provide better shielding of the polyyne.

The synthesis of the [3]rotaxanes starts from terminal alkyne **2** (Scheme 1), which is readily available from TMS‐C_6_‐TIPS,^[14]^ as reported previously.^[11]^ Active metal‐template Cadiot‐Chodkiewicz cross coupling of **2** with supertrityl bromo‐triyne **3** in the presence of macrocycles **M1** or **M2** gave the [2]rotaxanes **4⋅M1** and **4⋅M2**, although it was necessary to optimize the reaction conditions for each macrocycle. With the phenanthroline macrocycle, the **M1⋅**CuI complex was pre‐formed and cross coupling was carried out in THF, with K_2_CO_3_ as the base, as previously reported,[^8c^, ^9b^, ^15^] to give [2]rotaxane **4⋅M1** in 35 % isolated yield. In contrast, the nanohoop **M2** did not form the target [2]rotaxane **4⋅M2** under these conditions; instead, only the non‐interlocked dumbbell **4** was produced, presumably because its pyridine unit does not bind strongly enough to copper(I) cations in coordinating solvents such as THF. Changing to a non‐coordinating solvent (CHCl_3_), with diisopropylethylamine as the base[^10^, ^16^] afforded the desired [2]rotaxane **4⋅M2** in 43 % yield. Crystals of **4⋅M1** suitable for single‐crystal X‐ray diffraction^[17]^ were grown by layered addition of methanol to a solution in dichloromethane, followed by slow evaporation of the solvent. Despite considerable efforts, it was only possible to grow poor quality crystals that were highly unstable to solvent loss. The structure has four **4⋅M1** rotaxane moieties in the asymmetric unit and there is significant disorder, contributing to an absence of high‐resolution data. To ensure sensible displacement parameters and that the local geometry remained feasible, restraints were required, so it is not possible to compare derived parameters in detail. In spite of this, it is clear that all four molecules have similar geometries, with the PPh_2_CH_2_PPh_2_ ligand oriented towards the TIPS group, away from the polyyne, so that the macrocycle is buttressed by four carbonyl groups at one face and by the three *t‐*Bu groups of a supertrityl stopper at the other face (Figure 1).

**Figure 1:**
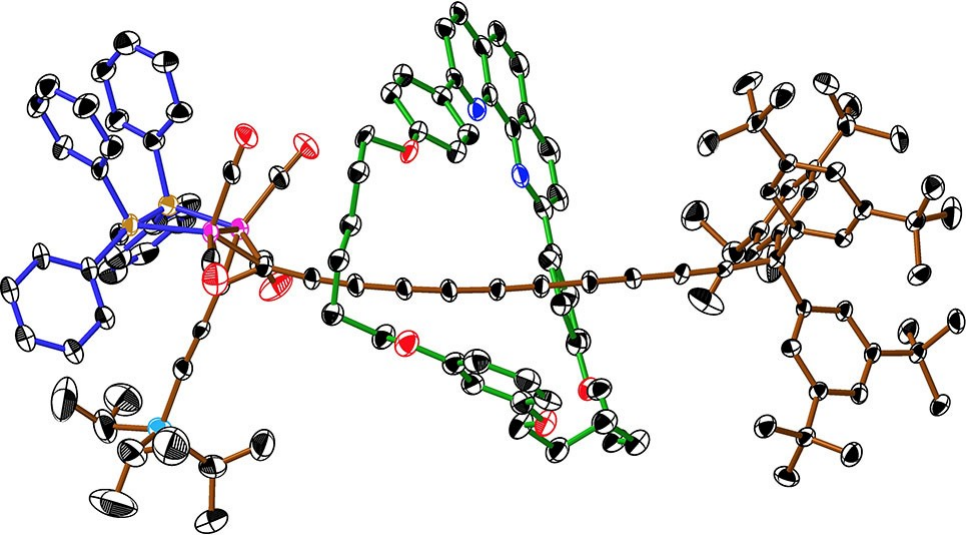
Crystal and molecular structure of [2]rotaxane **4⋅M1** (one of the four molecules in the asymmetric unit; displacement ellipsoids at 30 % probability, hydrogen atoms and minor component of disorder omitted for clarity).

The triisopropylsilyl (TIPS) protecting groups were removed from the [2]rotaxanes **4⋅M1** and **4⋅M2** using TBAF in wet THF, then the terminal alkynes **5⋅M1** and **5⋅M2** were subjected to Cu‐catalyzed oxidative homocoupling to obtain the [3]rotaxanes **6⋅**(**M1**)_2_ and **6⋅**(**M2**)_2_. To our surprise, the different macrocycles required different reaction conditions for this Glaser coupling step. Standard Glaser–Hay conditions (CuCl, TMEDA, CH_2_Cl_2_, O_2_) cleanly converted **5⋅M1** to [3]rotaxane **6⋅**(**M1**)_2_ in 90 % yield. However, the oxidative homocoupling of **5⋅M2** to afford the nanohoop [3]rotaxane **6⋅**(**M2**)_2_ was unexpectedly problematic. Standard Glaser–Hay conditions rapidly convert **5⋅M2** to unidentified by‐products, and we found that the free nanohoop **M2** is not stable under these conditions (CuCl, TMEDA, CH_2_Cl_2_, O_2_, 20 °C, 30 min). A variety of Cu^I^ and Cu^I^/Pd^0^ mixed catalyst systems were trialed, yet none yielded the expected product. However, successful coupling was observed when using 4,4′‐di‐*tert*‐butyl‐2,2′‐bipyridine instead of TMEDA under Glaser–Hay coupling conditions.^[6b]^ Warming to 30 °C significantly accelerated the reaction, compared with coupling at 20 °C (although it is still markedly slower than with TMEDA), and the [3]rotaxane **6⋅**(**M2**)_2_ was isolated in 77 % yield after 20 h.

The final polyyne [3]rotaxanes **1⋅**(**M1**)_2_ and **1⋅**(**M2**)_2_ were prepared by oxidative decomplexation of the corresponding masked [3]rotaxanes using iodine. Once again, the two rotaxanes **6⋅**(**M1**)_2_ and **6⋅**(**M2**)_2_ varied significantly in reactivity. In the case of **6⋅**(**M1**)_2_, unmasking proved capricious. Even after meticulous optimization of the reaction conditions, the polyyne rotaxane **1⋅**(**M1**)_2_ could only rarely be obtained in yields of 20–36 %. In contrast, treatment of [3]rotaxane **6⋅**(**M2**)_2_ with iodine in a 1 : 1 THF/MeCN reliably gave polyyne [3]rotaxane **1⋅**(**M2**)_2_ in 32 % isolated yield. Both [3]rotaxanes **1⋅**(**M1**)_2_ and **1⋅**(**M2**)_2_ are stable under ambient conditions, both as solids and in solution over a period of weeks (monitored by UV/Vis spectroscopy).

The ^1^H and ^13^C NMR spectra of the [3]rotaxanes **1⋅**(**M1**)_2_ and **1⋅**(**M2**)_2_ are similar to the sum of the spectra of their components (**1**+**M1** and **1**+**M2**, respectively; see Supporting Information, Figures S17, S24 and S25), indicating the absence of any strong interaction between the polyyne dumbbell and the macrocycles. The spectra of the nanohoop polyyne [3]rotaxane **1⋅**(**M2**)_2_ reveal that rotation of the *para*‐phenylene units of the threaded nanohoop **M2** is slow on the NMR timescale, making the two faces of the nanohoop chemically non‐equivalent. Thus 10 distinct *para*‐phenylene C−H environments are observed in the HSQC spectrum of **1⋅**(**M2**)_2_ (Figure 2), whereas the free nanohoop **M2** gives only 5 *para*‐phenylene CH signals.

**Figure 2:**
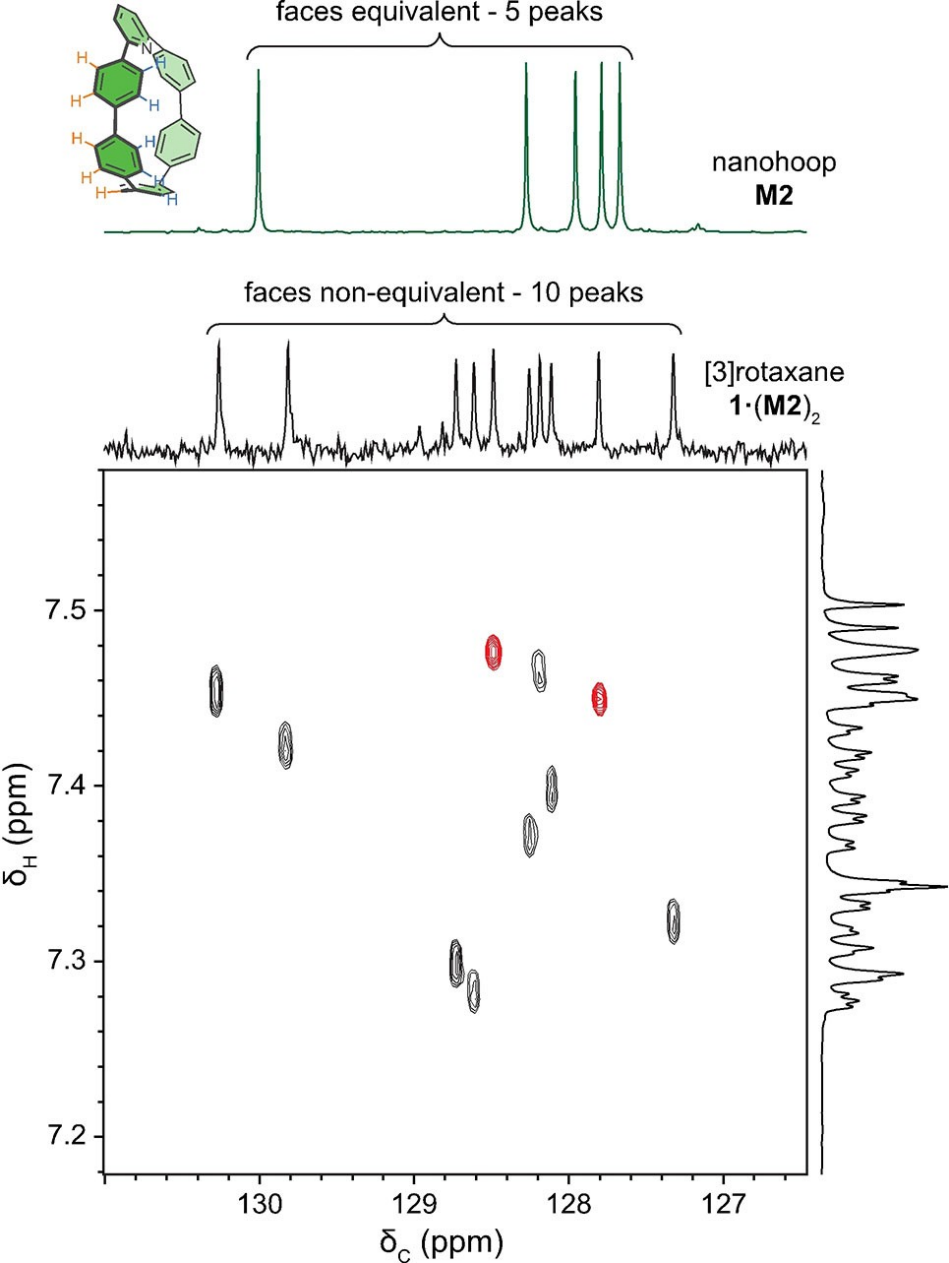
Top: Partial ^13^C NMR spectra of (green) the free nanohoop and (black) the nanohoop‐protected polyyne [3]rotaxane **1⋅**(**M2**)_2_. Bottom: High‐resolution HSQC spectrum showing C−H correlation for the chemically non‐equivalent *para*‐phenylene C−H signals. Cross peaks arising from the middle *para*‐phenylene, furthest away from the pyridine unit, have been colored red. The ^1^H reference spectrum has been diffusion edited to attenuate the overlapping CHCl_3_ resonance (CDCl_3_, 298 K, 700 MHz ^1^H frequency).

The UV/Vis absorption spectra of **1⋅**(**M1**)_2_ and **1⋅**(**M2**)_2_ (Figure 3) closely resemble the spectrum of the free dumbbell **1**, previously reported by Tykwinski et al.^[6a]^ The slight bathochromic shift in the spectra of the [3]rotaxanes (5 nm for **M1** and 7 nm for **M2**) is attributed to the different solvation environments in the [3]rotaxanes. Similar shifts have been reported in the UV/Vis spectra of other polyyne rotaxanes.^[8c]^ Nanohoop **M2** is known to be highly fluorescent,[^10^, ^16^] but its fluorescence is totally quenched in **1⋅**(**M2**)_2_ (see Supporting Information, Figure S27), probably via energy transfer to dark states of the polyyne.[^18^, ^19^] Thus, although the absorption spectra show only a minimal interaction between the macrocycle and the polyyne in the ground state, there is a significant interaction in the excited state.

**Figure 3:**
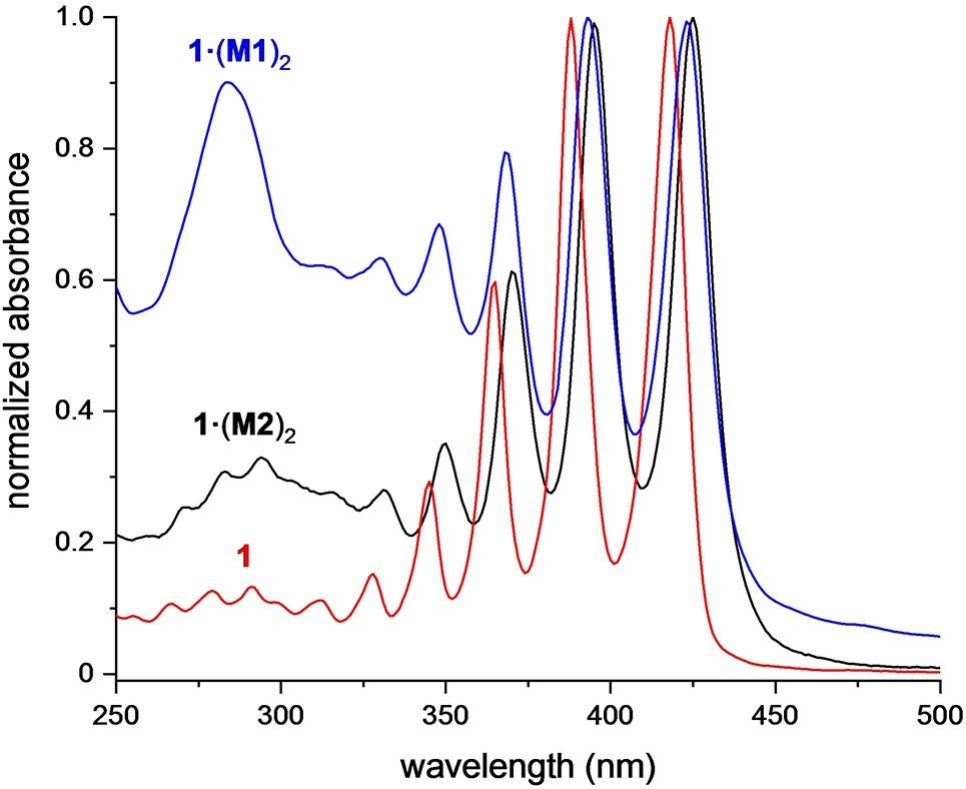
Normalized UV/Vis absorption spectra of polyyne **1** (red), phenanthroline [3]rotaxane **1⋅**(**M1**)_2_ (blue) and nanohoop [3]rotaxane **1⋅**(**M2**)_2_ (black), all as solutions in *n*‐hexane at 25 °C.

Next we tested whether the chemical stability of the C_28_ polyyne axle of **1** is enhanced by supramolecular encapsulation. Previously, differential scanning calorimetry (DSC) has been used to demonstrate a stability enhancement in some polyyne rotaxanes.^[8c]^ The problem with studying solid‐state stability is that it is influenced by unpredictable crystal packing effects. DSC analysis of **1** and **1⋅**(**M2**)_2_ showed that they decompose at similar temperatures (155 °C and 149 °C, respectively, see Supporting Information, Figure S37). We also investigated the stability of these compounds in solution. Oxygen‐free solutions of thread **1** and [3]rotaxanes **1⋅**(**M1**)_2_ and **1⋅**(**M2**)_2_ in decalin, at a concentration of about 1 μM, were heated to 80 °C in a silica cuvette and decomposition was monitored by UV/Vis spectroscopy. The sharp UV bands of the polyyne were found to decay exponentially, consistent with first‐order reaction kinetics (Figure 4). Fitting these data gave apparent first‐order rate constants of 0.092 s^−1^, 0.080 s^−1^ and 0.021 s^−1^ for the dumbbell **1** and the phenanthroline and nanohoop [3]rotaxanes **1⋅**(**M1**)_2_ and **1⋅**(**M2**)_2_, respectively. Experimental uncertainties associated with these measurements were estimated from repeat experiments at approximately 10 %. The minimal stability enhancement for **1⋅**(**M1**)_2_ may be attributed to the greater size and flexibility of the phenanthroline macrocycle, which does not effectively shield the polyyne. The tighter nanohoop in **1⋅**(**M2**)_2_ enhances the stability of the threaded polyyne by a factor of approximately 4.5.

**Figure 4:**
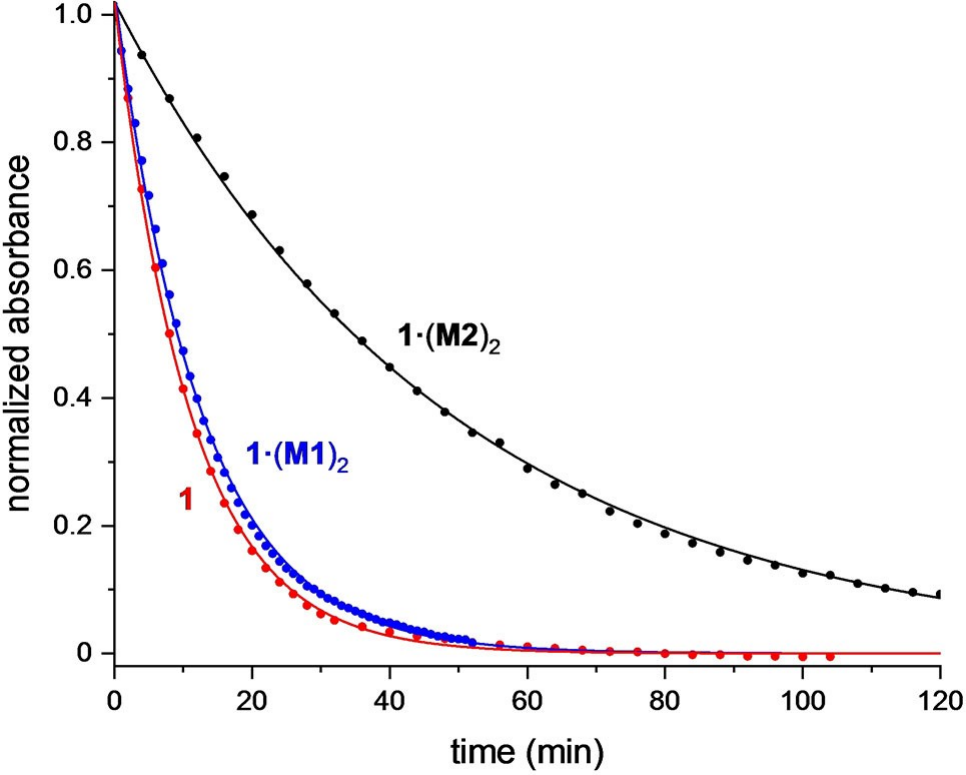
Thermal decomposition of the polyyne dumbbell **1** (red), phenanthroline [3]rotaxane **1⋅**(**M1**)_2_ (blue) and nanohoop [3]rotaxane **1⋅**(**M2**)_2_ (black) (decalin, 80 °C). The intensity of lowest energy band (418 nm, 423 nm and 425 nm for dumbbell **1** and [3]rotaxanes **1⋅**(**M1**)_2_ and **1⋅**(**M2**)_2_, respectively) was followed in each case. Data are fitted to a first‐order exponential decay; normalized absorbance=(*A*−*A*_f_ *)*/(*A*_0_−*A*_f_ *)*=exp(−*kt*), where *A*, *A*_0_ and *A*_f_ are the absorbance at time *t*, absorbance at *t*=0 and absorbance at *t*=∞, respectively, and *k* is the rate constant; see details in Supporting Information.

In summary, we have presented a new synthetic route to polyyne [3]rotaxanes, and we have shown that the size and shape of the macrocycle influence its ability to enhance the thermal stability of a threaded polyyne. Frauenrath et al. reported a [3]rotaxane consisting of a hexayne dumbbell threaded through two cyclodextrin rings, which also exhibited dramatic stability enhancement.^[20]^ Their synthesis used hydrophobic binding to promote threading, which required the [3]rotaxane to be prepared in aqueous solution. Active metal template coupling is a more versatile approach to polyyne rotaxanes, and the ability to prepare polyrotaxanes with cylindrical nanohoop macrocycles is a significant step towards the synthesis of encapsulated carbyne.

## Conflict of interest

The authors declare no conflict of interest.

## Supporting information

As a service to our authors and readers, this journal provides supporting information supplied by the authors. Such materials are peer reviewed and may be re‐organized for online delivery, but are not copy‐edited or typeset. Technical support issues arising from supporting information (other than missing files) should be addressed to the authors.

Supporting Information

Supporting Information

Supporting Information

## Data Availability

The data that support the findings of this study are available in the supplementary material of this article.
