# Supplementary material for: Polyyne [3]Rotaxanes: Synthesis via Dicobalt Carbonyl Complexes and Enhanced Stability
Source: Angew Chem Int Ed Engl. 2022 Jan 20;61(10):e202116897. doi: 10.1002/anie.202116897 (PMC9302669; doi:10.1002/anie.202116897)

checkCIF/PLATON report

Structure factors have been supplied for datablock(s) I

THIS REPORT IS FOR GUIDANCE ONLY. IF USED AS PART OF A REVIEW PROCEDURE FOR PUBLICATION, IT SHOULD NOT REPLACE THE EXPERTISE OF AN EXPERIENCED CRYSTALLOGRAPHIC REFEREE.

No syntax errors found. CIF dictionary Interpreting this report

Datablock: I

```
Bond precision:   C-C = 0.0100 A          Wavelength=1.54180

Cell:           a=23.1925(6)        b=28.6354(7)       c=39.0458(6)
                alpha=87.4974(16)   beta=83.2863(17)   gamma=76.621(2)
Temperature:    150 K
```

	Calculated	Reported
Volume	25050.9(10)	25050.9(10)
Space group	P -1	P -1
Hall group	-P 1	-P 1
Moiety formula	C95 H106 Co2 O4 P2 Si, C42 H42 N2 O4, 0.494(C H4 O) [+ solvent]	C137 H149 Co2 N2 O8 P2 Si1, 0.49(C H4 O)
Sum formula	C137.49 H149.97 Co2 N2 O8.49 P2 Si [+ solvent]	C137.49 H149.98 Co2 N2 O8.49 P2 Si1
Mr	2174.29	2174.40
Dx,g cm-3	1.153	1.153
Z	8	8
Mu (mm-1)	2.840	2.840
F000	9239.1	9239.1
F000’	9230.84	
h,k,lmax	23,28,39	23,28,38
Nref	53269	52527
Tmin,Tmax	0.550,0.893	0.720,0.890
Tmin’	0.324	

```
Correction method= # Reported T Limits: Tmin=0.720 Tmax=0.890
AbsCorr = MULTI-SCAN

Data completeness= 0.986            Theta(max)= 50.787
```

R(reflections)= 0.1035(28609)   wR2(reflections)= 0.3065(52400)

S = 0.982   Npar= 6331

The following ALERTS were generated. Each ALERT has the format
test-name_ALERT_alert-type_alert-level.
Click on the hyperlinks for more details of the test.

Alert level A

THETM01_ALERT_3_A The value of sine(theta_max)/wavelength is less than 0.550
Calculated sin(theta_max)/wavelength = 0.5025

Author Response: The structure consists of a very large molecule with no strong structure directing interactions. As a result it forms poor qulity crystals in this case with four molecules in the asymmetric unit and a considerable amount of disorder at the periphery. As a result there is very little in the way of high angle data. Despite this, there is little doubt that the connectivity is correct and the results herein confirm the interlocked nature of the compound as described.

PLAT023_ALERT_3_A Resolution (too) Low [sin(theta)/Lambda < 0.6].. 0.50 Ang-1

Author Response: The structure consists of a very large molecule with no strong structure directing interactions. As a result it forms poor qulity crystals in this case with four molecules in the asymmetric unit and a considerable amount of disorder at the periphery. As a result the data do not extend beyond medium resolution. Despite this, there is little doubt that the connectivity is correct and the results herein confirm the interlocked nature of the compound as described.

PLAT027_ALERT_3_A _diffrn_reflns_theta_full value (too) Low ...... 49.26 Degree

Author Response: The structure consists of a very large molecule with no strong structure directing interactions. As a result it forms poor qulity crystals in this case with four molecules in the asymmetric unit and a considerable amount of disorder at the periphery. As a result there is very little in the way of high angle data. For this reason, the completness statistics etc. were calulated for much lower value than is usual. Despite the poor qulity of the data, there is little doubt that the connectivity is correct and the results herein confirm the interlocked nature of the compound as described.

PLAT904_ALERT_1_A Number of Reflections is < Number of Parameters 3508

Alert level C

PLAT084_ALERT_3_C High wR2 Value (i.e. > 0.25) .... 0.31 Report

```
PLAT220_ALERT_2_C NonSolvent   Resd 1  C   Ueq(max)/Ueq(min) Range        4.2 Ratio
PLAT221_ALERT_2_C Solv./Anion  Resd 2  C   Ueq(max)/Ueq(min) Range        4.4 Ratio
PLAT221_ALERT_2_C Solv./Anion  Resd 3  C   Ueq(max)/Ueq(min) Range        5.2 Ratio
PLAT221_ALERT_2_C Solv./Anion  Resd 4  C   Ueq(max)/Ueq(min) Range        4.5 Ratio
PLAT222_ALERT_3_C NonSolvent Resd 1  H   Uiso(max)/Uiso(min) Range        4.1 Ratio
PLAT223_ALERT_4_C Solv./Anion  Resd 2  H   Ueq(max)/Ueq(min) Range        4.3 Ratio
PLAT223_ALERT_4_C Solv./Anion  Resd 3  H   Ueq(max)/Ueq(min) Range        4.7 Ratio
PLAT223_ALERT_4_C Solv./Anion  Resd 4  H   Ueq(max)/Ueq(min) Range        4.5 Ratio
PLAT241_ALERT_2_C High   'MainMol' Ueq as Compared to Neighbors of       C328 Check
PLAT242_ALERT_2_C Low    'MainMol' Ueq as Compared to Neighbors of       C716 Check
PLAT242_ALERT_2_C Low    'MainMol' Ueq as Compared to Neighbors of       C724 Check
PLAT242_ALERT_2_C Low    'MainMol' Ueq as Compared to Neighbors of       C457 Check
PLAT250_ALERT_2_C Large U3/U1 Ratio for Average U(i,j) Tensor ....        2.5 Note
PLAT250_ALERT_2_C Large U3/U1 Ratio for Average U(i,j) Tensor ....        2.4 Note
PLAT260_ALERT_2_C Large Average Ueq of Residue Including      O607      0.115 Check
PLAT260_ALERT_2_C Large Average Ueq of Residue Including      O407      0.124 Check
PLAT260_ALERT_2_C Large Average Ueq of Residue Including     O1407      0.126 Check
PLAT260_ALERT_2_C Large Average Ueq of Residue Including      O801      0.111 Check
PLAT260_ALERT_2_C Large Average Ueq of Residue Including      O803      0.183 Check
PLAT331_ALERT_2_C Small Aver Phenyl C-C Dist C724     --C729     .       1.37 Ang.
PLAT331_ALERT_2_C Small Aver Phenyl C-C Dist C518     --C523     .       1.37 Ang.
PLAT334_ALERT_2_C Small Aver. Benzene C-C Dist C601     -C606            1.37 Ang.
PLAT341_ALERT_3_C Low Bond Precision on  C-C Bonds ...............    0.00997 Ang.
PLAT373_ALERT_2_C Long   C(sp)-C(sp)   Bond  C294     - C295     .       1.38 Ang.
PLAT373_ALERT_2_C Long   C(sp)-C(sp)   Bond  C296     - C297     .       1.36 Ang.
PLAT373_ALERT_2_C Long   C(sp)-C(sp)   Bond  C298     - C299     .       1.36 Ang.
PLAT373_ALERT_2_C Long   C(sp)-C(sp)   Bond  C694     - C695     .       1.36 Ang.
PLAT373_ALERT_2_C Long   C(sp)-C(sp)   Bond  C696     - C697     .       1.36 Ang.
PLAT373_ALERT_2_C Long   C(sp)-C(sp)   Bond  C700     - C701     .       1.36 Ang.
PLAT373_ALERT_2_C Long   C(sp)-C(sp)   Bond  C494     - C495     .       1.36 Ang.
PLAT373_ALERT_2_C Long   C(sp)-C(sp)   Bond  C496     - C497     .       1.37 Ang.
PLAT373_ALERT_2_C Long   C(sp)-C(sp)   Bond  C498     - C499     .       1.37 Ang.
```

Alert level G

```
FORMU01_ALERT_1_G  There is a discrepancy between the atom counts in the
            _chemical_formula_sum and _chemical_formula_moiety. This is
            usually due to the moiety formula being in the wrong format.
            Atom count from _chemical_formula_sum:   C137.4899 H149.98 Co2 N2 O8.4
            Atom count from _chemical_formula_moiety:C137.4900 H150.9600 Co2 N2 O8
CELLZ01_ALERT_1_G Difference between formula and atom_site contents detected.
CELLZ01_ALERT_1_G ALERT: check formula stoichiometry or atom site occupancies.
           From the CIF: _cell_formula_units_Z    8
           From the CIF: _chemical_formula_sum  C137.49 H149.98 Co2 N2 O8.49 P2 Si
           TEST: Compare cell contents of formula and atom_site data

           atom    Z*formula  cif sites diff
           C       1099.92   1099.95   -0.03
           H       1199.84   1199.80    0.04
           Co        16.00     16.00    0.00
           N         16.00     16.00    0.00
           O         67.92     67.95   -0.03
           P         16.00     16.00    0.00
           Si         8.00      8.00    0.00
PLAT002_ALERT_2_G Number of Distance or Angle Restraints on AtSite        562 Note
PLAT003_ALERT_2_G Number of Uiso or Uij Restrained non-H Atoms ...        754 Report
PLAT007_ALERT_5_G Number of Unrefined Donor-H Atoms .............           2 Report
```

```
PLAT041_ALERT_1_G Calc. and Reported SumFormula    Strings  Differ     Please Check
PLAT042_ALERT_1_G Calc. and Reported Moiety Formula Strings Differ     Please Check
PLAT072_ALERT_2_G SHELXL First  Parameter in WGHT  Unusually Large       0.13 Report
PLAT175_ALERT_4_G The CIF-Embedded .res File Contains SAME Records          6 Report
PLAT177_ALERT_4_G The CIF-Embedded .res File Contains DELU Records          6 Report
PLAT178_ALERT_4_G The CIF-Embedded .res File Contains SIMU Records          6 Report
PLAT300_ALERT_4_G Atom Site Occupancy of Si344      Constrained at     0.7013 Check
PLAT300_ALERT_4_G Atom Site Occupancy of Si1344     Constrained at     0.2987 Check
PLAT300_ALERT_4_G Atom Site Occupancy of C1257      Constrained at     0.5066 Check
PLAT300_ALERT_4_G Atom Site Occupancy of C1258      Constrained at     0.5066 Check
PLAT300_ALERT_4_G Atom Site Occupancy of C1259      Constrained at     0.5066 Check
PLAT300_ALERT_4_G Atom Site Occupancy of C1260      Constrained at     0.5066 Check
PLAT300_ALERT_4_G Atom Site Occupancy of C1265      Constrained at     0.5105 Check
PLAT300_ALERT_4_G Atom Site Occupancy of C1266      Constrained at     0.5105 Check
PLAT300_ALERT_4_G Atom Site Occupancy of C1267      Constrained at     0.5105 Check
PLAT300_ALERT_4_G Atom Site Occupancy of C1268      Constrained at     0.5105 Check
PLAT300_ALERT_4_G Atom Site Occupancy of C1269      Constrained at     0.5105 Check
PLAT300_ALERT_4_G Atom Site Occupancy of C1270      Constrained at     0.5105 Check
PLAT300_ALERT_4_G Atom Site Occupancy of C1271      Constrained at     0.5105 Check
PLAT300_ALERT_4_G Atom Site Occupancy of C1272      Constrained at     0.5105 Check
PLAT300_ALERT_4_G Atom Site Occupancy of C1273      Constrained at     0.5105 Check
PLAT300_ALERT_4_G Atom Site Occupancy of C1274      Constrained at     0.5105 Check
PLAT300_ALERT_4_G Atom Site Occupancy of C1275      Constrained at     0.5105 Check
PLAT300_ALERT_4_G Atom Site Occupancy of C1276      Constrained at     0.5105 Check
PLAT300_ALERT_4_G Atom Site Occupancy of C1277      Constrained at     0.5105 Check
PLAT300_ALERT_4_G Atom Site Occupancy of C1278      Constrained at     0.5105 Check
PLAT300_ALERT_4_G Atom Site Occupancy of C342       Constrained at     0.7013 Check
PLAT300_ALERT_4_G Atom Site Occupancy of C343       Constrained at     0.7013 Check
PLAT300_ALERT_4_G Atom Site Occupancy of C345       Constrained at     0.7013 Check
PLAT300_ALERT_4_G Atom Site Occupancy of C346       Constrained at     0.7013 Check
PLAT300_ALERT_4_G Atom Site Occupancy of C347       Constrained at     0.7013 Check
PLAT300_ALERT_4_G Atom Site Occupancy of C348       Constrained at     0.7013 Check
PLAT300_ALERT_4_G Atom Site Occupancy of C349       Constrained at     0.7013 Check
PLAT300_ALERT_4_G Atom Site Occupancy of C350       Constrained at     0.7013 Check
PLAT300_ALERT_4_G Atom Site Occupancy of C351       Constrained at     0.7013 Check
PLAT300_ALERT_4_G Atom Site Occupancy of C352       Constrained at     0.7013 Check
PLAT300_ALERT_4_G Atom Site Occupancy of C353       Constrained at     0.7013 Check
PLAT300_ALERT_4_G Atom Site Occupancy of C1342      Constrained at     0.2987 Check
PLAT300_ALERT_4_G Atom Site Occupancy of C1343      Constrained at     0.2987 Check
PLAT300_ALERT_4_G Atom Site Occupancy of C1345      Constrained at     0.2987 Check
PLAT300_ALERT_4_G Atom Site Occupancy of C1346      Constrained at     0.2987 Check
PLAT300_ALERT_4_G Atom Site Occupancy of C1347      Constrained at     0.2987 Check
PLAT300_ALERT_4_G Atom Site Occupancy of C1348      Constrained at     0.2987 Check
PLAT300_ALERT_4_G Atom Site Occupancy of C1349      Constrained at     0.2987 Check
PLAT300_ALERT_4_G Atom Site Occupancy of C1350      Constrained at     0.2987 Check
PLAT300_ALERT_4_G Atom Site Occupancy of C1351      Constrained at     0.2987 Check
PLAT300_ALERT_4_G Atom Site Occupancy of C1352      Constrained at     0.2987 Check
PLAT300_ALERT_4_G Atom Site Occupancy of C1353      Constrained at     0.2987 Check
PLAT300_ALERT_4_G Atom Site Occupancy of C257       Constrained at     0.4934 Check
PLAT300_ALERT_4_G Atom Site Occupancy of C258       Constrained at     0.4934 Check
PLAT300_ALERT_4_G Atom Site Occupancy of C259       Constrained at     0.4934 Check
PLAT300_ALERT_4_G Atom Site Occupancy of C260       Constrained at     0.4934 Check
PLAT300_ALERT_4_G Atom Site Occupancy of C265       Constrained at     0.4895 Check
PLAT300_ALERT_4_G Atom Site Occupancy of C266       Constrained at     0.4895 Check
PLAT300_ALERT_4_G Atom Site Occupancy of C267       Constrained at     0.4895 Check
PLAT300_ALERT_4_G Atom Site Occupancy of C268       Constrained at     0.4895 Check
PLAT300_ALERT_4_G Atom Site Occupancy of C269       Constrained at     0.4895 Check
```

```
PLAT300_ALERT_4_G Atom Site Occupancy of C270       Constrained at     0.4895 Check
PLAT300_ALERT_4_G Atom Site Occupancy of C271       Constrained at     0.4895 Check
PLAT300_ALERT_4_G Atom Site Occupancy of C272       Constrained at     0.4895 Check
PLAT300_ALERT_4_G Atom Site Occupancy of C273       Constrained at     0.4895 Check
PLAT300_ALERT_4_G Atom Site Occupancy of C274       Constrained at     0.4895 Check
PLAT300_ALERT_4_G Atom Site Occupancy of C275       Constrained at     0.4895 Check
PLAT300_ALERT_4_G Atom Site Occupancy of C276       Constrained at     0.4895 Check
PLAT300_ALERT_4_G Atom Site Occupancy of C277       Constrained at     0.4895 Check
PLAT300_ALERT_4_G Atom Site Occupancy of C278       Constrained at     0.4895 Check
PLAT300_ALERT_4_G Atom Site Occupancy of H3451      Constrained at     0.7013 Check
PLAT300_ALERT_4_G Atom Site Occupancy of H3461      Constrained at     0.7013 Check
PLAT300_ALERT_4_G Atom Site Occupancy of H3462      Constrained at     0.7013 Check
PLAT300_ALERT_4_G Atom Site Occupancy of H3463      Constrained at     0.7013 Check
PLAT300_ALERT_4_G Atom Site Occupancy of H3471      Constrained at     0.7013 Check
PLAT300_ALERT_4_G Atom Site Occupancy of H3472      Constrained at     0.7013 Check
PLAT300_ALERT_4_G Atom Site Occupancy of H3473      Constrained at     0.7013 Check
PLAT300_ALERT_4_G Atom Site Occupancy of H3481      Constrained at     0.7013 Check
PLAT300_ALERT_4_G Atom Site Occupancy of H3491      Constrained at     0.7013 Check
PLAT300_ALERT_4_G Atom Site Occupancy of H3492      Constrained at     0.7013 Check
PLAT300_ALERT_4_G Atom Site Occupancy of H3493      Constrained at     0.7013 Check
PLAT300_ALERT_4_G Atom Site Occupancy of H3501      Constrained at     0.7013 Check
PLAT300_ALERT_4_G Atom Site Occupancy of H3502      Constrained at     0.7013 Check
PLAT300_ALERT_4_G Atom Site Occupancy of H3503      Constrained at     0.7013 Check
PLAT300_ALERT_4_G Atom Site Occupancy of H3511      Constrained at     0.7013 Check
PLAT300_ALERT_4_G Atom Site Occupancy of H3521      Constrained at     0.7013 Check
PLAT300_ALERT_4_G Atom Site Occupancy of H3522      Constrained at     0.7013 Check
PLAT300_ALERT_4_G Atom Site Occupancy of H3523      Constrained at     0.7013 Check
PLAT300_ALERT_4_G Atom Site Occupancy of H3531      Constrained at     0.7013 Check
PLAT300_ALERT_4_G Atom Site Occupancy of H3532      Constrained at     0.7013 Check
PLAT300_ALERT_4_G Atom Site Occupancy of H3533      Constrained at     0.7013 Check
PLAT300_ALERT_4_G Atom Site Occupancy of H12581     Constrained at     0.5066 Check
PLAT300_ALERT_4_G Atom Site Occupancy of H12582     Constrained at     0.5066 Check
PLAT300_ALERT_4_G Atom Site Occupancy of H12583     Constrained at     0.5066 Check
PLAT300_ALERT_4_G Atom Site Occupancy of H12591     Constrained at     0.5066 Check
PLAT300_ALERT_4_G Atom Site Occupancy of H12592     Constrained at     0.5066 Check
PLAT300_ALERT_4_G Atom Site Occupancy of H12594     Constrained at     0.5066 Check
PLAT300_ALERT_4_G Atom Site Occupancy of H12601     Constrained at     0.5066 Check
PLAT300_ALERT_4_G Atom Site Occupancy of H12602     Constrained at     0.5066 Check
PLAT300_ALERT_4_G Atom Site Occupancy of H12603     Constrained at     0.5066 Check
PLAT300_ALERT_4_G Atom Site Occupancy of H12661     Constrained at     0.5105 Check
PLAT300_ALERT_4_G Atom Site Occupancy of H12681     Constrained at     0.5105 Check
PLAT300_ALERT_4_G Atom Site Occupancy of H12701     Constrained at     0.5105 Check
PLAT300_ALERT_4_G Atom Site Occupancy of H12721     Constrained at     0.5105 Check
PLAT300_ALERT_4_G Atom Site Occupancy of H12722     Constrained at     0.5105 Check
PLAT300_ALERT_4_G Atom Site Occupancy of H12723     Constrained at     0.5105 Check
PLAT300_ALERT_4_G Atom Site Occupancy of H12731     Constrained at     0.5105 Check
PLAT300_ALERT_4_G Atom Site Occupancy of H12732     Constrained at     0.5105 Check
PLAT300_ALERT_4_G Atom Site Occupancy of H12733     Constrained at     0.5105 Check
PLAT300_ALERT_4_G Atom Site Occupancy of H12741     Constrained at     0.5105 Check
PLAT300_ALERT_4_G Atom Site Occupancy of H12742     Constrained at     0.5105 Check
PLAT300_ALERT_4_G Atom Site Occupancy of H12743     Constrained at     0.5105 Check
PLAT300_ALERT_4_G Atom Site Occupancy of H12761     Constrained at     0.5105 Check
PLAT300_ALERT_4_G Atom Site Occupancy of H12762     Constrained at     0.5105 Check
PLAT300_ALERT_4_G Atom Site Occupancy of H12764     Constrained at     0.5105 Check
PLAT300_ALERT_4_G Atom Site Occupancy of H12771     Constrained at     0.5105 Check
PLAT300_ALERT_4_G Atom Site Occupancy of H12772     Constrained at     0.5105 Check
PLAT300_ALERT_4_G Atom Site Occupancy of H12773     Constrained at     0.5105 Check
```

```
PLAT300_ALERT_4_G Atom Site Occupancy of H12781     Constrained at     0.5105 Check
PLAT300_ALERT_4_G Atom Site Occupancy of H12782     Constrained at     0.5105 Check
PLAT300_ALERT_4_G Atom Site Occupancy of H12783     Constrained at     0.5105 Check
PLAT300_ALERT_4_G Atom Site Occupancy of H2581      Constrained at     0.4934 Check
PLAT300_ALERT_4_G Atom Site Occupancy of H2582      Constrained at     0.4934 Check
PLAT300_ALERT_4_G Atom Site Occupancy of H2583      Constrained at     0.4934 Check
PLAT300_ALERT_4_G Atom Site Occupancy of H2591      Constrained at     0.4934 Check
PLAT300_ALERT_4_G Atom Site Occupancy of H2592      Constrained at     0.4934 Check
PLAT300_ALERT_4_G Atom Site Occupancy of H2593      Constrained at     0.4934 Check
PLAT300_ALERT_4_G Atom Site Occupancy of H2601      Constrained at     0.4934 Check
PLAT300_ALERT_4_G Atom Site Occupancy of H2602      Constrained at     0.4934 Check
PLAT300_ALERT_4_G Atom Site Occupancy of H2603      Constrained at     0.4934 Check
PLAT300_ALERT_4_G Atom Site Occupancy of H2661      Constrained at     0.4895 Check
PLAT300_ALERT_4_G Atom Site Occupancy of H2681      Constrained at     0.4895 Check
PLAT300_ALERT_4_G Atom Site Occupancy of H2701      Constrained at     0.4895 Check
PLAT300_ALERT_4_G Atom Site Occupancy of H2721      Constrained at     0.4895 Check
PLAT300_ALERT_4_G Atom Site Occupancy of H2722      Constrained at     0.4895 Check
PLAT300_ALERT_4_G Atom Site Occupancy of H2723      Constrained at     0.4895 Check
PLAT300_ALERT_4_G Atom Site Occupancy of H2731      Constrained at     0.4895 Check
PLAT300_ALERT_4_G Atom Site Occupancy of H2732      Constrained at     0.4895 Check
PLAT300_ALERT_4_G Atom Site Occupancy of H2733      Constrained at     0.4895 Check
PLAT300_ALERT_4_G Atom Site Occupancy of H2741      Constrained at     0.4895 Check
PLAT300_ALERT_4_G Atom Site Occupancy of H2742      Constrained at     0.4895 Check
PLAT300_ALERT_4_G Atom Site Occupancy of H2743      Constrained at     0.4895 Check
PLAT300_ALERT_4_G Atom Site Occupancy of H2761      Constrained at     0.4895 Check
PLAT300_ALERT_4_G Atom Site Occupancy of H2762      Constrained at     0.4895 Check
PLAT300_ALERT_4_G Atom Site Occupancy of H2763      Constrained at     0.4895 Check
PLAT300_ALERT_4_G Atom Site Occupancy of H2771      Constrained at     0.4895 Check
PLAT300_ALERT_4_G Atom Site Occupancy of H2772      Constrained at     0.4895 Check
PLAT300_ALERT_4_G Atom Site Occupancy of H2773      Constrained at     0.4895 Check
PLAT300_ALERT_4_G Atom Site Occupancy of H2781      Constrained at     0.4895 Check
PLAT300_ALERT_4_G Atom Site Occupancy of H2782      Constrained at     0.4895 Check
PLAT300_ALERT_4_G Atom Site Occupancy of H2783      Constrained at     0.4895 Check
PLAT300_ALERT_4_G Atom Site Occupancy of H13451     Constrained at     0.2987 Check
PLAT300_ALERT_4_G Atom Site Occupancy of H13461     Constrained at     0.2987 Check
PLAT300_ALERT_4_G Atom Site Occupancy of H13462     Constrained at     0.2987 Check
PLAT300_ALERT_4_G Atom Site Occupancy of H13463     Constrained at     0.2987 Check
PLAT300_ALERT_4_G Atom Site Occupancy of H13471     Constrained at     0.2987 Check
PLAT300_ALERT_4_G Atom Site Occupancy of H13472     Constrained at     0.2987 Check
PLAT300_ALERT_4_G Atom Site Occupancy of H13473     Constrained at     0.2987 Check
PLAT300_ALERT_4_G Atom Site Occupancy of H13481     Constrained at     0.2987 Check
PLAT300_ALERT_4_G Atom Site Occupancy of H13491     Constrained at     0.2987 Check
PLAT300_ALERT_4_G Atom Site Occupancy of H13492     Constrained at     0.2987 Check
PLAT300_ALERT_4_G Atom Site Occupancy of H13493     Constrained at     0.2987 Check
PLAT300_ALERT_4_G Atom Site Occupancy of H13502     Constrained at     0.2987 Check
PLAT300_ALERT_4_G Atom Site Occupancy of H13503     Constrained at     0.2987 Check
PLAT300_ALERT_4_G Atom Site Occupancy of H13504     Constrained at     0.2987 Check
PLAT300_ALERT_4_G Atom Site Occupancy of H13511     Constrained at     0.2987 Check
PLAT300_ALERT_4_G Atom Site Occupancy of H13521     Constrained at     0.2987 Check
PLAT300_ALERT_4_G Atom Site Occupancy of H13522     Constrained at     0.2987 Check
PLAT300_ALERT_4_G Atom Site Occupancy of H13523     Constrained at     0.2987 Check
PLAT300_ALERT_4_G Atom Site Occupancy of H13531     Constrained at     0.2987 Check
PLAT300_ALERT_4_G Atom Site Occupancy of H13532     Constrained at     0.2987 Check
PLAT300_ALERT_4_G Atom Site Occupancy of H13533     Constrained at     0.2987 Check
PLAT300_ALERT_4_G Atom Site Occupancy of Si744      Constrained at     0.5483 Check
PLAT300_ALERT_4_G Atom Site Occupancy of Si1744     Constrained at     0.4517 Check
PLAT300_ALERT_4_G Atom Site Occupancy of C1672      Constrained at     0.7489 Check
```

```
PLAT300_ALERT_4_G Atom Site Occupancy of C1673      Constrained at     0.7489 Check
PLAT300_ALERT_4_G Atom Site Occupancy of C1674      Constrained at     0.7489 Check
PLAT300_ALERT_4_G Atom Site Occupancy of C1686      Constrained at     0.5766 Check
PLAT300_ALERT_4_G Atom Site Occupancy of C1687      Constrained at     0.5766 Check
PLAT300_ALERT_4_G Atom Site Occupancy of C1688      Constrained at     0.5766 Check
PLAT300_ALERT_4_G Atom Site Occupancy of C689       Constrained at     0.5866 Check
PLAT300_ALERT_4_G Atom Site Occupancy of C690       Constrained at     0.5866 Check
PLAT300_ALERT_4_G Atom Site Occupancy of C691       Constrained at     0.5866 Check
PLAT300_ALERT_4_G Atom Site Occupancy of C692       Constrained at     0.5866 Check
PLAT300_ALERT_4_G Atom Site Occupancy of C742       Constrained at     0.5483 Check
PLAT300_ALERT_4_G Atom Site Occupancy of C743       Constrained at     0.5483 Check
PLAT300_ALERT_4_G Atom Site Occupancy of C745       Constrained at     0.5483 Check
PLAT300_ALERT_4_G Atom Site Occupancy of C746       Constrained at     0.5483 Check
PLAT300_ALERT_4_G Atom Site Occupancy of C747       Constrained at     0.5483 Check
PLAT300_ALERT_4_G Atom Site Occupancy of C748       Constrained at     0.5483 Check
PLAT300_ALERT_4_G Atom Site Occupancy of C749       Constrained at     0.5483 Check
PLAT300_ALERT_4_G Atom Site Occupancy of C750       Constrained at     0.5483 Check
PLAT300_ALERT_4_G Atom Site Occupancy of C751       Constrained at     0.5483 Check
PLAT300_ALERT_4_G Atom Site Occupancy of C752       Constrained at     0.5483 Check
PLAT300_ALERT_4_G Atom Site Occupancy of C753       Constrained at     0.5483 Check
PLAT300_ALERT_4_G Atom Site Occupancy of C1689      Constrained at     0.4134 Check
PLAT300_ALERT_4_G Atom Site Occupancy of C1690      Constrained at     0.4134 Check
PLAT300_ALERT_4_G Atom Site Occupancy of C1691      Constrained at     0.4134 Check
PLAT300_ALERT_4_G Atom Site Occupancy of C1692      Constrained at     0.4134 Check
PLAT300_ALERT_4_G Atom Site Occupancy of C1742      Constrained at     0.4517 Check
PLAT300_ALERT_4_G Atom Site Occupancy of C1743      Constrained at     0.4517 Check
PLAT300_ALERT_4_G Atom Site Occupancy of C1745      Constrained at     0.4517 Check
PLAT300_ALERT_4_G Atom Site Occupancy of C1746      Constrained at     0.4517 Check
PLAT300_ALERT_4_G Atom Site Occupancy of C1747      Constrained at     0.4517 Check
PLAT300_ALERT_4_G Atom Site Occupancy of C1748      Constrained at     0.4517 Check
PLAT300_ALERT_4_G Atom Site Occupancy of C1749      Constrained at     0.4517 Check
PLAT300_ALERT_4_G Atom Site Occupancy of C1750      Constrained at     0.4517 Check
PLAT300_ALERT_4_G Atom Site Occupancy of C1751      Constrained at     0.4517 Check
PLAT300_ALERT_4_G Atom Site Occupancy of C1752      Constrained at     0.4517 Check
PLAT300_ALERT_4_G Atom Site Occupancy of C1753      Constrained at     0.4517 Check
PLAT300_ALERT_4_G Atom Site Occupancy of C672       Constrained at     0.2511 Check
PLAT300_ALERT_4_G Atom Site Occupancy of C673       Constrained at     0.2511 Check
PLAT300_ALERT_4_G Atom Site Occupancy of C674       Constrained at     0.2511 Check
PLAT300_ALERT_4_G Atom Site Occupancy of C686       Constrained at     0.4234 Check
PLAT300_ALERT_4_G Atom Site Occupancy of C687       Constrained at     0.4234 Check
PLAT300_ALERT_4_G Atom Site Occupancy of C688       Constrained at     0.4234 Check
PLAT300_ALERT_4_G Atom Site Occupancy of H6901      Constrained at     0.5866 Check
PLAT300_ALERT_4_G Atom Site Occupancy of H6902      Constrained at     0.5866 Check
PLAT300_ALERT_4_G Atom Site Occupancy of H6903      Constrained at     0.5866 Check
PLAT300_ALERT_4_G Atom Site Occupancy of H6911      Constrained at     0.5866 Check
PLAT300_ALERT_4_G Atom Site Occupancy of H6912      Constrained at     0.5866 Check
PLAT300_ALERT_4_G Atom Site Occupancy of H6913      Constrained at     0.5866 Check
PLAT300_ALERT_4_G Atom Site Occupancy of H6921      Constrained at     0.5866 Check
PLAT300_ALERT_4_G Atom Site Occupancy of H6922      Constrained at     0.5866 Check
PLAT300_ALERT_4_G Atom Site Occupancy of H6923      Constrained at     0.5866 Check
PLAT300_ALERT_4_G Atom Site Occupancy of H7451      Constrained at     0.5483 Check
PLAT300_ALERT_4_G Atom Site Occupancy of H7461      Constrained at     0.5483 Check
PLAT300_ALERT_4_G Atom Site Occupancy of H7462      Constrained at     0.5483 Check
PLAT300_ALERT_4_G Atom Site Occupancy of H7463      Constrained at     0.5483 Check
PLAT300_ALERT_4_G Atom Site Occupancy of H7471      Constrained at     0.5483 Check
PLAT300_ALERT_4_G Atom Site Occupancy of H7472      Constrained at     0.5483 Check
PLAT300_ALERT_4_G Atom Site Occupancy of H7473      Constrained at     0.5483 Check
```

```
PLAT300_ALERT_4_G Atom Site Occupancy of H7481      Constrained at     0.5483 Check
PLAT300_ALERT_4_G Atom Site Occupancy of H7491      Constrained at     0.5483 Check
PLAT300_ALERT_4_G Atom Site Occupancy of H7492      Constrained at     0.5483 Check
PLAT300_ALERT_4_G Atom Site Occupancy of H7493      Constrained at     0.5483 Check
PLAT300_ALERT_4_G Atom Site Occupancy of H7501      Constrained at     0.5483 Check
PLAT300_ALERT_4_G Atom Site Occupancy of H7502      Constrained at     0.5483 Check
PLAT300_ALERT_4_G Atom Site Occupancy of H7503      Constrained at     0.5483 Check
PLAT300_ALERT_4_G Atom Site Occupancy of H7511      Constrained at     0.5483 Check
PLAT300_ALERT_4_G Atom Site Occupancy of H7521      Constrained at     0.5483 Check
PLAT300_ALERT_4_G Atom Site Occupancy of H7523      Constrained at     0.5483 Check
PLAT300_ALERT_4_G Atom Site Occupancy of H7524      Constrained at     0.5483 Check
PLAT300_ALERT_4_G Atom Site Occupancy of H7531      Constrained at     0.5483 Check
PLAT300_ALERT_4_G Atom Site Occupancy of H7532      Constrained at     0.5483 Check
PLAT300_ALERT_4_G Atom Site Occupancy of H7533      Constrained at     0.5483 Check
PLAT300_ALERT_4_G Atom Site Occupancy of H16721     Constrained at     0.7489 Check
PLAT300_ALERT_4_G Atom Site Occupancy of H16722     Constrained at     0.7489 Check
PLAT300_ALERT_4_G Atom Site Occupancy of H16723     Constrained at     0.7489 Check
PLAT300_ALERT_4_G Atom Site Occupancy of H16731     Constrained at     0.7489 Check
PLAT300_ALERT_4_G Atom Site Occupancy of H16732     Constrained at     0.7489 Check
PLAT300_ALERT_4_G Atom Site Occupancy of H16734     Constrained at     0.7489 Check
PLAT300_ALERT_4_G Atom Site Occupancy of H16741     Constrained at     0.7489 Check
PLAT300_ALERT_4_G Atom Site Occupancy of H16742     Constrained at     0.7489 Check
PLAT300_ALERT_4_G Atom Site Occupancy of H16743     Constrained at     0.7489 Check
PLAT300_ALERT_4_G Atom Site Occupancy of H16861     Constrained at     0.5766 Check
PLAT300_ALERT_4_G Atom Site Occupancy of H16862     Constrained at     0.5766 Check
PLAT300_ALERT_4_G Atom Site Occupancy of H16863     Constrained at     0.5766 Check
PLAT300_ALERT_4_G Atom Site Occupancy of H16871     Constrained at     0.5766 Check
PLAT300_ALERT_4_G Atom Site Occupancy of H16872     Constrained at     0.5766 Check
PLAT300_ALERT_4_G Atom Site Occupancy of H16873     Constrained at     0.5766 Check
PLAT300_ALERT_4_G Atom Site Occupancy of H16881     Constrained at     0.5766 Check
PLAT300_ALERT_4_G Atom Site Occupancy of H16882     Constrained at     0.5766 Check
PLAT300_ALERT_4_G Atom Site Occupancy of H16883     Constrained at     0.5766 Check
PLAT300_ALERT_4_G Atom Site Occupancy of H6721      Constrained at     0.2511 Check
PLAT300_ALERT_4_G Atom Site Occupancy of H6722      Constrained at     0.2511 Check
PLAT300_ALERT_4_G Atom Site Occupancy of H6723      Constrained at     0.2511 Check
PLAT300_ALERT_4_G Atom Site Occupancy of H6731      Constrained at     0.2511 Check
PLAT300_ALERT_4_G Atom Site Occupancy of H6732      Constrained at     0.2511 Check
PLAT300_ALERT_4_G Atom Site Occupancy of H6733      Constrained at     0.2511 Check
PLAT300_ALERT_4_G Atom Site Occupancy of H6741      Constrained at     0.2511 Check
PLAT300_ALERT_4_G Atom Site Occupancy of H6742      Constrained at     0.2511 Check
PLAT300_ALERT_4_G Atom Site Occupancy of H6743      Constrained at     0.2511 Check
PLAT300_ALERT_4_G Atom Site Occupancy of H6861      Constrained at     0.4234 Check
PLAT300_ALERT_4_G Atom Site Occupancy of H6862      Constrained at     0.4234 Check
PLAT300_ALERT_4_G Atom Site Occupancy of H6863      Constrained at     0.4234 Check
PLAT300_ALERT_4_G Atom Site Occupancy of H6871      Constrained at     0.4234 Check
PLAT300_ALERT_4_G Atom Site Occupancy of H6872      Constrained at     0.4234 Check
PLAT300_ALERT_4_G Atom Site Occupancy of H6873      Constrained at     0.4234 Check
PLAT300_ALERT_4_G Atom Site Occupancy of H6881      Constrained at     0.4234 Check
PLAT300_ALERT_4_G Atom Site Occupancy of H6882      Constrained at     0.4234 Check
PLAT300_ALERT_4_G Atom Site Occupancy of H6883      Constrained at     0.4234 Check
PLAT300_ALERT_4_G Atom Site Occupancy of H16901     Constrained at     0.4134 Check
PLAT300_ALERT_4_G Atom Site Occupancy of H16902     Constrained at     0.4134 Check
PLAT300_ALERT_4_G Atom Site Occupancy of H16903     Constrained at     0.4134 Check
PLAT300_ALERT_4_G Atom Site Occupancy of H16911     Constrained at     0.4134 Check
PLAT300_ALERT_4_G Atom Site Occupancy of H16912     Constrained at     0.4134 Check
PLAT300_ALERT_4_G Atom Site Occupancy of H16914     Constrained at     0.4134 Check
PLAT300_ALERT_4_G Atom Site Occupancy of H16921     Constrained at     0.4134 Check
```

```
PLAT300_ALERT_4_G Atom Site Occupancy of H16922     Constrained at     0.4134 Check
PLAT300_ALERT_4_G Atom Site Occupancy of H16923     Constrained at     0.4134 Check
PLAT300_ALERT_4_G Atom Site Occupancy of H17451     Constrained at     0.4517 Check
PLAT300_ALERT_4_G Atom Site Occupancy of H17461     Constrained at     0.4517 Check
PLAT300_ALERT_4_G Atom Site Occupancy of H17462     Constrained at     0.4517 Check
PLAT300_ALERT_4_G Atom Site Occupancy of H17463     Constrained at     0.4517 Check
PLAT300_ALERT_4_G Atom Site Occupancy of H17471     Constrained at     0.4517 Check
PLAT300_ALERT_4_G Atom Site Occupancy of H17472     Constrained at     0.4517 Check
PLAT300_ALERT_4_G Atom Site Occupancy of H17473     Constrained at     0.4517 Check
PLAT300_ALERT_4_G Atom Site Occupancy of H17481     Constrained at     0.4517 Check
PLAT300_ALERT_4_G Atom Site Occupancy of H17491     Constrained at     0.4517 Check
PLAT300_ALERT_4_G Atom Site Occupancy of H17492     Constrained at     0.4517 Check
PLAT300_ALERT_4_G Atom Site Occupancy of H17493     Constrained at     0.4517 Check
PLAT300_ALERT_4_G Atom Site Occupancy of H17501     Constrained at     0.4517 Check
PLAT300_ALERT_4_G Atom Site Occupancy of H17502     Constrained at     0.4517 Check
PLAT300_ALERT_4_G Atom Site Occupancy of H17503     Constrained at     0.4517 Check
PLAT300_ALERT_4_G Atom Site Occupancy of H17512     Constrained at     0.4517 Check
PLAT300_ALERT_4_G Atom Site Occupancy of H17521     Constrained at     0.4517 Check
PLAT300_ALERT_4_G Atom Site Occupancy of H17522     Constrained at     0.4517 Check
PLAT300_ALERT_4_G Atom Site Occupancy of H17523     Constrained at     0.4517 Check
PLAT300_ALERT_4_G Atom Site Occupancy of H17531     Constrained at     0.4517 Check
PLAT300_ALERT_4_G Atom Site Occupancy of H17532     Constrained at     0.4517 Check
PLAT300_ALERT_4_G Atom Site Occupancy of H17533     Constrained at     0.4517 Check
PLAT300_ALERT_4_G Atom Site Occupancy of Si144      Constrained at     0.7279 Check
PLAT300_ALERT_4_G Atom Site Occupancy of Si1144     Constrained at     0.2721 Check
PLAT300_ALERT_4_G Atom Site Occupancy of C124       Constrained at     0.5349 Check
PLAT300_ALERT_4_G Atom Site Occupancy of C125       Constrained at     0.5349 Check
PLAT300_ALERT_4_G Atom Site Occupancy of C126       Constrained at     0.5349 Check
PLAT300_ALERT_4_G Atom Site Occupancy of C127       Constrained at     0.5349 Check
PLAT300_ALERT_4_G Atom Site Occupancy of C128       Constrained at     0.5349 Check
PLAT300_ALERT_4_G Atom Site Occupancy of C129       Constrained at     0.5349 Check
PLAT300_ALERT_4_G Atom Site Occupancy of C142       Constrained at     0.7279 Check
PLAT300_ALERT_4_G Atom Site Occupancy of C143       Constrained at     0.7279 Check
PLAT300_ALERT_4_G Atom Site Occupancy of C145       Constrained at     0.7279 Check
PLAT300_ALERT_4_G Atom Site Occupancy of C146       Constrained at     0.7279 Check
PLAT300_ALERT_4_G Atom Site Occupancy of C147       Constrained at     0.7279 Check
PLAT300_ALERT_4_G Atom Site Occupancy of C148       Constrained at     0.7279 Check
PLAT300_ALERT_4_G Atom Site Occupancy of C149       Constrained at     0.7279 Check
PLAT300_ALERT_4_G Atom Site Occupancy of C150       Constrained at     0.7279 Check
PLAT300_ALERT_4_G Atom Site Occupancy of C151       Constrained at     0.7279 Check
PLAT300_ALERT_4_G Atom Site Occupancy of C152       Constrained at     0.7279 Check
PLAT300_ALERT_4_G Atom Site Occupancy of C153       Constrained at     0.7279 Check
PLAT300_ALERT_4_G Atom Site Occupancy of C1124      Constrained at     0.4651 Check
PLAT300_ALERT_4_G Atom Site Occupancy of C1125      Constrained at     0.4651 Check
PLAT300_ALERT_4_G Atom Site Occupancy of C1126      Constrained at     0.4651 Check
PLAT300_ALERT_4_G Atom Site Occupancy of C1127      Constrained at     0.4651 Check
PLAT300_ALERT_4_G Atom Site Occupancy of C1128      Constrained at     0.4651 Check
PLAT300_ALERT_4_G Atom Site Occupancy of C1129      Constrained at     0.4651 Check
PLAT300_ALERT_4_G Atom Site Occupancy of C1142      Constrained at     0.2721 Check
PLAT300_ALERT_4_G Atom Site Occupancy of C1143      Constrained at     0.2721 Check
PLAT300_ALERT_4_G Atom Site Occupancy of C1145      Constrained at     0.2721 Check
PLAT300_ALERT_4_G Atom Site Occupancy of C1146      Constrained at     0.2721 Check
PLAT300_ALERT_4_G Atom Site Occupancy of C1147      Constrained at     0.2721 Check
PLAT300_ALERT_4_G Atom Site Occupancy of C1148      Constrained at     0.2721 Check
PLAT300_ALERT_4_G Atom Site Occupancy of C1149      Constrained at     0.2721 Check
PLAT300_ALERT_4_G Atom Site Occupancy of C1150      Constrained at     0.2721 Check
PLAT300_ALERT_4_G Atom Site Occupancy of C1151      Constrained at     0.2721 Check
```

```
PLAT300_ALERT_4_G Atom Site Occupancy of C1152      Constrained at     0.2721 Check
PLAT300_ALERT_4_G Atom Site Occupancy of C1153      Constrained at     0.2721 Check
PLAT300_ALERT_4_G Atom Site Occupancy of H1251      Constrained at     0.5349 Check
PLAT300_ALERT_4_G Atom Site Occupancy of H1261      Constrained at     0.5349 Check
PLAT300_ALERT_4_G Atom Site Occupancy of H1271      Constrained at     0.5349 Check
PLAT300_ALERT_4_G Atom Site Occupancy of H1281      Constrained at     0.5349 Check
PLAT300_ALERT_4_G Atom Site Occupancy of H1291      Constrained at     0.5349 Check
PLAT300_ALERT_4_G Atom Site Occupancy of H1451      Constrained at     0.7279 Check
PLAT300_ALERT_4_G Atom Site Occupancy of H1461      Constrained at     0.7279 Check
PLAT300_ALERT_4_G Atom Site Occupancy of H1462      Constrained at     0.7279 Check
PLAT300_ALERT_4_G Atom Site Occupancy of H1463      Constrained at     0.7279 Check
PLAT300_ALERT_4_G Atom Site Occupancy of H1471      Constrained at     0.7279 Check
PLAT300_ALERT_4_G Atom Site Occupancy of H1472      Constrained at     0.7279 Check
PLAT300_ALERT_4_G Atom Site Occupancy of H1473      Constrained at     0.7279 Check
PLAT300_ALERT_4_G Atom Site Occupancy of H1481      Constrained at     0.7279 Check
PLAT300_ALERT_4_G Atom Site Occupancy of H1491      Constrained at     0.7279 Check
PLAT300_ALERT_4_G Atom Site Occupancy of H1492      Constrained at     0.7279 Check
PLAT300_ALERT_4_G Atom Site Occupancy of H1493      Constrained at     0.7279 Check
PLAT300_ALERT_4_G Atom Site Occupancy of H1501      Constrained at     0.7279 Check
PLAT300_ALERT_4_G Atom Site Occupancy of H1502      Constrained at     0.7279 Check
PLAT300_ALERT_4_G Atom Site Occupancy of H1503      Constrained at     0.7279 Check
PLAT300_ALERT_4_G Atom Site Occupancy of H1511      Constrained at     0.7279 Check
PLAT300_ALERT_4_G Atom Site Occupancy of H1521      Constrained at     0.7279 Check
PLAT300_ALERT_4_G Atom Site Occupancy of H1522      Constrained at     0.7279 Check
PLAT300_ALERT_4_G Atom Site Occupancy of H1523      Constrained at     0.7279 Check
PLAT300_ALERT_4_G Atom Site Occupancy of H1531      Constrained at     0.7279 Check
PLAT300_ALERT_4_G Atom Site Occupancy of H1532      Constrained at     0.7279 Check
PLAT300_ALERT_4_G Atom Site Occupancy of H1533      Constrained at     0.7279 Check
PLAT300_ALERT_4_G Atom Site Occupancy of H11251     Constrained at     0.4651 Check
PLAT300_ALERT_4_G Atom Site Occupancy of H11261     Constrained at     0.4651 Check
PLAT300_ALERT_4_G Atom Site Occupancy of H11271     Constrained at     0.4651 Check
PLAT300_ALERT_4_G Atom Site Occupancy of H11281     Constrained at     0.4651 Check
PLAT300_ALERT_4_G Atom Site Occupancy of H11291     Constrained at     0.4651 Check
PLAT300_ALERT_4_G Atom Site Occupancy of H11451     Constrained at     0.2721 Check
PLAT300_ALERT_4_G Atom Site Occupancy of H11461     Constrained at     0.2721 Check
PLAT300_ALERT_4_G Atom Site Occupancy of H11462     Constrained at     0.2721 Check
PLAT300_ALERT_4_G Atom Site Occupancy of H11463     Constrained at     0.2721 Check
PLAT300_ALERT_4_G Atom Site Occupancy of H11471     Constrained at     0.2721 Check
PLAT300_ALERT_4_G Atom Site Occupancy of H11472     Constrained at     0.2721 Check
PLAT300_ALERT_4_G Atom Site Occupancy of H11473     Constrained at     0.2721 Check
PLAT300_ALERT_4_G Atom Site Occupancy of H11481     Constrained at     0.2721 Check
PLAT300_ALERT_4_G Atom Site Occupancy of H11492     Constrained at     0.2721 Check
PLAT300_ALERT_4_G Atom Site Occupancy of H11493     Constrained at     0.2721 Check
PLAT300_ALERT_4_G Atom Site Occupancy of H11494     Constrained at     0.2721 Check
PLAT300_ALERT_4_G Atom Site Occupancy of H11501     Constrained at     0.2721 Check
PLAT300_ALERT_4_G Atom Site Occupancy of H11502     Constrained at     0.2721 Check
PLAT300_ALERT_4_G Atom Site Occupancy of H11503     Constrained at     0.2721 Check
PLAT300_ALERT_4_G Atom Site Occupancy of H11511     Constrained at     0.2721 Check
PLAT300_ALERT_4_G Atom Site Occupancy of H11521     Constrained at     0.2721 Check
PLAT300_ALERT_4_G Atom Site Occupancy of H11522     Constrained at     0.2721 Check
PLAT300_ALERT_4_G Atom Site Occupancy of H11523     Constrained at     0.2721 Check
PLAT300_ALERT_4_G Atom Site Occupancy of H11531     Constrained at     0.2721 Check
PLAT300_ALERT_4_G Atom Site Occupancy of H11532     Constrained at     0.2721 Check
PLAT300_ALERT_4_G Atom Site Occupancy of H11533     Constrained at     0.2721 Check
PLAT300_ALERT_4_G Atom Site Occupancy of Si1544     Constrained at      0.565 Check
PLAT300_ALERT_4_G Atom Site Occupancy of Si544      Constrained at      0.435 Check
PLAT300_ALERT_4_G Atom Site Occupancy of C1542      Constrained at      0.565 Check
```

```
PLAT300_ALERT_4_G Atom Site Occupancy of C1543      Constrained at      0.565 Check
PLAT300_ALERT_4_G Atom Site Occupancy of C1545      Constrained at      0.565 Check
PLAT300_ALERT_4_G Atom Site Occupancy of C1546      Constrained at      0.565 Check
PLAT300_ALERT_4_G Atom Site Occupancy of C1547      Constrained at      0.565 Check
PLAT300_ALERT_4_G Atom Site Occupancy of C1548      Constrained at      0.565 Check
PLAT300_ALERT_4_G Atom Site Occupancy of C1549      Constrained at      0.565 Check
PLAT300_ALERT_4_G Atom Site Occupancy of C1550      Constrained at      0.565 Check
PLAT300_ALERT_4_G Atom Site Occupancy of C1551      Constrained at      0.565 Check
PLAT300_ALERT_4_G Atom Site Occupancy of C1552      Constrained at      0.565 Check
PLAT300_ALERT_4_G Atom Site Occupancy of C1553      Constrained at      0.565 Check
PLAT300_ALERT_4_G Atom Site Occupancy of C476       Constrained at     0.5809 Check
PLAT300_ALERT_4_G Atom Site Occupancy of C477       Constrained at     0.5809 Check
PLAT300_ALERT_4_G Atom Site Occupancy of C478       Constrained at     0.5809 Check
PLAT300_ALERT_4_G Atom Site Occupancy of C486       Constrained at     0.5907 Check
PLAT300_ALERT_4_G Atom Site Occupancy of C487       Constrained at     0.5907 Check
PLAT300_ALERT_4_G Atom Site Occupancy of C488       Constrained at     0.5907 Check
PLAT300_ALERT_4_G Atom Site Occupancy of C1476      Constrained at     0.4191 Check
PLAT300_ALERT_4_G Atom Site Occupancy of C1477      Constrained at     0.4191 Check
PLAT300_ALERT_4_G Atom Site Occupancy of C1478      Constrained at     0.4191 Check
PLAT300_ALERT_4_G Atom Site Occupancy of C1486      Constrained at     0.4093 Check
PLAT300_ALERT_4_G Atom Site Occupancy of C1487      Constrained at     0.4093 Check
PLAT300_ALERT_4_G Atom Site Occupancy of C1488      Constrained at     0.4093 Check
PLAT300_ALERT_4_G Atom Site Occupancy of C542       Constrained at      0.435 Check
PLAT300_ALERT_4_G Atom Site Occupancy of C543       Constrained at      0.435 Check
PLAT300_ALERT_4_G Atom Site Occupancy of C545       Constrained at      0.435 Check
PLAT300_ALERT_4_G Atom Site Occupancy of C546       Constrained at      0.435 Check
PLAT300_ALERT_4_G Atom Site Occupancy of C547       Constrained at      0.435 Check
PLAT300_ALERT_4_G Atom Site Occupancy of C548       Constrained at      0.435 Check
PLAT300_ALERT_4_G Atom Site Occupancy of C549       Constrained at      0.435 Check
PLAT300_ALERT_4_G Atom Site Occupancy of C550       Constrained at      0.435 Check
PLAT300_ALERT_4_G Atom Site Occupancy of C551       Constrained at      0.435 Check
PLAT300_ALERT_4_G Atom Site Occupancy of C552       Constrained at      0.435 Check
PLAT300_ALERT_4_G Atom Site Occupancy of C553       Constrained at      0.435 Check
PLAT300_ALERT_4_G Atom Site Occupancy of H4761      Constrained at     0.5809 Check
PLAT300_ALERT_4_G Atom Site Occupancy of H4762      Constrained at     0.5809 Check
PLAT300_ALERT_4_G Atom Site Occupancy of H4763      Constrained at     0.5809 Check
PLAT300_ALERT_4_G Atom Site Occupancy of H4771      Constrained at     0.5809 Check
PLAT300_ALERT_4_G Atom Site Occupancy of H4772      Constrained at     0.5809 Check
PLAT300_ALERT_4_G Atom Site Occupancy of H4773      Constrained at     0.5809 Check
PLAT300_ALERT_4_G Atom Site Occupancy of H4781      Constrained at     0.5809 Check
PLAT300_ALERT_4_G Atom Site Occupancy of H4782      Constrained at     0.5809 Check
PLAT300_ALERT_4_G Atom Site Occupancy of H4783      Constrained at     0.5809 Check
PLAT300_ALERT_4_G Atom Site Occupancy of H4861      Constrained at     0.5907 Check
PLAT300_ALERT_4_G Atom Site Occupancy of H4862      Constrained at     0.5907 Check
PLAT300_ALERT_4_G Atom Site Occupancy of H4863      Constrained at     0.5907 Check
PLAT300_ALERT_4_G Atom Site Occupancy of H4871      Constrained at     0.5907 Check
PLAT300_ALERT_4_G Atom Site Occupancy of H4872      Constrained at     0.5907 Check
PLAT300_ALERT_4_G Atom Site Occupancy of H4873      Constrained at     0.5907 Check
PLAT300_ALERT_4_G Atom Site Occupancy of H4881      Constrained at     0.5907 Check
PLAT300_ALERT_4_G Atom Site Occupancy of H4882      Constrained at     0.5907 Check
PLAT300_ALERT_4_G Atom Site Occupancy of H4883      Constrained at     0.5907 Check
PLAT300_ALERT_4_G Atom Site Occupancy of H15451     Constrained at      0.565 Check
PLAT300_ALERT_4_G Atom Site Occupancy of H15461     Constrained at      0.565 Check
PLAT300_ALERT_4_G Atom Site Occupancy of H15463     Constrained at      0.565 Check
PLAT300_ALERT_4_G Atom Site Occupancy of H15464     Constrained at      0.565 Check
PLAT300_ALERT_4_G Atom Site Occupancy of H15471     Constrained at      0.565 Check
PLAT300_ALERT_4_G Atom Site Occupancy of H15472     Constrained at      0.565 Check
```

```
PLAT300_ALERT_4_G Atom Site Occupancy of H15473     Constrained at      0.565 Check
PLAT300_ALERT_4_G Atom Site Occupancy of H15481     Constrained at      0.565 Check
PLAT300_ALERT_4_G Atom Site Occupancy of H15491     Constrained at      0.565 Check
PLAT300_ALERT_4_G Atom Site Occupancy of H15492     Constrained at      0.565 Check
PLAT300_ALERT_4_G Atom Site Occupancy of H15493     Constrained at      0.565 Check
PLAT300_ALERT_4_G Atom Site Occupancy of H15501     Constrained at      0.565 Check
PLAT300_ALERT_4_G Atom Site Occupancy of H15502     Constrained at      0.565 Check
PLAT300_ALERT_4_G Atom Site Occupancy of H15503     Constrained at      0.565 Check
PLAT300_ALERT_4_G Atom Site Occupancy of H15511     Constrained at      0.565 Check
PLAT300_ALERT_4_G Atom Site Occupancy of H15521     Constrained at      0.565 Check
PLAT300_ALERT_4_G Atom Site Occupancy of H15522     Constrained at      0.565 Check
PLAT300_ALERT_4_G Atom Site Occupancy of H15523     Constrained at      0.565 Check
PLAT300_ALERT_4_G Atom Site Occupancy of H15531     Constrained at      0.565 Check
PLAT300_ALERT_4_G Atom Site Occupancy of H15532     Constrained at      0.565 Check
PLAT300_ALERT_4_G Atom Site Occupancy of H15534     Constrained at      0.565 Check
PLAT300_ALERT_4_G Atom Site Occupancy of H5451      Constrained at      0.435 Check
PLAT300_ALERT_4_G Atom Site Occupancy of H5461      Constrained at      0.435 Check
PLAT300_ALERT_4_G Atom Site Occupancy of H5462      Constrained at      0.435 Check
PLAT300_ALERT_4_G Atom Site Occupancy of H5463      Constrained at      0.435 Check
PLAT300_ALERT_4_G Atom Site Occupancy of H5471      Constrained at      0.435 Check
PLAT300_ALERT_4_G Atom Site Occupancy of H5472      Constrained at      0.435 Check
PLAT300_ALERT_4_G Atom Site Occupancy of H5473      Constrained at      0.435 Check
PLAT300_ALERT_4_G Atom Site Occupancy of H5481      Constrained at      0.435 Check
PLAT300_ALERT_4_G Atom Site Occupancy of H5491      Constrained at      0.435 Check
PLAT300_ALERT_4_G Atom Site Occupancy of H5492      Constrained at      0.435 Check
PLAT300_ALERT_4_G Atom Site Occupancy of H5493      Constrained at      0.435 Check
PLAT300_ALERT_4_G Atom Site Occupancy of H5501      Constrained at      0.435 Check
PLAT300_ALERT_4_G Atom Site Occupancy of H5502      Constrained at      0.435 Check
PLAT300_ALERT_4_G Atom Site Occupancy of H5503      Constrained at      0.435 Check
PLAT300_ALERT_4_G Atom Site Occupancy of H5511      Constrained at      0.435 Check
PLAT300_ALERT_4_G Atom Site Occupancy of H5521      Constrained at      0.435 Check
PLAT300_ALERT_4_G Atom Site Occupancy of H5522      Constrained at      0.435 Check
PLAT300_ALERT_4_G Atom Site Occupancy of H5523      Constrained at      0.435 Check
PLAT300_ALERT_4_G Atom Site Occupancy of H5531      Constrained at      0.435 Check
PLAT300_ALERT_4_G Atom Site Occupancy of H5532      Constrained at      0.435 Check
PLAT300_ALERT_4_G Atom Site Occupancy of H5533      Constrained at      0.435 Check
PLAT300_ALERT_4_G Atom Site Occupancy of H14761     Constrained at     0.4191 Check
PLAT300_ALERT_4_G Atom Site Occupancy of H14762     Constrained at     0.4191 Check
PLAT300_ALERT_4_G Atom Site Occupancy of H14764     Constrained at     0.4191 Check
PLAT300_ALERT_4_G Atom Site Occupancy of H14771     Constrained at     0.4191 Check
PLAT300_ALERT_4_G Atom Site Occupancy of H14772     Constrained at     0.4191 Check
PLAT300_ALERT_4_G Atom Site Occupancy of H14773     Constrained at     0.4191 Check
PLAT300_ALERT_4_G Atom Site Occupancy of H14781     Constrained at     0.4191 Check
PLAT300_ALERT_4_G Atom Site Occupancy of H14782     Constrained at     0.4191 Check
PLAT300_ALERT_4_G Atom Site Occupancy of H14783     Constrained at     0.4191 Check
PLAT300_ALERT_4_G Atom Site Occupancy of H14861     Constrained at     0.4093 Check
PLAT300_ALERT_4_G Atom Site Occupancy of H14862     Constrained at     0.4093 Check
PLAT300_ALERT_4_G Atom Site Occupancy of H14863     Constrained at     0.4093 Check
PLAT300_ALERT_4_G Atom Site Occupancy of H14871     Constrained at     0.4093 Check
PLAT300_ALERT_4_G Atom Site Occupancy of H14872     Constrained at     0.4093 Check
PLAT300_ALERT_4_G Atom Site Occupancy of H14873     Constrained at     0.4093 Check
PLAT300_ALERT_4_G Atom Site Occupancy of H14881     Constrained at     0.4093 Check
PLAT300_ALERT_4_G Atom Site Occupancy of H14882     Constrained at     0.4093 Check
PLAT300_ALERT_4_G Atom Site Occupancy of H14883     Constrained at     0.4093 Check
PLAT300_ALERT_4_G Atom Site Occupancy of C642       Constrained at     0.5433 Check
PLAT300_ALERT_4_G Atom Site Occupancy of C643       Constrained at     0.5433 Check
PLAT300_ALERT_4_G Atom Site Occupancy of C644       Constrained at     0.5433 Check
```

```
PLAT300_ALERT_4_G Atom Site Occupancy of C645       Constrained at     0.5433 Check
PLAT300_ALERT_4_G Atom Site Occupancy of C646       Constrained at     0.5433 Check
PLAT300_ALERT_4_G Atom Site Occupancy of C647       Constrained at     0.5433 Check
PLAT300_ALERT_4_G Atom Site Occupancy of C1642      Constrained at     0.4567 Check
PLAT300_ALERT_4_G Atom Site Occupancy of C1643      Constrained at     0.4567 Check
PLAT300_ALERT_4_G Atom Site Occupancy of C1644      Constrained at     0.4567 Check
PLAT300_ALERT_4_G Atom Site Occupancy of C1645      Constrained at     0.4567 Check
PLAT300_ALERT_4_G Atom Site Occupancy of C1646      Constrained at     0.4567 Check
PLAT300_ALERT_4_G Atom Site Occupancy of C1647      Constrained at     0.4567 Check
PLAT300_ALERT_4_G Atom Site Occupancy of H6421      Constrained at     0.5433 Check
PLAT300_ALERT_4_G Atom Site Occupancy of H6422      Constrained at     0.5433 Check
PLAT300_ALERT_4_G Atom Site Occupancy of H6431      Constrained at     0.5433 Check
PLAT300_ALERT_4_G Atom Site Occupancy of H6432      Constrained at     0.5433 Check
PLAT300_ALERT_4_G Atom Site Occupancy of H6441      Constrained at     0.5433 Check
PLAT300_ALERT_4_G Atom Site Occupancy of H6442      Constrained at     0.5433 Check
PLAT300_ALERT_4_G Atom Site Occupancy of H6451      Constrained at     0.5433 Check
PLAT300_ALERT_4_G Atom Site Occupancy of H6452      Constrained at     0.5433 Check
PLAT300_ALERT_4_G Atom Site Occupancy of H6461      Constrained at     0.5433 Check
PLAT300_ALERT_4_G Atom Site Occupancy of H6462      Constrained at     0.5433 Check
PLAT300_ALERT_4_G Atom Site Occupancy of H6471      Constrained at     0.5433 Check
PLAT300_ALERT_4_G Atom Site Occupancy of H6472      Constrained at     0.5433 Check
PLAT300_ALERT_4_G Atom Site Occupancy of H16421     Constrained at     0.4567 Check
PLAT300_ALERT_4_G Atom Site Occupancy of H16422     Constrained at     0.4567 Check
PLAT300_ALERT_4_G Atom Site Occupancy of H16431     Constrained at     0.4567 Check
PLAT300_ALERT_4_G Atom Site Occupancy of H16432     Constrained at     0.4567 Check
PLAT300_ALERT_4_G Atom Site Occupancy of H16441     Constrained at     0.4567 Check
PLAT300_ALERT_4_G Atom Site Occupancy of H16442     Constrained at     0.4567 Check
PLAT300_ALERT_4_G Atom Site Occupancy of H16451     Constrained at     0.4567 Check
PLAT300_ALERT_4_G Atom Site Occupancy of H16452     Constrained at     0.4567 Check
PLAT300_ALERT_4_G Atom Site Occupancy of H16461     Constrained at     0.4567 Check
PLAT300_ALERT_4_G Atom Site Occupancy of H16462     Constrained at     0.4567 Check
PLAT300_ALERT_4_G Atom Site Occupancy of H16471     Constrained at     0.4567 Check
PLAT300_ALERT_4_G Atom Site Occupancy of H16472     Constrained at     0.4567 Check
PLAT300_ALERT_4_G Atom Site Occupancy of O407       Constrained at     0.6337 Check
PLAT300_ALERT_4_G Atom Site Occupancy of O414       Constrained at     0.6337 Check
PLAT300_ALERT_4_G Atom Site Occupancy of O441       Constrained at     0.6337 Check
PLAT300_ALERT_4_G Atom Site Occupancy of O448       Constrained at     0.6337 Check
PLAT300_ALERT_4_G Atom Site Occupancy of N422       Constrained at     0.6337 Check
PLAT300_ALERT_4_G Atom Site Occupancy of N425       Constrained at     0.6337 Check
PLAT300_ALERT_4_G Atom Site Occupancy of C401       Constrained at     0.6337 Check
PLAT300_ALERT_4_G Atom Site Occupancy of C402       Constrained at     0.6337 Check
PLAT300_ALERT_4_G Atom Site Occupancy of C403       Constrained at     0.6337 Check
PLAT300_ALERT_4_G Atom Site Occupancy of C404       Constrained at     0.6337 Check
PLAT300_ALERT_4_G Atom Site Occupancy of C405       Constrained at     0.6337 Check
PLAT300_ALERT_4_G Atom Site Occupancy of C406       Constrained at     0.6337 Check
PLAT300_ALERT_4_G Atom Site Occupancy of C408       Constrained at     0.6337 Check
PLAT300_ALERT_4_G Atom Site Occupancy of C409       Constrained at     0.6337 Check
PLAT300_ALERT_4_G Atom Site Occupancy of C410       Constrained at     0.6337 Check
PLAT300_ALERT_4_G Atom Site Occupancy of C411       Constrained at     0.6337 Check
PLAT300_ALERT_4_G Atom Site Occupancy of C412       Constrained at     0.6337 Check
PLAT300_ALERT_4_G Atom Site Occupancy of C413       Constrained at     0.6337 Check
PLAT300_ALERT_4_G Atom Site Occupancy of C415       Constrained at     0.6337 Check
PLAT300_ALERT_4_G Atom Site Occupancy of C416       Constrained at     0.6337 Check
PLAT300_ALERT_4_G Atom Site Occupancy of C417       Constrained at     0.6337 Check
PLAT300_ALERT_4_G Atom Site Occupancy of C418       Constrained at     0.6337 Check
PLAT300_ALERT_4_G Atom Site Occupancy of C419       Constrained at     0.6337 Check
PLAT300_ALERT_4_G Atom Site Occupancy of C420       Constrained at     0.6337 Check
```

```
PLAT300_ALERT_4_G Atom Site Occupancy of C421       Constrained at     0.6337 Check
PLAT300_ALERT_4_G Atom Site Occupancy of C423       Constrained at     0.6337 Check
PLAT300_ALERT_4_G Atom Site Occupancy of C424       Constrained at     0.6337 Check
PLAT300_ALERT_4_G Atom Site Occupancy of C426       Constrained at     0.6337 Check
PLAT300_ALERT_4_G Atom Site Occupancy of C427       Constrained at     0.6337 Check
PLAT300_ALERT_4_G Atom Site Occupancy of C428       Constrained at     0.6337 Check
PLAT300_ALERT_4_G Atom Site Occupancy of C429       Constrained at     0.6337 Check
PLAT300_ALERT_4_G Atom Site Occupancy of C430       Constrained at     0.6337 Check
PLAT300_ALERT_4_G Atom Site Occupancy of C431       Constrained at     0.6337 Check
PLAT300_ALERT_4_G Atom Site Occupancy of C432       Constrained at     0.6337 Check
PLAT300_ALERT_4_G Atom Site Occupancy of C433       Constrained at     0.6337 Check
PLAT300_ALERT_4_G Atom Site Occupancy of C434       Constrained at     0.6337 Check
PLAT300_ALERT_4_G Atom Site Occupancy of C435       Constrained at     0.6337 Check
PLAT300_ALERT_4_G Atom Site Occupancy of C436       Constrained at     0.6337 Check
PLAT300_ALERT_4_G Atom Site Occupancy of C437       Constrained at     0.6337 Check
PLAT300_ALERT_4_G Atom Site Occupancy of C438       Constrained at     0.6337 Check
PLAT300_ALERT_4_G Atom Site Occupancy of C439       Constrained at     0.6337 Check
PLAT300_ALERT_4_G Atom Site Occupancy of C440       Constrained at     0.6337 Check
PLAT300_ALERT_4_G Atom Site Occupancy of C442       Constrained at     0.6337 Check
PLAT300_ALERT_4_G Atom Site Occupancy of C443       Constrained at     0.6337 Check
PLAT300_ALERT_4_G Atom Site Occupancy of C444       Constrained at     0.6337 Check
PLAT300_ALERT_4_G Atom Site Occupancy of C445       Constrained at     0.6337 Check
PLAT300_ALERT_4_G Atom Site Occupancy of C446       Constrained at     0.6337 Check
PLAT300_ALERT_4_G Atom Site Occupancy of C447       Constrained at     0.6337 Check
PLAT300_ALERT_4_G Atom Site Occupancy of H4011      Constrained at     0.6337 Check
PLAT300_ALERT_4_G Atom Site Occupancy of H4031      Constrained at     0.6337 Check
PLAT300_ALERT_4_G Atom Site Occupancy of H4041      Constrained at     0.6337 Check
PLAT300_ALERT_4_G Atom Site Occupancy of H4051      Constrained at     0.6337 Check
PLAT300_ALERT_4_G Atom Site Occupancy of H4081      Constrained at     0.6337 Check
PLAT300_ALERT_4_G Atom Site Occupancy of H4082      Constrained at     0.6337 Check
PLAT300_ALERT_4_G Atom Site Occupancy of H4091      Constrained at     0.6337 Check
PLAT300_ALERT_4_G Atom Site Occupancy of H4092      Constrained at     0.6337 Check
PLAT300_ALERT_4_G Atom Site Occupancy of H4101      Constrained at     0.6337 Check
PLAT300_ALERT_4_G Atom Site Occupancy of H4102      Constrained at     0.6337 Check
PLAT300_ALERT_4_G Atom Site Occupancy of H4111      Constrained at     0.6337 Check
PLAT300_ALERT_4_G Atom Site Occupancy of H4112      Constrained at     0.6337 Check
PLAT300_ALERT_4_G Atom Site Occupancy of H4121      Constrained at     0.6337 Check
PLAT300_ALERT_4_G Atom Site Occupancy of H4122      Constrained at     0.6337 Check
PLAT300_ALERT_4_G Atom Site Occupancy of H4131      Constrained at     0.6337 Check
PLAT300_ALERT_4_G Atom Site Occupancy of H4132      Constrained at     0.6337 Check
PLAT300_ALERT_4_G Atom Site Occupancy of H4161      Constrained at     0.6337 Check
PLAT300_ALERT_4_G Atom Site Occupancy of H4171      Constrained at     0.6337 Check
PLAT300_ALERT_4_G Atom Site Occupancy of H4191      Constrained at     0.6337 Check
PLAT300_ALERT_4_G Atom Site Occupancy of H4201      Constrained at     0.6337 Check
PLAT300_ALERT_4_G Atom Site Occupancy of H4271      Constrained at     0.6337 Check
PLAT300_ALERT_4_G Atom Site Occupancy of H4281      Constrained at     0.6337 Check
PLAT300_ALERT_4_G Atom Site Occupancy of H4301      Constrained at     0.6337 Check
PLAT300_ALERT_4_G Atom Site Occupancy of H4311      Constrained at     0.6337 Check
PLAT300_ALERT_4_G Atom Site Occupancy of H4331      Constrained at     0.6337 Check
PLAT300_ALERT_4_G Atom Site Occupancy of H4341      Constrained at     0.6337 Check
PLAT300_ALERT_4_G Atom Site Occupancy of H4361      Constrained at     0.6337 Check
PLAT300_ALERT_4_G Atom Site Occupancy of H4371      Constrained at     0.6337 Check
PLAT300_ALERT_4_G Atom Site Occupancy of H4391      Constrained at     0.6337 Check
PLAT300_ALERT_4_G Atom Site Occupancy of H4401      Constrained at     0.6337 Check
PLAT300_ALERT_4_G Atom Site Occupancy of H4421      Constrained at     0.6337 Check
PLAT300_ALERT_4_G Atom Site Occupancy of H4422      Constrained at     0.6337 Check
PLAT300_ALERT_4_G Atom Site Occupancy of H4431      Constrained at     0.6337 Check
```

```
PLAT300_ALERT_4_G Atom Site Occupancy of H4432      Constrained at     0.6337 Check
PLAT300_ALERT_4_G Atom Site Occupancy of H4441      Constrained at     0.6337 Check
PLAT300_ALERT_4_G Atom Site Occupancy of H4442      Constrained at     0.6337 Check
PLAT300_ALERT_4_G Atom Site Occupancy of H4451      Constrained at     0.6337 Check
PLAT300_ALERT_4_G Atom Site Occupancy of H4452      Constrained at     0.6337 Check
PLAT300_ALERT_4_G Atom Site Occupancy of H4461      Constrained at     0.6337 Check
PLAT300_ALERT_4_G Atom Site Occupancy of H4462      Constrained at     0.6337 Check
PLAT300_ALERT_4_G Atom Site Occupancy of H4471      Constrained at     0.6337 Check
PLAT300_ALERT_4_G Atom Site Occupancy of H4472      Constrained at     0.6337 Check
PLAT300_ALERT_4_G Atom Site Occupancy of O1407      Constrained at     0.3663 Check
PLAT300_ALERT_4_G Atom Site Occupancy of O1414      Constrained at     0.3663 Check
PLAT300_ALERT_4_G Atom Site Occupancy of O1441      Constrained at     0.3663 Check
PLAT300_ALERT_4_G Atom Site Occupancy of O1448      Constrained at     0.3663 Check
PLAT300_ALERT_4_G Atom Site Occupancy of N1422      Constrained at     0.3663 Check
PLAT300_ALERT_4_G Atom Site Occupancy of N1425      Constrained at     0.3663 Check
PLAT300_ALERT_4_G Atom Site Occupancy of C1401      Constrained at     0.3663 Check
PLAT300_ALERT_4_G Atom Site Occupancy of C1402      Constrained at     0.3663 Check
PLAT300_ALERT_4_G Atom Site Occupancy of C1403      Constrained at     0.3663 Check
PLAT300_ALERT_4_G Atom Site Occupancy of C1404      Constrained at     0.3663 Check
PLAT300_ALERT_4_G Atom Site Occupancy of C1405      Constrained at     0.3663 Check
PLAT300_ALERT_4_G Atom Site Occupancy of C1406      Constrained at     0.3663 Check
PLAT300_ALERT_4_G Atom Site Occupancy of C1408      Constrained at     0.3663 Check
PLAT300_ALERT_4_G Atom Site Occupancy of C1409      Constrained at     0.3663 Check
PLAT300_ALERT_4_G Atom Site Occupancy of C1410      Constrained at     0.3663 Check
PLAT300_ALERT_4_G Atom Site Occupancy of C1411      Constrained at     0.3663 Check
PLAT300_ALERT_4_G Atom Site Occupancy of C1412      Constrained at     0.3663 Check
PLAT300_ALERT_4_G Atom Site Occupancy of C1413      Constrained at     0.3663 Check
PLAT300_ALERT_4_G Atom Site Occupancy of C1415      Constrained at     0.3663 Check
PLAT300_ALERT_4_G Atom Site Occupancy of C1416      Constrained at     0.3663 Check
PLAT300_ALERT_4_G Atom Site Occupancy of C1417      Constrained at     0.3663 Check
PLAT300_ALERT_4_G Atom Site Occupancy of C1418      Constrained at     0.3663 Check
PLAT300_ALERT_4_G Atom Site Occupancy of C1419      Constrained at     0.3663 Check
PLAT300_ALERT_4_G Atom Site Occupancy of C1420      Constrained at     0.3663 Check
PLAT300_ALERT_4_G Atom Site Occupancy of C1421      Constrained at     0.3663 Check
PLAT300_ALERT_4_G Atom Site Occupancy of C1423      Constrained at     0.3663 Check
PLAT300_ALERT_4_G Atom Site Occupancy of C1424      Constrained at     0.3663 Check
PLAT300_ALERT_4_G Atom Site Occupancy of C1426      Constrained at     0.3663 Check
PLAT300_ALERT_4_G Atom Site Occupancy of C1427      Constrained at     0.3663 Check
PLAT300_ALERT_4_G Atom Site Occupancy of C1428      Constrained at     0.3663 Check
PLAT300_ALERT_4_G Atom Site Occupancy of C1429      Constrained at     0.3663 Check
PLAT300_ALERT_4_G Atom Site Occupancy of C1430      Constrained at     0.3663 Check
PLAT300_ALERT_4_G Atom Site Occupancy of C1431      Constrained at     0.3663 Check
PLAT300_ALERT_4_G Atom Site Occupancy of C1432      Constrained at     0.3663 Check
PLAT300_ALERT_4_G Atom Site Occupancy of C1433      Constrained at     0.3663 Check
PLAT300_ALERT_4_G Atom Site Occupancy of C1434      Constrained at     0.3663 Check
PLAT300_ALERT_4_G Atom Site Occupancy of C1435      Constrained at     0.3663 Check
PLAT300_ALERT_4_G Atom Site Occupancy of C1436      Constrained at     0.3663 Check
PLAT300_ALERT_4_G Atom Site Occupancy of C1437      Constrained at     0.3663 Check
PLAT300_ALERT_4_G Atom Site Occupancy of C1438      Constrained at     0.3663 Check
PLAT300_ALERT_4_G Atom Site Occupancy of C1439      Constrained at     0.3663 Check
PLAT300_ALERT_4_G Atom Site Occupancy of C1440      Constrained at     0.3663 Check
PLAT300_ALERT_4_G Atom Site Occupancy of C1442      Constrained at     0.3663 Check
PLAT300_ALERT_4_G Atom Site Occupancy of C1443      Constrained at     0.3663 Check
PLAT300_ALERT_4_G Atom Site Occupancy of C1444      Constrained at     0.3663 Check
PLAT300_ALERT_4_G Atom Site Occupancy of C1445      Constrained at     0.3663 Check
PLAT300_ALERT_4_G Atom Site Occupancy of C1446      Constrained at     0.3663 Check
PLAT300_ALERT_4_G Atom Site Occupancy of C1447      Constrained at     0.3663 Check
```

```
PLAT300_ALERT_4_G Atom Site Occupancy of H14011     Constrained at     0.3663 Check
PLAT300_ALERT_4_G Atom Site Occupancy of H14031     Constrained at     0.3663 Check
PLAT300_ALERT_4_G Atom Site Occupancy of H14041     Constrained at     0.3663 Check
PLAT300_ALERT_4_G Atom Site Occupancy of H14051     Constrained at     0.3663 Check
PLAT300_ALERT_4_G Atom Site Occupancy of H14081     Constrained at     0.3663 Check
PLAT300_ALERT_4_G Atom Site Occupancy of H14082     Constrained at     0.3663 Check
PLAT300_ALERT_4_G Atom Site Occupancy of H14091     Constrained at     0.3663 Check
PLAT300_ALERT_4_G Atom Site Occupancy of H14092     Constrained at     0.3663 Check
PLAT300_ALERT_4_G Atom Site Occupancy of H14102     Constrained at     0.3663 Check
PLAT300_ALERT_4_G Atom Site Occupancy of H14103     Constrained at     0.3663 Check
PLAT300_ALERT_4_G Atom Site Occupancy of H14111     Constrained at     0.3663 Check
PLAT300_ALERT_4_G Atom Site Occupancy of H14112     Constrained at     0.3663 Check
PLAT300_ALERT_4_G Atom Site Occupancy of H14121     Constrained at     0.3663 Check
PLAT300_ALERT_4_G Atom Site Occupancy of H14122     Constrained at     0.3663 Check
PLAT300_ALERT_4_G Atom Site Occupancy of H14131     Constrained at     0.3663 Check
PLAT300_ALERT_4_G Atom Site Occupancy of H14132     Constrained at     0.3663 Check
PLAT300_ALERT_4_G Atom Site Occupancy of H14161     Constrained at     0.3663 Check
PLAT300_ALERT_4_G Atom Site Occupancy of H14171     Constrained at     0.3663 Check
PLAT300_ALERT_4_G Atom Site Occupancy of H14191     Constrained at     0.3663 Check
PLAT300_ALERT_4_G Atom Site Occupancy of H14201     Constrained at     0.3663 Check
PLAT300_ALERT_4_G Atom Site Occupancy of H14271     Constrained at     0.3663 Check
PLAT300_ALERT_4_G Atom Site Occupancy of H14281     Constrained at     0.3663 Check
PLAT300_ALERT_4_G Atom Site Occupancy of H14301     Constrained at     0.3663 Check
PLAT300_ALERT_4_G Atom Site Occupancy of H14311     Constrained at     0.3663 Check
PLAT300_ALERT_4_G Atom Site Occupancy of H14331     Constrained at     0.3663 Check
PLAT300_ALERT_4_G Atom Site Occupancy of H14341     Constrained at     0.3663 Check
PLAT300_ALERT_4_G Atom Site Occupancy of H14362     Constrained at     0.3663 Check
PLAT300_ALERT_4_G Atom Site Occupancy of H14371     Constrained at     0.3663 Check
PLAT300_ALERT_4_G Atom Site Occupancy of H14391     Constrained at     0.3663 Check
PLAT300_ALERT_4_G Atom Site Occupancy of H14401     Constrained at     0.3663 Check
PLAT300_ALERT_4_G Atom Site Occupancy of H14421     Constrained at     0.3663 Check
PLAT300_ALERT_4_G Atom Site Occupancy of H14422     Constrained at     0.3663 Check
PLAT300_ALERT_4_G Atom Site Occupancy of H14431     Constrained at     0.3663 Check
PLAT300_ALERT_4_G Atom Site Occupancy of H14432     Constrained at     0.3663 Check
PLAT300_ALERT_4_G Atom Site Occupancy of H14441     Constrained at     0.3663 Check
PLAT300_ALERT_4_G Atom Site Occupancy of H14442     Constrained at     0.3663 Check
PLAT300_ALERT_4_G Atom Site Occupancy of H14451     Constrained at     0.3663 Check
PLAT300_ALERT_4_G Atom Site Occupancy of H14452     Constrained at     0.3663 Check
PLAT300_ALERT_4_G Atom Site Occupancy of H14461     Constrained at     0.3663 Check
PLAT300_ALERT_4_G Atom Site Occupancy of H14462     Constrained at     0.3663 Check
PLAT300_ALERT_4_G Atom Site Occupancy of H14471     Constrained at     0.3663 Check
PLAT300_ALERT_4_G Atom Site Occupancy of H14472     Constrained at     0.3663 Check
PLAT300_ALERT_4_G Atom Site Occupancy of O803       Constrained at     0.9749 Check
PLAT300_ALERT_4_G Atom Site Occupancy of C804       Constrained at     0.9749 Check
PLAT300_ALERT_4_G Atom Site Occupancy of H8031      Constrained at     0.9749 Check
PLAT300_ALERT_4_G Atom Site Occupancy of H8041      Constrained at     0.9749 Check
PLAT300_ALERT_4_G Atom Site Occupancy of H8042      Constrained at     0.9749 Check
PLAT300_ALERT_4_G Atom Site Occupancy of H8043      Constrained at     0.9749 Check
PLAT301_ALERT_3_G Main Residue  Disorder .............(Resd  1  )        29% Note
PLAT302_ALERT_4_G Anion/Solvent/Minor-Residue Disorder (Resd  2  )        21% Note
PLAT302_ALERT_4_G Anion/Solvent/Minor-Residue Disorder (Resd  3  )        17% Note
PLAT302_ALERT_4_G Anion/Solvent/Minor-Residue Disorder (Resd  4  )        17% Note
PLAT302_ALERT_4_G Anion/Solvent/Minor-Residue Disorder (Resd  5  )        13% Note
PLAT302_ALERT_4_G Anion/Solvent/Minor-Residue Disorder (Resd  8  )       100% Note
PLAT302_ALERT_4_G Anion/Solvent/Minor-Residue Disorder (Resd  9  )       100% Note
PLAT302_ALERT_4_G Anion/Solvent/Minor-Residue Disorder (Resd 11  )       100% Note
PLAT304_ALERT_4_G Non-Integer Number of Atoms in ..... (Resd  8  )      57.03 Check
```

```
PLAT304_ALERT_4_G Non-Integer Number of Atoms in ..... (Resd  9  )      32.97 Check
PLAT304_ALERT_4_G Non-Integer Number of Atoms in ..... (Resd 11  )       5.85 Check
PLAT343_ALERT_2_G Unusual sp?      Angle Range in Main Residue for       C303 Check
PLAT343_ALERT_2_G Unusual sp?      Angle Range in Main Residue for       C703 Check
PLAT343_ALERT_2_G Unusual sp?      Angle Range in Main Residue for       C103 Check
PLAT343_ALERT_2_G Unusual sp?      Angle Range in Main Residue for       C503 Check
PLAT398_ALERT_2_G Deviating  C-O-C    Angle From 120 for O407          131.2 Degree
PLAT398_ALERT_2_G Deviating  C-O-C    Angle From 120 for O441          135.7 Degree
PLAT398_ALERT_2_G Deviating  C-O-C    Angle From 120 for O448          133.8 Degree
PLAT398_ALERT_2_G Deviating  C-O-C    Angle From 120 for O1407         107.6 Degree
PLAT398_ALERT_2_G Deviating  C-O-C    Angle From 120 for O1441         141.0 Degree
PLAT398_ALERT_2_G Deviating  C-O-C    Angle From 120 for O1448         109.5 Degree
PLAT410_ALERT_2_G Short Intra H...H Contact  H6031    ..H6471    .       2.04 Ang.
                                                       x,y,z  =      1_555 Check
PLAT410_ALERT_2_G Short Intra H...H Contact  H6031    ..H6472    .       1.85 Ang.
                                                       x,y,z  =      1_555 Check
PLAT410_ALERT_2_G Short Intra H...H Contact  H6371    ..H6422    .       1.95 Ang.
                                                       x,y,z  =      1_555 Check
PLAT410_ALERT_2_G Short Intra H...H Contact  H6371    ..H16422   .       1.91 Ang.
                                                       x,y,z  =      1_555 Check
PLAT412_ALERT_2_G Short Intra XH3 .. XHn     H2521    ..H2592    .       1.89 Ang.
                                                       x,y,z  =      1_555 Check
PLAT412_ALERT_2_G Short Intra XH3 .. XHn     H2541    ..H12582   .       2.12 Ang.
                                                       x,y,z  =      1_555 Check
PLAT412_ALERT_2_G Short Intra XH3 .. XHn     H6661    ..H6722    .       1.94 Ang.
                                                       x,y,z  =      1_555 Check
PLAT412_ALERT_2_G Short Intra XH3 .. XHn     H6801    ..H16883   .       2.08 Ang.
                                                       x,y,z  =      1_555 Check
PLAT412_ALERT_2_G Short Intra XH3 .. XHn     H6801    ..H6873    .       2.05 Ang.
                                                       x,y,z  =      1_555 Check
PLAT412_ALERT_2_G Short Intra XH3 .. XHn     H6821    ..H6903    .       2.10 Ang.
                                                       x,y,z  =      1_555 Check
PLAT412_ALERT_2_G Short Intra XH3 .. XHn     H6821    ..H16863   .       2.04 Ang.
                                                       x,y,z  =      1_555 Check
PLAT412_ALERT_2_G Short Intra XH3 .. XHn     H6841    ..H6923    .       2.06 Ang.
                                                       x,y,z  =      1_555 Check
PLAT412_ALERT_2_G Short Intra XH3 .. XHn     H6841    ..H16912   .       2.00 Ang.
                                                       x,y,z  =      1_555 Check
PLAT412_ALERT_2_G Short Intra XH3 .. XHn     H4701    ..H14772   .       2.12 Ang.
                                                       x,y,z  =      1_555 Check
PLAT412_ALERT_2_G Short Intra XH3 .. XHn     H4701    ..H14773   .       2.06 Ang.
                                                       x,y,z  =      1_555 Check
PLAT412_ALERT_2_G Short Intra XH3 .. XHn     H4801    ..H4883    .       2.12 Ang.
                                                       x,y,z  =      1_555 Check
PLAT412_ALERT_2_G Short Intra XH3 .. XHn     H4821    ..H14863   .       1.96 Ang.
                                                       x,y,z  =      1_555 Check
PLAT413_ALERT_2_G Short Inter XH3 .. XHn     H3211    ..H12741   .       2.14 Ang.
                                                     x,1+y,z  =      1_565 Check
PLAT413_ALERT_2_G Short Inter XH3 .. XHn     H2391    ..H11473   .       2.11 Ang.
                                                       x,y,z  =      1_555 Check
PLAT432_ALERT_2_G Short Inter X...Y Contact  C630     ..C1442            3.08 Ang.
                                                       x,y,z  =      1_555 Check
PLAT432_ALERT_2_G Short Inter X...Y Contact  C804     ..C1146            2.56 Ang.
                                                 1-x,1-y,1-z  =      2_666 Check
PLAT432_ALERT_2_G Short Inter X...Y Contact  C804     ..C1145            3.20 Ang.
                                                 1-x,1-y,1-z  =      2_666 Check
PLAT605_ALERT_4_G Largest Solvent Accessible VOID in the Structure        112 A**3
```

```
PLAT720_ALERT_4_G Number of Unusual/Non-Standard Labels ..........        867 Note
PLAT725_ALERT_2_G D-H     Calc     0.97000, Rep     0.96000 Dev...       0.01 Ang.
              C804     -H8043          1_555   1_555 ........     #   35 Check
PLAT725_ALERT_2_G D-H     Calc     0.97000, Rep     0.96000 Dev...       0.01 Ang.
              C804     -H8043          1_555   1_555 ........     #   36 Check
PLAT725_ALERT_2_G D-H     Calc     0.96000, Rep     0.97000 Dev...       0.01 Ang.
              C1428    -H14281         1_555   1_555 ........     #   64 Check
PLAT726_ALERT_2_G H...A   Calc     1.44000, Rep     1.43000 Dev...       0.01 Ang.
              H13481   -C353           1_555   1_555 ........     #   53 Check
PLAT769_ALERT_4_G CIF Embedded explicitly supplied scattering data     Please Note
PLAT809_ALERT_1_G Can not Parse the SHELXL Weighting Scheme String     Please Check
PLAT860_ALERT_3_G Number of Least-Squares Restraints ............      23577 Note
PLAT869_ALERT_4_G ALERTS Related to the Use of SQUEEZE Suppressed          ! Info
```

```
   4 ALERT level A = Most likely a serious problem - resolve or explain
   0 ALERT level B = A potentially serious problem, consider carefully
  33 ALERT level C = Check. Ensure it is not caused by an omission or oversight
 791 ALERT level G = General information/check it is not something unexpected

   7 ALERT type 1 CIF construction/syntax error, inconsistent or missing data
  66 ALERT type 2 Indicator that the structure model may be wrong or deficient
   8 ALERT type 3 Indicator that the structure quality may be low
 746 ALERT type 4 Improvement, methodology, query or suggestion
   1 ALERT type 5 Informative message, check
```

Publication of your CIF

You should attempt to resolve as many as possible of the alerts in all categories. Often the minor alerts point to easily fixed oversights, errors and omissions in your CIF or refinement strategy, so attention to these fine details can be worthwhile. In order to resolve some of the more serious problems it may be necessary to carry out additional measurements or structure refinements. However, the nature of your study may justify the reported deviations from journal submission requirements and the more serious of these should be commented upon in the discussion or experimental section of a paper or in the "special_details" fields of the CIF. *checkCIF* was carefully designed to identify outliers and unusual parameters, but every test has its limitations and alerts that are not important in a particular case may appear. Conversely, the absence of alerts does not guarantee there are no aspects of the results needing attention. It is up to the individual to critically assess their own results and, if necessary, seek expert advice.

If level A alerts remain, which you believe to be justified deviations, and you intend to submit this CIF for publication in a journal, you should additionally insert an explanation in your CIF using the Validation Reply Form (VRF) below. This will allow your explanation to be considered as part of the review process.

Validation response form

Please find below a validation response form (VRF) that can be filled in and pasted into your CIF.

```
# start Validation Reply Form
_vrf_PLAT904_I
;
PROBLEM: Number of Reflections is <  Number of Parameters       3508
RESPONSE: ...
;
# end Validation Reply Form
```

If you wish to submit your CIF for publication in Acta Crystallographica Section C or E, you should upload your CIF via the web. If you wish to submit your CIF for publication in IUCrData you should upload your CIF via the web. If your CIF is to form part of a submission to another IUCr journal, you will be asked, either during electronic submission or by the Co-editor handling your paper, to upload your CIF via our web site.

PLATON version of 13/07/2021; check.def file version of 13/07/2021

Datablock I - ellipsoid plot

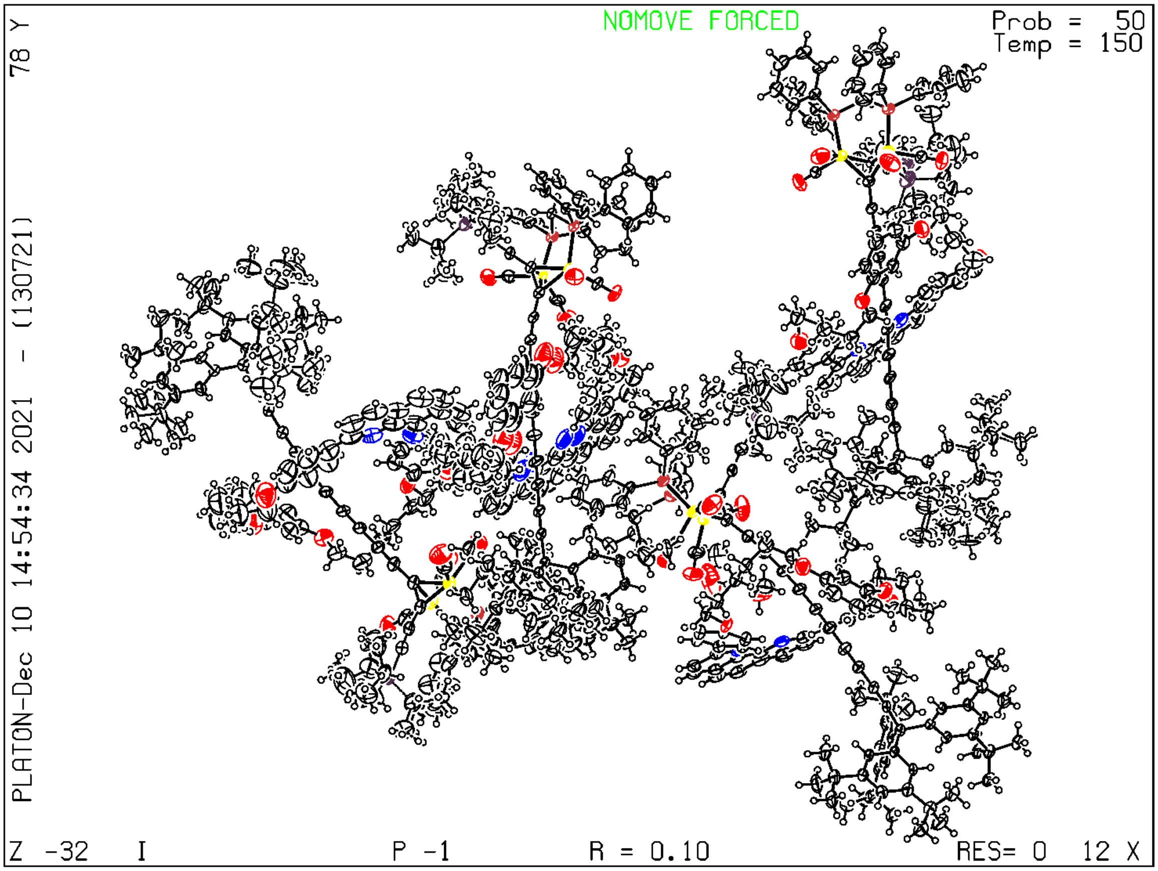

Supplement: Supplementary file 1 — Supporting Information [file ANIE-61-0-s002.pdf]
