# Supplementary material for: Polyyne [3]Rotaxanes: Synthesis via Dicobalt Carbonyl Complexes and Enhanced Stability
Source: Angew Chem Int Ed Engl. 2022 Jan 20;61(10):e202116897. doi: 10.1002/anie.202116897 (PMC9302669; doi:10.1002/anie.202116897)
Supplement: Supplementary file 3 — Supporting Information [file ANIE-61-0-s003.pdf]

Angewandte Chemie
Eine Zeitschrift der Gesellschaft Deutscher Chemiker

Supporting Information

Polyyne [3]Rotaxanes: Synthesis via Dicobalt Carbonyl Complexes and Enhanced Stability

C. W. Patrick, J. F. Woods, P. Gawel, C. E. Otteson, A. L. Thompson, T. D. W. Claridge, R. Jasti, H. L. Anderson**

Table of Contents

General Methods.....S3
Synthetic Procedures.....S4
Cobalt [2]rotaxane **4**·**M1**.....S4
Deprotected cobalt [2]rotaxane **5**·**M1**.....S4
Cobalt [3]rotaxane **6**·(**M1**)$_2$.....S5
Polyyne [3]rotaxane **1**·(**M1**)$_2$.....S6
Cobalt [2]rotaxane **4**·**M2**.....S6
Deprotected cobalt [2]rotaxane **5**·**M2**.....S7
Cobalt [3]rotaxane **6**·(**M2**)$_2$.....S7
Polyyne [3]rotaxane **1**·(**M2**)$_2$.....S8
Cobalt [2]rotaxane thread **4**.....S9
Deprotected cobalt [2]rotaxane thread **5**.....S9
Cobalt [3]rotaxane thread **6**.....S10
Supertrityl-stoppered 14-yne thread **1**.....S10
NMR Spectra.....S11
Absorption and Fluorescence Spectra.....S36
Mass Spectra.....S38
Decomposition Studies.....S41
Differential Scanning Calorimetry.....S43
References.....S44
Author Contributions.....S44

General Methods

Commercially available reagents were used as received. Dry solvents (THF, CH_2Cl_2, pentane, DMF) for reactions were purified by a MBraun MB-SPS-5 bench-top SPS system under nitrogen (H_2O content < 20 ppm). All other solvents used were HPLC grade and dried over appropriate drying agents when required. Petroleum ether (petrol) had a boiling point range of 40–60 °C. *N,N,N',N'*-Tetramethylethylenediamine (TMEDA) was dried with 3 Å molecular sieves (Linde-type) and then distilled over KOH under an Ar atmosphere prior to each use. EDTA/NH_3 solution was prepared by saturating a 1:1 solution of water/aqueous 35% NH_3 solution with tetrasodium EDTA. All solutions used during workups (NH_4Cl, $Na_2S_2O_3$ and brine) were saturated aqueous solutions, unless otherwise specified. Copper(I) chloride was freshly prepared.[1]

Reactions, unless otherwise stated, were carried out in oven-dried glassware under an Ar atmosphere. Thin layer chromatography (TLC) was carried out on aluminum-backed silica gel plates with 0.2 mm thick silica gel 60 F254 (Merck) and visualized by UV irradiation at either 254 nm or 366 nm. Preparative TLC was performed on (20 × 20 cm) Analtech 02013 Preparative Uniplates (1000 micron). Preparative flash column chromatography was either carried out using flash silica gel 60 (230-400 mesh) obtained from Sigma-Aldrich, or on a Biotage Isolera One with a 200–400 nm UV detector. Size exclusion chromatography (SEC) was carried out using Bio-Beads S-X3, 40-80 µm (Bio Rad). Evaporation of solvents was performed at 20–50 °C and 5–1010 mbar. Reported yields refer to pure compounds dried under high vacuum (< 0.1 mbar).

^{1}H and ^{13}C nuclear magnetic resonance (NMR) spectra were recorded on Bruker AVIII HD 400, Avance NEO 400, AVIII HD 500, Bruker AVIII HD 600 (Prodigy broadband cryoprobe) and Bruker AVIII 700 (with 1H/13C/15N TCI cryoprobe) spectrometers at 400 MHz, 400 MHz, 500 MHz, 600 MHz and 700 MHz (^{1}H) and 101 MHz, 126 MHz, 151 MHz and 175 MHz (^{13}C), respectively at 298 K unless stated otherwise. NMR chemical shifts were reported in ppm relative to $SiMe_4$ ($\delta = 0$) and were referenced internally with respect to residual solvent protons using the reported values. All chemical shifts are reported in ppm, coupling constants are reported in Hz and ^{1}H multiplicities are reported in accordance with the following: s = singlet; br s = broad singlet; d = doublet; t = triplet; q = quartet; and m = multiplet. ^{1}H assignments were made using 2D NMR methods (COSY, NOESY, HSQC, HMBC).

^{13}C NMR spectra of cobalt carbonyl complex of alkynes often show fewer than the expected number of peaks due to the broadness and low intensity (from the slow relaxation) of the carbons bound to cobalt and the overlap between the signals from the many carbon environments.

Electrospray mass spectrometry was carried out on a Waters Micromass LCT Premier XE spectrometer using 90:10 MeOH:H_2O (+0.1% formic acid) as the mobile phase. High-resolution mass spectrometry (HR-MS) measurements were performed either by the mass spectrometry service at the University of Oxford on a Waters GTC classic or at the NMSF at Swansea University on a Bruker ultrafleXtreme.

UV-vis spectra were recorded in solution on a Perkin-Elmer Lambda 20 or Perkin-Elmer Lambda 25 spectrometer at 25 °C (unless otherwise noted), in fused silica cuvettes with a pathlength of 1 cm. Elevated temperature UV-vis experiments were run using a heated cuvette holder connected to a Perkin Elmer PTP-1 Peltier System.

Fluorescence spectra were acquired in solution in fused silica cuvettes at 298 K using an Edinburgh Instruments FS5 spectrofluorometer operating Fluoracle® software and equipped with a xenon arc lamp (providing 230–1000 nm excitation range), a thermostatic sample holder (SC-20) and both an R13456 PMT detector (200–950 nm spectral coverage, Hamamatsu) and an InGaAs analogue NIR detector (850–1650 nm spectral coverage).

Differential scanning calorimetry (DSC) measurements were performed on a PerkinElmer DSC 4000 instrument using an aluminum plate as a reference. In cases when the sample decomposed, the onset temperature of the decomposition exothermic peak is reported, as well as the exothermic maxima corresponding to the decomposition.

The supertrityl triyne bromide stopper **3**,[2,3] TIPS-TMS triyne,[4] TMS cobalt diyne **2**,[5] phenanthroline macrocycle **M1**[6] and 2,6-pyridyl cycloparaphenylene nanohoop **M2**[7] were synthesized following reported procedures.

Synthetic Procedures

Cobalt [2]rotaxane 4·M1

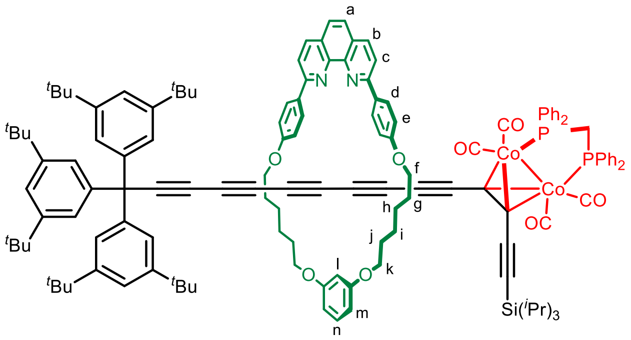

Macrocycle **M1** (212 mg, 1.05 eq., 332 µmol) and copper(I) iodide (60.3 mg, 1.00 eq., 317 µmol) were combined and dissolved in a solution of dry MeCN (10.5 mL) and dry DCM (15.0 mL), then stirred for 1 h under argon at 20 °C. The solvent was removed under reduced pressure and the residual solid dissolved in dry THF (25 mL). Cobalt diyne **2** (275 mg, 1.00 eq., 317 µmol), supertrityl triyne bromide **3** (232 mg, 1.00 eq., 317 µmol) and potassium carbonate (219 mg, 5.00 eq., 1.58 mmol) were combined and evacuated of air and flushed with argon three times. The solids were cooled in liquid nitrogen, before adding the Cu-macrocycle complex, along with additional THF (25 mL). The reaction mixture was thoroughly degassed (3 freeze-pump-thaw cycles) before warming to 60 °C and stirring under Ar for 18 h. EDTA/NH_3 solution (50 mL) was added to the cooled reaction mixture and left to stir for 1 h. Et_2O (50 mL) was added and the organic layer extracted. The aqueous layer was washed with Et_2O (2 × 20 mL) and the combined organic extracts dried over Na_2SO_4 before removing the solvent under reduced pressure. The crude product was subject to SiO_2 chromatography (pet. ether/EtOAc, gradient elution from 0 to 33%) then size-exclusion chromatography (S-X3 beads, toluene) and then recrystallized from DCM/hexane to yield [2]rotaxane **4·M1** (240 mg, 11 µmol, 35%) as a red-brown crystalline solid.

^{1}H NMR (500 MHz, $CDCl_3$) δ_H 8.49 (d, J = 8.8 Hz, 4H, H_d), 8.22 (d, J = 8.5 Hz, 2H, H_b), 8.06 (d, J = 8.4 Hz, 2H, H_c), 7.70 (s, 2H, H_a), 7.31 (d, J = 5.5 Hz, 4H, Co Ar-H), 7.25 (s, 3H, Tr* Ar-H), 7.22 – 7.16 (m, 12H, Co Ar-H), 7.15 – 7.12 (m, 4H, Co Ar-H), 7.08 (d, J = 8.2 Hz, 1H, H_n), 7.01 (t, J = 7.5 Hz, 4H, H_e), 6.86 (d, J = 1.8 Hz, 6H, Tr* Ar-H), 6.65 (t, J = 2.4 Hz, 1H, H_l), 6.49 (dd, J = 8.1, 2.3 Hz, 2H, H_m), 4.24 – 4.14 (m, 4H, H_f), 4.07 (t, J = 6.6 Hz, 4H, H_k), 3.43 – 3.26 (m, 2H, PCH_2P), 1.99 – 1.91 (m, 4H, H_g), 1.87 (q, J = 6.6 Hz, 4H, H_j), 1.64 (d, J = 5.8 Hz, 8H, $H_{h,i}$), 1.13 (s, 54H, ^{t}Bu), 1.03 (s, 21H, ^{i}Pr).

^{13}C NMR (126 MHz, $CDCl_3$) δ_C 160.82, 160.69, 156.55, 150.13, 146.18, 143.56, 136.51, 132.21 (br), 131.86, 131.37 (br), 130.28, 129.90, 129.66, 129.13, 128.55 (t, J_{C-P} = 4.9 Hz), 128.41 (t, J_{C-P} = 4.8 Hz), 127.36, 125.42, 123.78, 120.40, 118.99, 115.02, 108.38, 107.91, 101.71, 99.75, 86.66, 82.67, 80.91, 69.47, 69.40, 68.25, 67.83, 66.37, 64.68, 64.16, 63.63, 63.45, 57.46, 34.90, 31.46, 29.68, 29.28, 25.90, 25.87, 18.85, 11.55. *(7 carbons not distinguished)*

HRMS *m/z* = 2157.91431 $[M+H]^+$ ($C_{137}H_{149}Co_2N_2O_8P_2Si^+$ requires 2157.92170).

IR (ATR) (only selected signals) 2959.67 (C-H), 2924.67 (C-H), 2159.71 (s, C≡C), 2015.05 (s, C=O), 1990.65 (s, C=O) cm^{-1}.

Deprotected Cobalt [2]rotaxane 5·M1

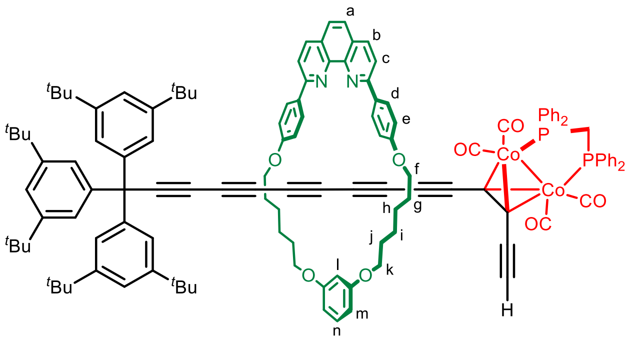

[2]Rotaxane **4·M1** (230 mg, 1.0 eq., 90 µmol) was dissolved in THF (35 mL) containing water (0.35 mL, 1% v/v). Tetrabutylammonium fluoride solution (0.53 mL, 5.0 eq., 0.53 mmol, 1.0 M in THF) was added dropwise and the mixture stirred for 30 min. The reaction was quenched with addition of aqueous NH_4Cl solution (30 mL), then Et_2O (30 mL) was added, and the organic layer extracted. The aqueous layer was washed with Et_2O (2 × 15 mL) and the combined extracts washed with brine (40 mL), then dried over Na_2SO_4. The solvent was removed under reduced pressure and the crude material purified by silica chromatography (pet. ether/EtOAc, gradient elution from 0 to 25%) to yield the deprotected [2]rotaxane **5·M1** (170 mg, 86 µmol, 81%) as a red-brown solid.

^{1}H NMR (500 MHz, $CDCl_3$) δ_H 8.45 – 8.54 (m, 4H, H_d), 8.22 (d, J = 8.4 Hz, 2H, H_b), 8.07 (d, J = 8.5 Hz, 2H, H_c), 7.71 (s, 2H, H_a), 7.23 – 7.28 (m, 4H, Co Ar–H), 7.19 – 7.21 (m, 3H, Tr* Ar–H), 7.12 – 7.24 (m, 16H, Co Ar–H), 7.09 (t, 1H, H_n), 7.04 (t, J = 7.5 Hz, 4H, H_e), 6.85 (d, J = 1.8 Hz, 6H, Tr* Ar–H), 6.65 (t, J = 2.4 Hz, 1H, H_l), 6.49 (dd, J = 8.2, 2.3 Hz, 2H, H_m), 4.15 – 4.23 (m, 4H, H_f), 4.07 (t, J = 6.6 Hz, 4H, H_k), 3.75 (s, 1H, C≡C–H), 3.29 (br. s, 2H, PCH_2P), 1.94 (q, J = 6.8 Hz, 4H, H_g), 1.86 (q, J = 6.6, 6.1 Hz, 4H, H_j), 1.60 – 1.66 (m, 8H, $H_{h,i}$), 1.12 (s, 54H, ^{t}Bu).

^{13}C NMR (126 MHz, $CDCl_3$) δ_C 201.80 (br, C≡O), 160.80, 160.67, 156.51, 150.14, 146.16, 143.53, 136.54, 135.43 (t, $J_{C\text{-}P}$ = 26.5 Hz), 133.95 (t, $J_{C\text{-}P}$ = 21.0 Hz), 131.84 (t, $J_{C\text{-}P}$ = 5.5 Hz), 131.59 (t, $J_{C\text{-}P}$ = 6.2 Hz), 130.31, 130.04, 129.64, 129.15, 128.51 (dt, $J_{C\text{-}P}$ = 5.0, 5.0 Hz), 127.37, 125.43, 123.78, 120.40, 118.98, 115.00, 107.93, 99.81, 86.75, 86.16, 85.43, 82.59, 81.03, 69.38, 69.33, 68.24, 67.89, 66.44, 64.73, 63.87, 63.48, 63.36, 57.45, 36.82 (t, J = 20.7 Hz), 34.89, 31.44, 29.69, 29.27, 25.91, 25.85. *(4 carbons not distinguished)*

^{31}P{H} NMR (202 MHz, $CDCl_3$) δ_P 39.56.

HRMS *m/z* = 2002.7985 $[M+H]^+$ ($C_{128}H_{129}Co_2N_2O_8P_2^+$ requires 2002.7992).

Cobalt [3]rotaxane 6·(M1)$_2$

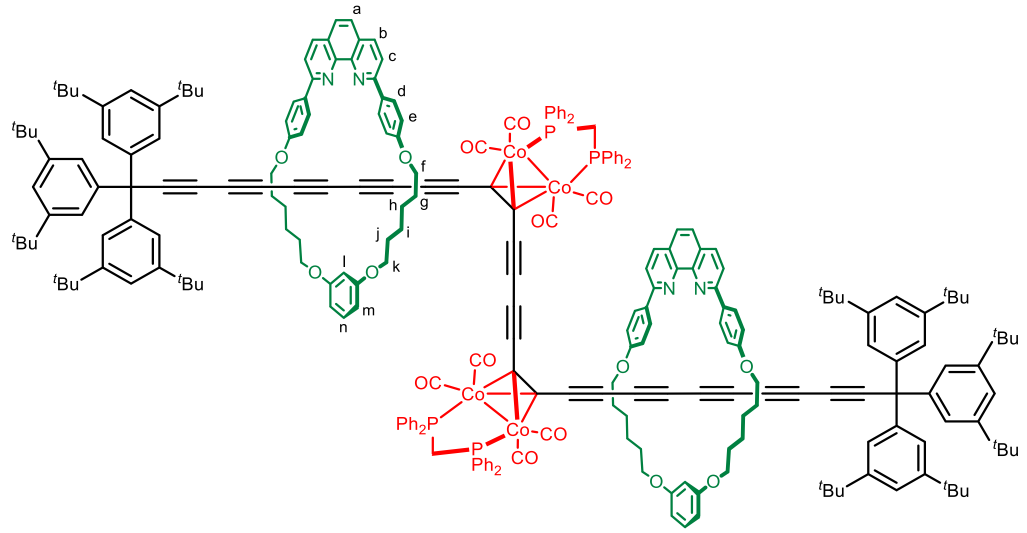

Copper(I) chloride (420 mg, 50 eq., 4.3 mmol) was added to a solution of deprotected [2]rotaxane **5·M1** (170 mg, 1.0 eq., 85 µmol) in dry DCM (150 mL). The mixture was vigorously stirred under O_2 for 5 min. Freshly-distilled TMEDA (440 mg, 570 µL, 45 eq., 3.8 mmol) was added to the solution, and was stirred vigorously under an O_2 atmosphere for 30 min. Aqueous EDTA/NH_3 solution (150 mL) was added to the mixture, and was stirred for 1 h. The organic layer was extracted, and the aqueous layer washed with DCM (2 × 50 mL). The combined extracts were washed with brine (200 mL), then dried over Na_2SO_4 before removing the solvent under reduced pressure. The crude material was purified by SiO_2 chromatography (pet. ether/EtOAc, gradient elution from 0 to 40%), then recrystallized from DCM/hexane to yield [3]rotaxane **6·**(**M1**)$_2$ (150 mg, 38 µmol, 90%) as a red-brown crystalline solid.

^{1}H NMR (500 MHz, $CDCl_3$) δ_H 8.46 (d, J = 8.6 Hz, 8H, H_d), 8.17 (d, J = 8.4 Hz, 4H, H_b), 8.00 (d, J = 8.4 Hz, 4H, H_c), 7.67 (s, 4H, H_a), 7.20 (d, J = 1.8 Hz, 6H, Tr* Ar−H), 7.19 – 7.13 (m, 28H, Co Ar−H), 7.05-7.11 (m, 20H, Co Ar−H), 7.04 (t, J = 8.1 Hz, 2H, H_n), 6.85 (d, J = 1.8 Hz, 12H, Tr* Ar−H), 6.62 (t, J = 2.4 Hz, 2H, H_l), 6.44 (dd, J = 8.2, 2.3 Hz, 4H, H_m), 4.12 – 4.18 (m, 8H, H_f), 4.03 (q, J = 6.8 Hz, 8H, H_k), 3.73 – 2.82 (m, 4H, PPh_2CH_2), 1.89 (dd, J = 12.2, 5.4 Hz, 8H, $H_{g,k}$), 1.83 (q, J = 6.9 Hz, 8H, $H_{h,i}$), 1.12 (s, 108H, ^{t}Bu).

^{13}C NMR (126 MHz, $CDCl_3$) δ_C 160.68, 160.55, 156.32, 150.02, 146.03, 143.43, 136.37, 134.15, 131.82, 131.69, 131.37, 130.18, 130.12, 129.51, 128.97, 128.61, 128.42, 127.21, 125.29, 123.66, 120.29, 118.81, 114.88, 114.82, 107.70, 99.80, 87.74, 86.65, 82.87, 77.30, 77.05, 76.80, 69.85, 69.26, 68.12, 67.73, 66.56, 64.73, 63.96, 63.49, 63.31, 57.35, 53.44, 37.79 (d, J = 21.4 Hz), 34.78, 31.33, 29.56, 29.15, 25.79. *(3 carbons not distinguished)*

HRMS *m/z* = 2001.7829 $[M+2H]^{2+}$ ($C_{256}H_{256}Co_4N_4O_{16}P_4^{2+}$ requires 2001.7838).

IR (ATR) (only selected signals) 2961.43 (C-H), 2906.40 (C-H), 2866.26 (C-H), 2360.18, 2197.60 (w, C≡C), 2153.42 (w, C≡C), 2121.36 (w, C≡C), 2016.43 (s, C=O), 1991.75 (s, C=O), 1588.55, 1487.22, 1435.23, 1249.82, 1174.11, 738.98, 321.27, 692.96 cm^{-1}.

UV-vis (DCM) λ_{max} (ε) 414 (105000), 321 (224000), 312 (121000), 290 (256000) nm.

Polyyne [3]rotaxane 1·(M1)$_2$

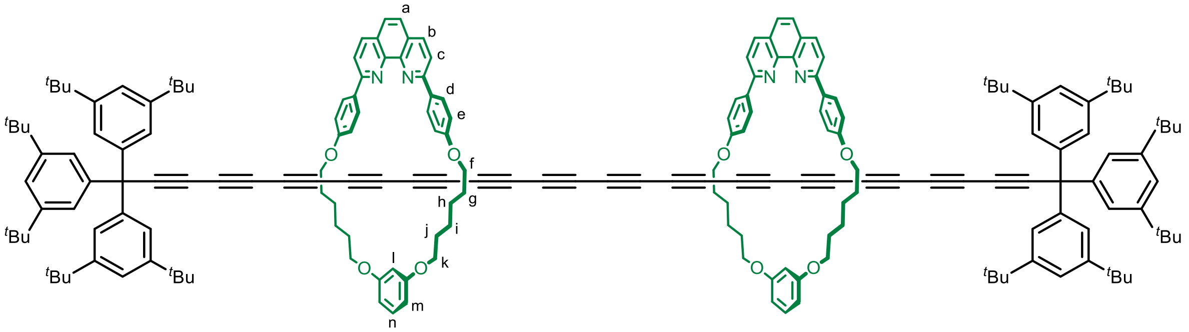

Iodine (25 mg, 20 eq., 0.10 mmol) was added to a solution of supertrityl-cobalt [3]rotaxane **6**·(**M1**)$_2$ (20 mg, 1.0 eq., 5.0 µmol) in dry THF (3 mL). The reaction was monitored by TLC and stopped upon reaching completion after 3 h. Aqueous sat. sodium sulfite solution (3 mL) was added and the mixture stirred for 10 min. The organic layer was extracted, and the aqueous layer washed with DCM (2 × 3 mL), combined and dried over Na_2SO_4. The solvent was removed under reduced pressure and the crude material purified by SiO_2 chromatography (pet. ether/EtOAc, gradient elution from 0 to 15%) to yield polyyne [3]rotaxane **1**·(**M1**)$_2$ (4.9 mg, 1.8 µmol, 36%) as a light brown solid.

^{1}H NMR (400 MHz, $CDCl_3$) δ_H 8.38 (d, J = 8.8 Hz, 8H, H_d), 8.15 (d, J = 8.5 Hz, 4H, H_b), 7.98 (dd, J = 8.5, 2.5 Hz, 4H, H_c), 7.64 (s, 4H, H_a), 7.06 (t, 2H, H_n), 7.01 – 7.03 (m, 8H, H_e), 7.00 (s, 6H, Tr* Ar–H), 6.86 (d, J = 1.8 Hz, 12H, Tr* Ar–H), 6.42 (d, J = 2.2 Hz, 2H, H_l), 6.40 (s, 4H, H_m), 3.98 (t, J = 7.4 Hz, 8H, H_f), 3.80 – 3.89 (m, 8H, H_k), 2.08 (m, 8H, H_g), 1.97 – 2.06 (m, 8H, H_j), 1.44 – 1.51 (m, 16H, $H_{h,i}$), 1.17 (d, J = 0.9 Hz, 108H, ^{t}Bu).

HRMS *m/z* = 2772.62256 $[M+H]^+$ ($C_{198}H_{210}N_4O_8^+$ requires 2772.62215).

UV-vis (hexane) λ_{max} (ε) 423 (43900), 393 (44700), 368 (37300), 348 (32100), 330 (31300), 313 (31200), 283 (43700) nm.

Cobalt [2]rotaxane 4·M2

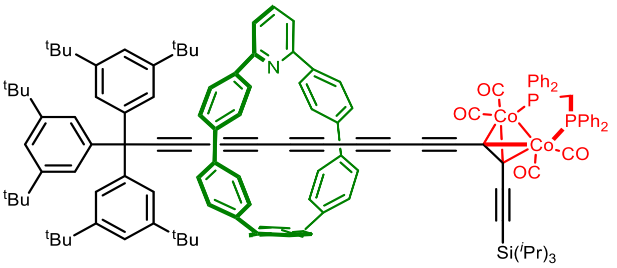

Cobalt diyne **2** (185 mg, 1.00 eq., 213 µmol), supertrityltriyne bromide **3** (156 mg, 1.00 eq., 213 µmol) macrocycle **M2** (107 mg, 1.10 eq., 234 µmol) and $[Cu(MeCN)_4][PF_6]$ (79.4 mg, 1.00 eq., 213 µmol) were combined, evacuated of air and flushed with Ar three times. The solids were cooled in liquid nitrogen, before addition of freshly-distilled diisopropylethyl amine (185 µL, 5.00 eq., 1.06 mmol) in dry $CHCl_3$ (22 mL). The reaction mixture was thoroughly degassed (3 freeze-pump-thaw cycles) before warming to 60 °C and stirred under Ar for 18 h. EDTA/NH_3 solution (22 mL) was added to the cooled reaction mixture and left to stir for 15 min. The organic layer was extracted, and the aqueous layer was washed with DCM (2 × 20 mL). The combined organic extracts were dried over Na_2SO_4 before removing the solvent under reduced pressure. The crude product was subject to SiO_2 chromatography (pet. ether/EtOAc, gradient elution from 0 to 15%) to yield the nanohoop [2]rotaxane **4·M2** (179 mg, 91.6 µmol, 43%) as a dark brown solid; R_f (Petrol/EtOAc, 9/1) = 0.40.

^{1}H NMR (500 MHz, $CDCl_3$) δ_H 7.59 (t, J = 7.7 Hz, 1H, CPP-H), 7.51 – 7.44 (m, 12H), 7.41 (dd, J = 8.8, 2.2 Hz, 2H), 7.37 – 7.31 (m, 19H), 7.26 – 7.19 (m, 8H), 7.09 (t, J = 7.5 Hz, 4H), 7.01 (d, J = 1.7 Hz, 6H, Tr* Ar-H), 3.67 – 3.25 (m, 2H, P-CH_2), 1.26 (s, 54H, ^{t}Bu), 1.14 (d, J = 5.9 Hz, 21H, Si^{i}Pr$_3$).

^{13}C NMR (126 MHz, $CDCl_3$) δ_C 205.15 (C≡O), 200.20 (C≡O), 159.89, 150.27, 143.96, 141.91, 140.81, 138.11, 137.64, 137.49, 137.29, 137.06, 132.83 (t, $J_{C\text{-}P}$ = 6.4 Hz), 131.00 (t, $J_{C\text{-}P}$ = 6.0 Hz), 130.46, 130.43, 129.91, 129.29, 129.03, 129.00, 128.77, 128.73, 128.69, 128.42, 128.38, 128.33, 128.18, 127.82, 127.18, 123.88, 120.51, 115.94, 108.60, 100.66, 85.87, 83.10, 79.28, 70.02, 69.02, 64.32, 64.25, 63.78, 62.92, 57.55, 35.05, 31.57, 29.85, 19.07, 11.72. *(3 carbons not distinguished)*

HRMS *m/z* = 1976.7884 $[M+H]^+$ ($C_{130}H_{129}Co_2NO_4P_2Si^+$ requires 1976.7903).

IR (ATR) (only selected signals) 2980.12 (C-H), 2966.07 (C-H), 2161.82 (w, C≡C), 2012.46 (s, C=O), 1987.49 (s, C=O) cm^{-1}.

UV-vis (DCM) λ_{max} (ε) 372 (81400), 332 (109000), 312 (121000), 297 (121000) nm.

Deprotected cobalt [2]rotaxane 5·M2

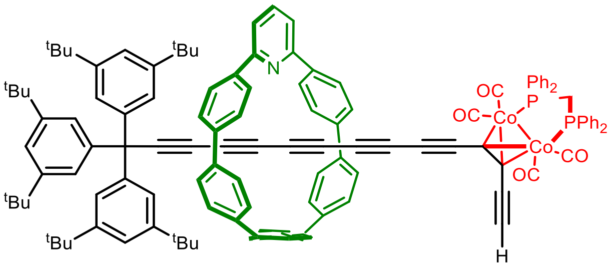

Nanohoop rotaxane **4·M2** (0.25 g, 1.0 eq., 0.13 mmol) was dissolved in THF (35 mL) containing water (0.35 mL, 1% v/v). TBAF (0.63 mL, 5.0 eq., 0.63 mmol, 1.0 M in THF) was added dropwise and the mixture stirred for 30 min. The reaction was quenched with addition of aqueous sat. NH_4Cl solution (20 mL), then pet. ether (10 mL) was added and the organic layer extracted. The aqueous layer was washed with pet. ether (2 × 10 mL) and the combined extracts washed with brine (20 mL), then dried over Na_2SO_4. The solvent was removed under reduced pressure and the crude material purified by silica chromatography (pet. ether/EtOAc, gradient elution from 15 to 20%) to give the deprotected [2] rotaxane **5·M2** (0.20 g, 0.11 mmol, 87%) as a dark brown solid; R_f (Petrol/EtOAc, 9/1) = 0.33.

^{1}H NMR (500 MHz, $CDCl_3$) δ_C 7.53 (t, J = 7.7 Hz, 1H, CPP-H), 7.44 – 7.36 (m, 12H), 7.33 (dd, J = 8.8, 2.1 Hz, 2H), 7.30 – 7.27 (m, 13H), 7.26 – 7.24 (m, 8H), 7.23 (d, J = 1.8 Hz, 2H), 7.20 (d, J = 2.0 Hz, 2H), 7.17 – 7.13 (m, 2H), 7.11 (t, J = 7.6 Hz, 4H), 6.95 (d, J = 1.8 Hz, 6H, Tr* Ar−H), 3.83 (s, 1H, C≡C–H), 3.59 (q, J = 10.5 Hz, 1H, P-CH_2), 3.20 (q, J = 10.5 Hz, 1H, P-CH_2), 1.19 (s, 54H, ^{t}Bu).

^{13}C NMR (126 MHz, $CDCl_3$) δ_C 159.90, 150.29, 143.97, 141.93, 140.81, 138.11, 137.62, 137.11, 137.07, 132.42 (t, $J_{C\text{-}P}$ = 6.2 Hz), 131.99 (t, $J_{C\text{-}P}$ = 6.3 Hz), 131.78 (t, $J_{C\text{-}P}$ = 6.3 Hz), 131.36 (t, $J_{C\text{-}P}$ = 6.2 Hz), 130.45, 130.38, 130.17, 130.09, 130.01, 128.90, 128.84, 128.79, 128.71, 128.67, 128.62, 128.57, 128.53, 128.39, 128.05, 127.67, 123.88, 120.53, 116.01, 85.78, 85.70, 85.66, 83.10, 79.16, 72.93, 70.05, 69.26, 64.31, 63.50, 62.96, 62.06, 57.57, 37.20, 35.06, 31.58. *(2 carbons not distinguished)*

^{31}P{H} NMR (202 MHz, $CDCl_3$) δ_P 39.09.

HRMS *m/z* = 1820.6546 [M+H]$^+$ ($C_{122}H_{109}Co_2NO_4P_2^+$ requires 1820.6569).

IR (ATR) (only selected signals) 2961.80 (C-H), 2904.91 (C-H), 2866.56 (C-H), 2196.23 (w, C≡C), 2160.27 (w, C≡C), 2011.48 (s, C=O), 1987.10 (s, C=O), 1591.05, 1434.97, 814.30, 738.13, 722.46, 694.88 cm^{-1}.

Cobalt [3]rotaxane 6·(M2)$_2$

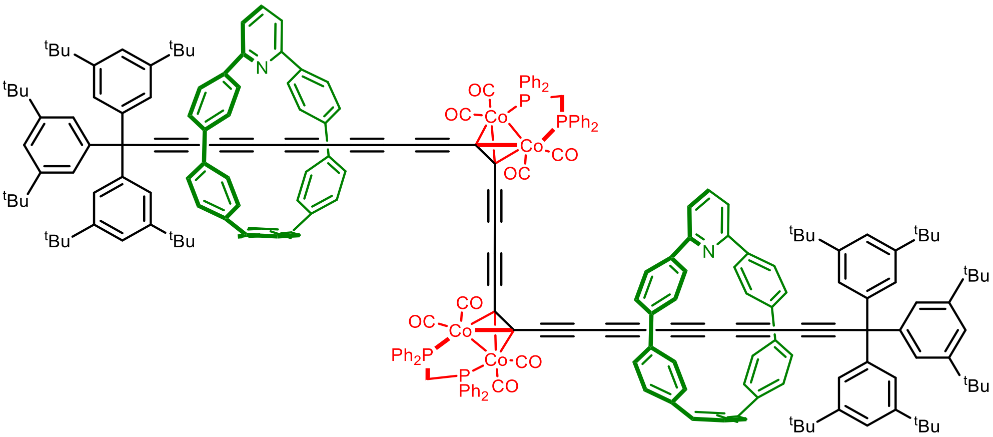

To a 100 mL round bottom flask was added deprotected nanohoop [2]rotaxane **5·M2** (125 mg, 2.00 eq., 68.6 µmol), 4,4'-di-*tert*-butyl-2,2'-bipyridine (92.1 mg, 10.0 eq., 343 µmol) and copper(I) chloride (40.8 mg, 12.0 eq., 412 µmol). Dry DCM (20 mL) was sparged with O_2 for 10 min before addition to the solids. The reaction was stirred vigorously under O_2 at 30 °C for 20 h. Upon complete reaction, the mixture was evaporated to dryness under reduced pressure. The crude material was purified first by silica chromatography (pet. ether/EtOAc, gradient elution from 10 to 25%), then SEC (S-X3, toluene) to give the nanohoop [3]rotaxane **6·(M2)$_2$** (95.7 mg, 26.3 µmol, 77%) as a brown solid.

^{1}H NMR (600 MHz, $CDCl_3$) δ_H 7.56 (t, J = 7.7 Hz, 2H, CPP-H), 7.46 – 7.40 (m, 24H), 7.38 – 7.35 (m, 8H), 7.35 – 7.27 (m, 34H), 7.24 (dt, J = 9.1, 5.1 Hz, 16H), 7.16 (t, J = 7.5 Hz, 8H), 7.02 (d, J = 1.8 Hz, 12H, Tr* Ar−H), 3.71 (q, J = 11.4 Hz, 2H, P-CH_2), 3.17 (q, J = 11.4 Hz, 2H, P-CH_2), 1.26 (s, 108H, ^{t}Bu).

^{13}C NMR (151 MHz, $CDCl_3$) δ_C 204.99 (C≡O), 200.17 (C≡O), 159.84, 150.31, 143.97, 141.91, 140.85, 138.12, 137.64, 137.08, 135.85 (t, $J_{C\text{-}P}$ = 24.0 Hz), 133.53 (t, $J_{C\text{-}P}$ = 22.0 Hz), 132.58 (t, $J_{C\text{-}P}$ = 5.9 Hz), 131.14 (t, $J_{C\text{-}P}$ = 5.9 Hz), 130.48, 130.43, 130.33, 130.01, 128.99 (t, $J_{C\text{-}P}$ = 5.0 Hz), 128.87, 128.57, 128.55 (t, $J_{C\text{-}P}$ = 5.1 Hz), 128.36, 128.03, 127.61, 123.88, 87.84, 87.79, 85.90, 85.36, 83.31, 79.77, 79.69, 70.03, 69.73 (br), 64.61, 64.29, 63.45 (br), 63.09, 62.14 (br), 57.58, 36.63 (br), 35.07, 31.59. *(6 carbons not distinguished)*

IR (ATR) (only selected signals) 2962.23 (C-H), 2905.46 (C-H), 2866.71 (C-H), 2198.35 (w, C≡C), 2161.59 (w, C≡C), 2124.39 (w, C≡C), 2032.82 (s, C=O), 2013.40 (s, C=O), 1989.14 (s, C=O), 1591.67, 1435.03, 813.91, 738.29, 722.56, 697.82 cm^{-1}.

UV-vis (DCM) λ_{max} (ε) 572 (7980), 410 (147000), 383 (137000), 318 (277000) nm.

Polyyne [3]rotaxane 1·(M2)$_2$

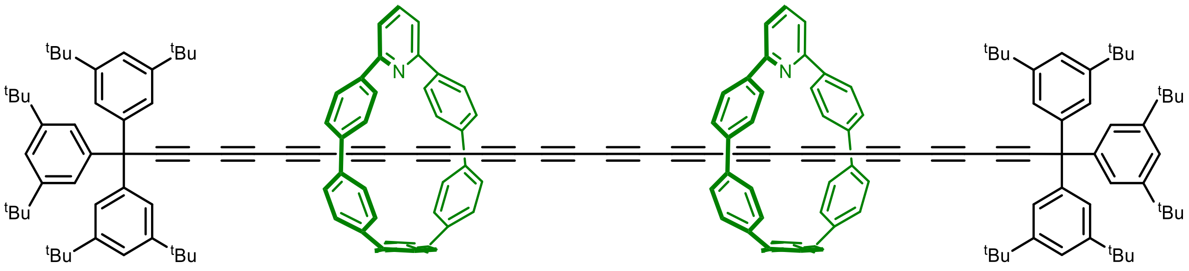

Masked nanohoop [3]rotaxane **6**·(**M2**)$_2$ (40.0 mg, 1.00 eq., 11.0 µmol) was suspended in dry MeCN (5 mL) under Ar. A solution of iodine (27.9 mg, 10.0 eq., 110 µmol) in dry THF (5 mL) was then added and the mixture stirred at 25 °C for 5 min, until complete conversion was indicated by TLC. $Na_2S_2O_3$ (4 mL) was added to quench the reaction, then the organic layer extracted, dried over Na_2SO_4 and the solvent removed under reduced pressure. The crude material purified by a silica plug (pet. ether/EtOAc, gradient elution from 0 to 15%) then preparative TLC (pet. ether/EtOAc, 6%) to give nanohoop polyyne [3]rotaxane **1**·(**M2**)$_2$ (8.6 mg, 3.6 µmol, 32%) as a yellow-orange solid; R_f (pet. ether/EtOAc, 6%) = 0.70.

^{1}H NMR (600 MHz, $CDCl_3$) δ_H 7.72 (t, J = 7.7 Hz, 2H, CPP-H), 7.47 (d, J = 7.7 Hz, 4H, CPP-H), 7.46 – 7.44 (m, 6H, CPP-H), 7.43 (d, J = 2.1 Hz, 4H, CPP-H), 7.43 – 7.42 (m, 6H, CPP-H), 7.40 (dd, J = 8.3, 1.9 Hz, 4H, CPP-H), 7.37 (dd, J = 8.8, 2.1 Hz, 4H, CPP-H), 7.35 (dd, J = 8.9, 2.2 Hz, 4H, CPP-H), 7.32 (t, J = 1.8 Hz, 6H, Tr* Ar-H), 7.30 (dd, J = 8.3, 2.0 Hz, 4H, CPP-H), 7.28 (d, J = 1.8 Hz, 2H, CPP-H), 7.28 – 7.26 (m, 4H, overlapping CPP-H), 7.25 (d, J = 2.1 Hz, 2H, CPP-H), 6.96 (d, J = 1.7 Hz, 12H, Tr* Ar-H), 1.24 (s, 108H, ^{t}Bu).

Note the asymmetry of the nanohoop results in a series of doublet of doublets (confirmed by high-resolution HSQC experiments). Assignments were made using a combination of HSQC and diffusion-edited ^{1}H experiments.

^{13}C NMR (151 MHz, $CDCl_3$) δ_C 159.79, 150.46, 143.43, 142.01, 140.73, 137.95, 137.64, 137.48, 136.94, 130.42, 129.97, 128.87, 128.76, 128.63, 128.39, 128.32, 128.25, 127.94, 127.45, 123.86, 120.71, 116.22, 87.06, 68.91, 68.32, 63.61, 63.54, 63.17, 62.88, 62.66, 62.47, 62.26, 62.06, 61.94, 61.87, 61.80, 57.56, 35.05, 31.54.

MS (MALDI-TOF, DCTB matrix) 2409.5 $[M+H]^+$ ($C_{184}H_{172}N_2$ requires 2409.4).

UV-vis (*n*-hexane) λ_{max} (ε) 425 (80700), 395 (78300), 371 (46000), 350 (24600), 331 (19700), 316 (20600), 294 (25700), 283 (24100), 272 (19700), 259 (16200), 248 (16800) nm.

IR (ATR) (only selected signals) 2961.22, 2926.82, 2866.55, 2199.69, 2129.29, 2080.81, 2013.60, 1934.64, 1591.84, 814.84, 722.72, 699.87 cm^{-1}.

Cobalt [2]rotaxane thread 4

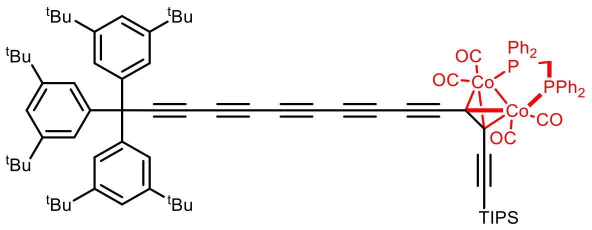

Supertrityltriyne bromide **3** (77.2 mg, 1.00 eq., 106 µmol), deprotected cobalt diyne **2** (110 mg, 1.20 eq., 127 µmol), bis(triphenylphosphine)palladium(II) dichloride (7.41 mg, 0.10 eq., 10.6 µmol) and copper(I) iodide (2.01 mg, 0.10 eq., 10.6 µmol) were cooled in liquid nitrogen before addition of dry THF (10 mL) and freshly-distilled diisopropylamine (32.0 mg, 45 µL, 3.0 eq., 317 µmol). The mixture was degassed by three freeze-pump-thaw cycles before being left to stir under Ar for 18 h at 25 °C. The reaction was quenched by addition of sat. aqueous NH_4Cl solution (10 mL). The mixture was diluted with pet. ether (10 mL), extracted then the organic layer washed with water (20 mL), then brine (20 mL). The organic layer was dried over Na_2SO_4, filtered, and concentrated under reduced pressure. The crude material was purified by silica chromatography (pet. ether/DCM, gradient elution from 0 to 33%) to give dumbbell **4** (79.4 mg, 52.2 µmol, 50%) as a red solid.

^{1}H NMR (500 MHz, $CDCl_3$) δ_H 7.39 (q, *J* = 7.5 Hz, 4H, Co Ar-H), 7.34 – 7.26 (m, 10H), 7.26 – 7.22 (m, 5H), 7.15 (t, *J* = 7.5 Hz, 4H, Co Ar-H), 6.94 (d, *J* = 1.8 Hz, 6H, Tr* Ar-H), 3.42 (t, *J* = 9.9 Hz, 2H, P-CH_2), 1.22 (s, 54H, ^{t}Bu), 1.11 (d, *J* = 4.6 Hz, 21H, ^{i}Pr$_3$).

^{13}C NMR (151 MHz, $CDCl_3$) δ_C 202.87 (br, C≡O), 201.62 (br, C≡O), 150.25, 143.69, 136.33 (br), 133.69 (br), 132.30 (t, $J_{C\text{-}P}$ = 6.4 Hz), 131.47 (t, $J_{C\text{-}P}$ = 6.2 Hz), 130.31, 130.02, 128.65 (t, $J_{C\text{-}P}$ = 4.9 Hz), 128.50 (t, $J_{C\text{-}P}$ = 4.9 Hz), 123.88, 120.48, 108.53, 101.52, 86.66, 82.45, 81.17, 69.57, 69.45, 66.15, 64.37, 63.93, 63.69, 63.61, 57.51, 36.51 (br), 35.00, 31.53, 18.92, 11.62. *(2 carbons not distinguished)*

^{31}P{H} NMR (202 MHz, $CDCl_3$) δ_P 39.03.

HRMS *m/z* = 1519.6073 [M+H]$^+$ ($C_{95}H_{107}Co_2O_4P_2Si^+$ requires 1519.6072).

Deprotected cobalt [2]rotaxane thread 5

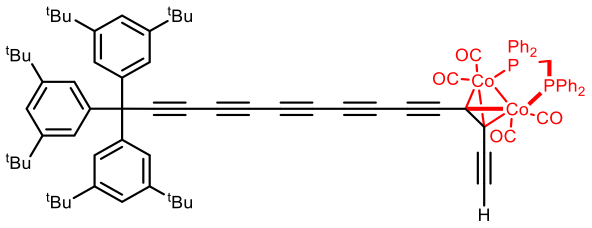

TIPS-protected [2]rotaxane thread **4** (89 mg, 1.0 eq., 59 µmol) was dissolved in THF (10 mL) containing water (0.10 mL). TBAF (0.29 mL, 1.0 M in THF, 5.0 eq., 0.29 mmol) was added dropwise and the mixture stirred for 90 min. The reaction was quenched with addition of sat. aqueous NH_4Cl solution (10 mL), then Et_2O (10 mL) was added and the organic layer extracted. The aqueous layer was washed with Et_2O (2 × 10 mL) and the combined extracts washed with brine (20 mL), then dried over Na_2SO_4. The solvent was removed under reduced pressure and the crude material purified by silica chromatography (pet. ether/EtOAc, gradient elution from 0 to 25%) to afford the deprotected [2]rotaxane thread **5** (66 mg, 48 µmol, 83%) as a red-brown solid.

^{1}H NMR (500 MHz, $CDCl_3$) δ_H 7.36 – 7.31 (m, 4H, Co Ar-H), 7.30 – 7.26 (m, 8H), 7.25 (m, 2H), 7.24 – 7.17 (m, 9H), 6.91 (d, *J* = 1.8 Hz, 6H, Tr* Ar-H), 3.78 (t, *J* = 1.7 Hz, 1H, C≡C–H), 3.38 (q, *J* = 12.7 Hz, 2H, P-CH_2), 1.19 (s, 54H, ^{t}Bu).

^{13}C NMR (126 MHz, $CDCl_3$) δ_C 201.80 (br, C≡O), 150.25, 143.65, 135.40 (br), 134.24 (br), 131.98 (t, $J_{C\text{-}P}$ = 6.2 Hz), 131.70 (t, $J_{C\text{-}P}$ = 6.2 Hz), 130.35, 130.21, 128.64 (td, $J_{C\text{-}P}$ = 4.9, 2.1 Hz), 123.87, 120.49, 86.71, 85.65, 85.62, 81.97, 81.65, 69.64, 69.37, 66.22, 64.44, 63.62, 63.54, 63.50, 57.49, 37.37, 35.00, 31.52. *(4 carbons not distinguished)*

^{31}P{H} NMR (203 MHz, $CDCl_3$) δ_P 39.71.

HRMS *m/z* = 1363.4744 [M+H]$^+$ ($C_{86}H_{87}Co_2O_4P_2^+$ requires 1363.4738).

Cobalt [3]rotaxane thread 6

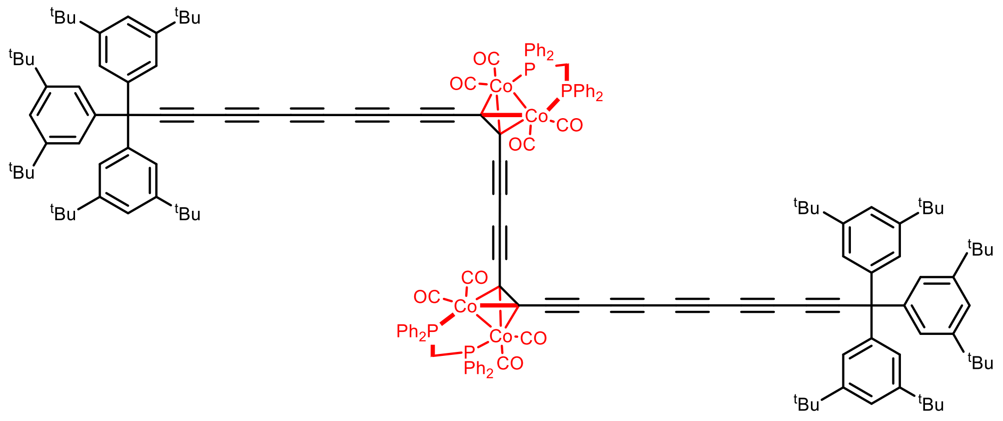

Deprotected [2]rotaxane thread **5** (65.0 mg, 2.00 eq., 47.7 µmol) and copper(I) chloride (118 mg, 50.0 eq., 1.19 mmol) were dissolved in dry DCM (20 mL). Freshly-distilled TMEDA (139 mg, 179 µL, 50.0 eq., 1.19 mmol) was added, and the mixture stirred vigorously at 25 °C for 30 min under and O_2 atmosphere. Upon complete reaction, water (20 mL) was added, and the organic layer extracted, washed with water (2 × 20 mL), then brine (20 mL). The combined organic extracts were dried over Na_2SO_4 and the solvent removed under reduced pressure. The crude material was purified by silica chromatography (pet. ether/DCM, gradient elution from 0 to 33%) to yield the masked [3]rotaxane dumbbell **6** (46.1 mg, 16.9 µmol, 71%) as a brown solid.

^{1}H NMR (600 MHz, $CDCl_3$) δ_H 7.37 – 7.26 (m, 26H), 7.26 – 7.18 (m, 20H), 6.91 (d, *J* = 1.8 Hz, 12H, Tr* Ar-H), 3.56 (s, 2H, P-CH_2), 3.34 (s, 2H, P-CH_2), 1.19 (s, 108H, ^{t}Bu).

^{13}C NMR (151 MHz, $CDCl_3$) δ_C 202.86 (br, C≡O), 201.15 (br, C≡O), 150.25, 143.67, 134.98 – 134.28 (m), 132.00 (t, *J* = 6.1 Hz), 131.69 (t, *J* = 6.0 Hz), 130.43, 130.37, 128.84 (t, *J* = 4.9 Hz), 128.69 (t, *J* = 4.9 Hz), 123.88, 120.48, 88.04, 86.77, 85.63, 82.52, 81.90, 80.26, 78.30, 70.15, 69.43, 66.48, 64.55, 63.79, 63.65, 63.59, 57.51, 38.37 (t, *J* = 21.7 Hz), 35.00, 31.53. *(1 carbon not distinguished)*

^{31}P{H} NMR (243 MHz, $CDCl_3$) δ_P 40.06.

Supertrityl-stoppered 14-yne thread 1

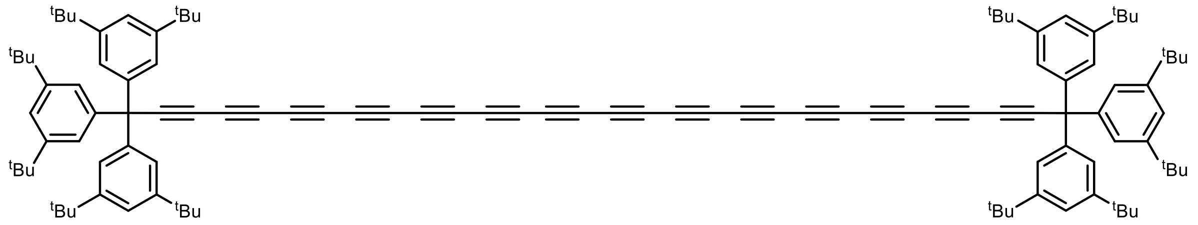

Masked [3]rotaxane thread **6** (45 mg, 1.0 eq., 17 µmol) was charged into a flask and flushed with Ar. A solution of iodine (84 mg, 20 eq., 0.33 mmol) in dry THF (20 mL) was added and the mixture was stirred at 25 °C for 5 min, until complete conversion by TLC. Sat. $Na_2S_2O_3$ (20 mL) was added, the organic layer extracted, dried over Na_2SO_4 and the solvent was removed under reduced pressure. The crude material purified by a silica plug (pet. ether/DCM, 15%) to give the supertrityl-stoppered 14-yne **1** (1.8 mg, 1.2 µmol, 7.3%).

^{1}H NMR (600 MHz, $CDCl_3$) δ_H 7.28 (t, *J* = 1.8 Hz, 6H, Tr* Ar-H), 6.89 (d, *J* = 1.8 Hz, 12H, Tr* Ar-H), 1.20 (s, 108H, ^{t}Bu).

^{13}C NMR (151 MHz, $CDCl_3$) δ_C 150.41, 143.29, 123.84, 120.67, 87.38, 68.71, 64.43, 64.15, 64.08, 63.85, 63.61, 63.39, 63.16, 62.89, 62.64, 62.60, 62.27, 62.12, 57.52, 35.01, 31.50.

MS (MALDI-TOF, DCTB matrix) 1495.2 $[M-H]^-$ ($C_{114}H_{126}$ requires 1495.0).

Analytical data as lit.[2]

NMR Spectra

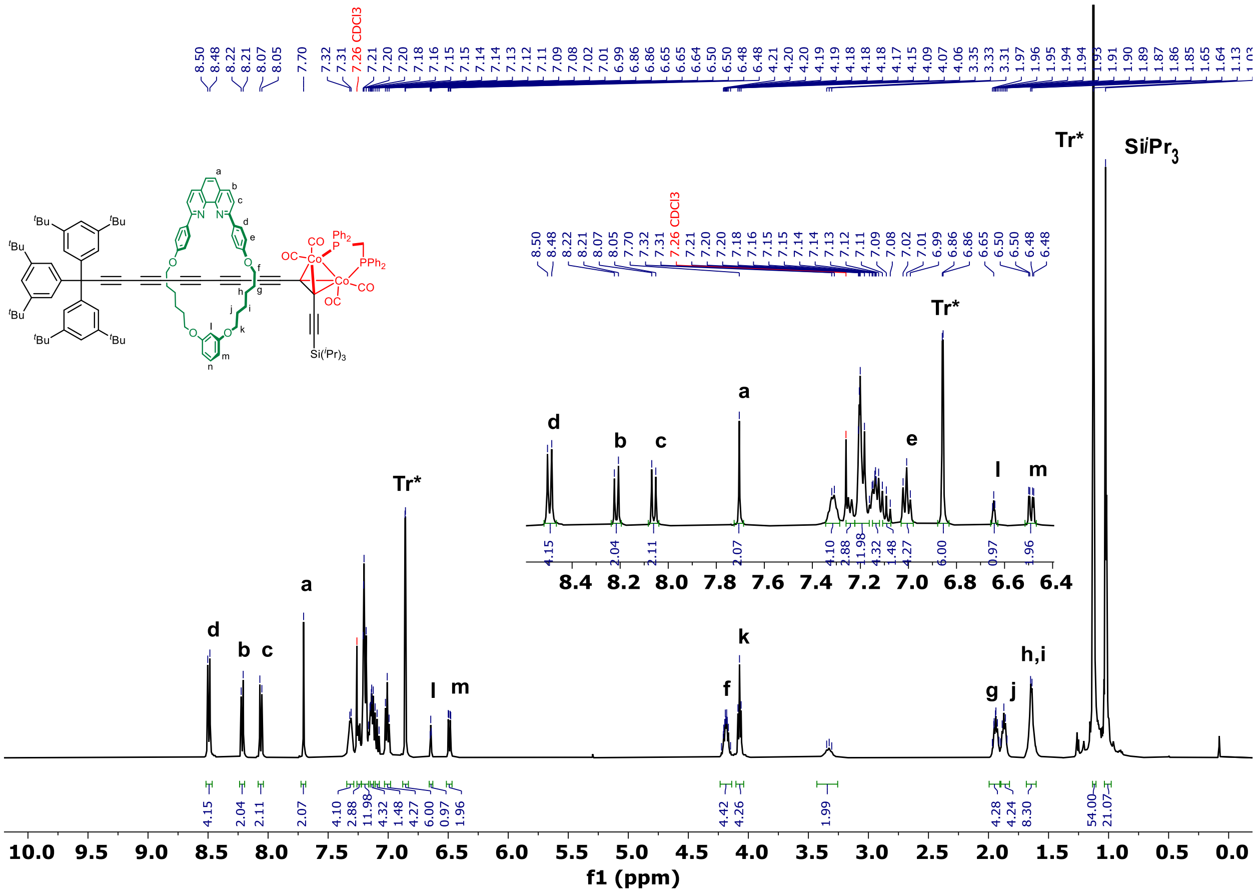

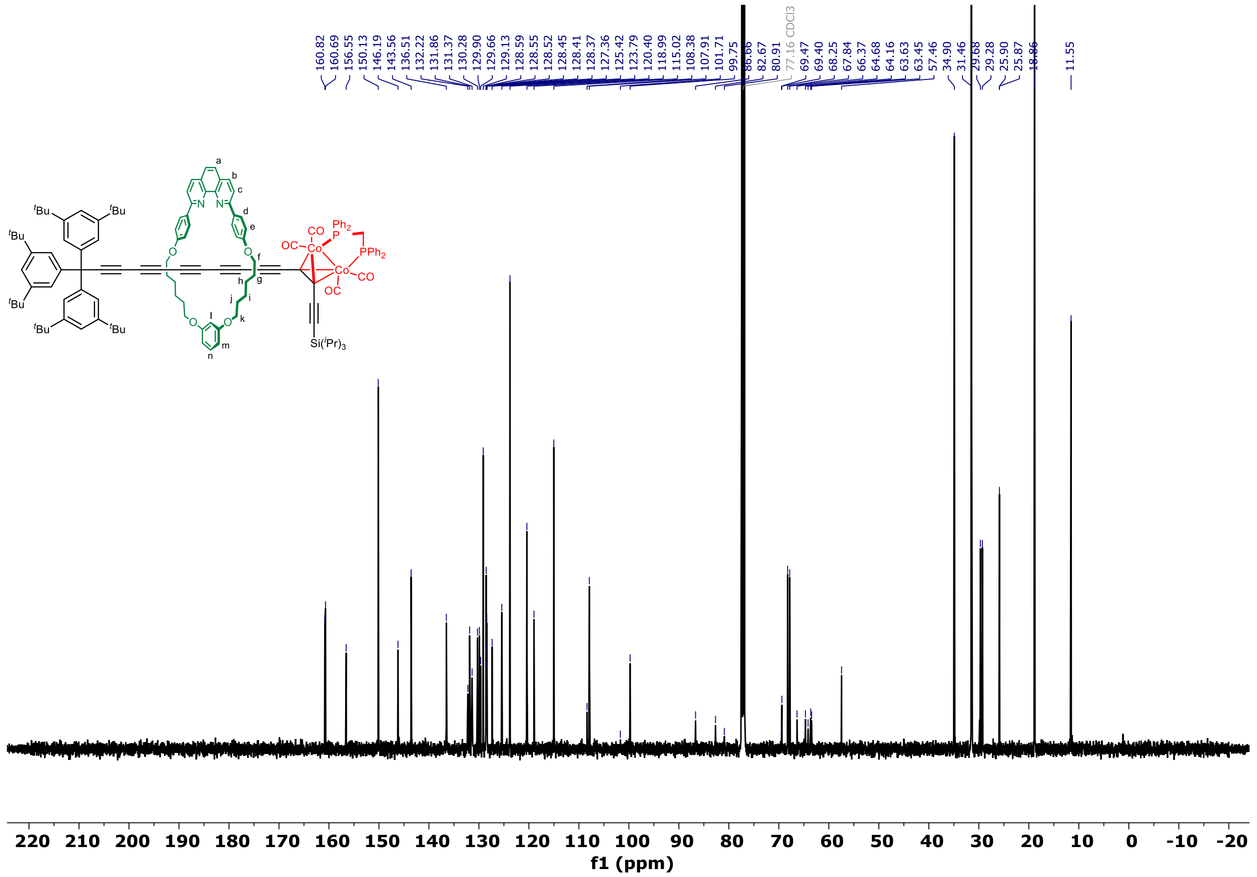

Figure S1: (top) ^{1}H NMR (500 MHz) and (bottom) ^{13}C NMR (126 MHz) spectra of [2]rotaxane **4·M1** ($CDCl_3$, 298 K).

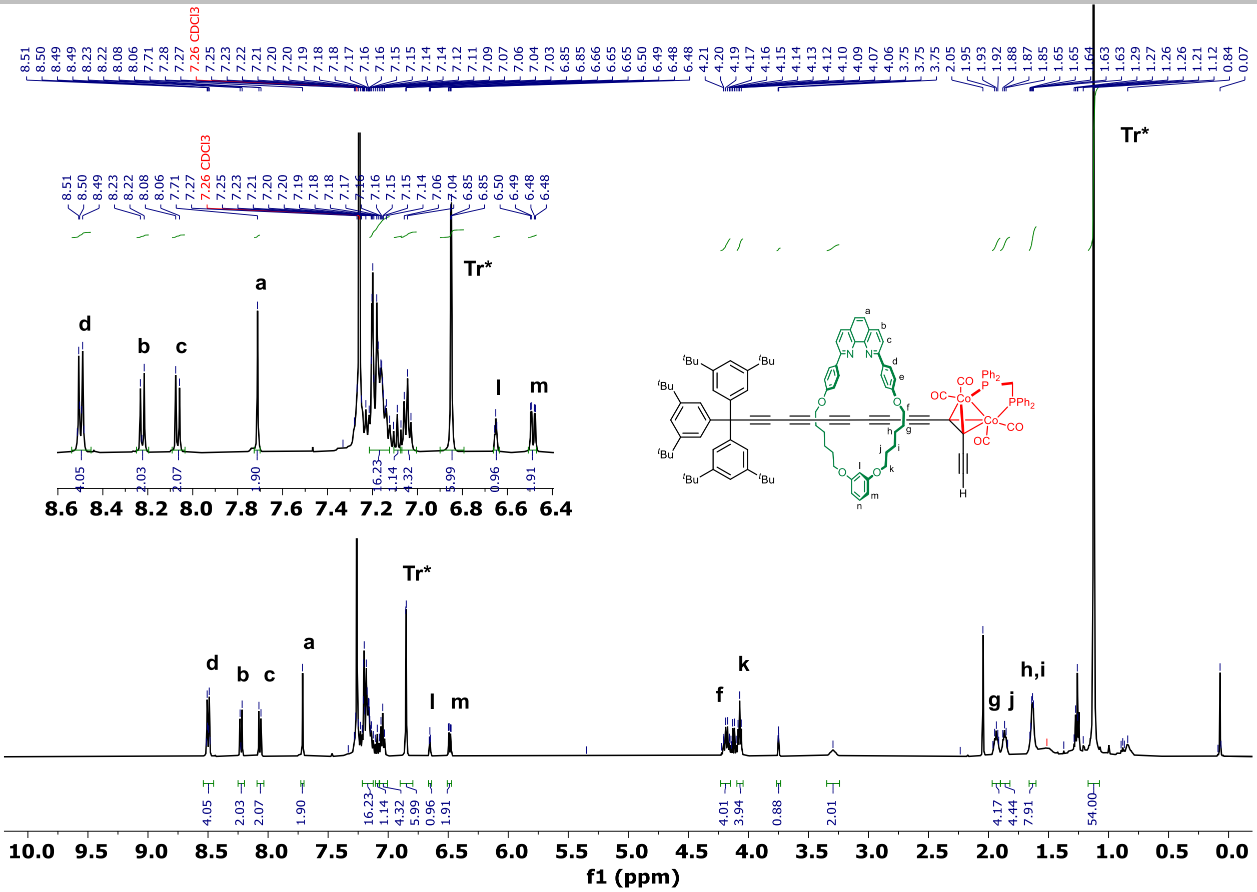

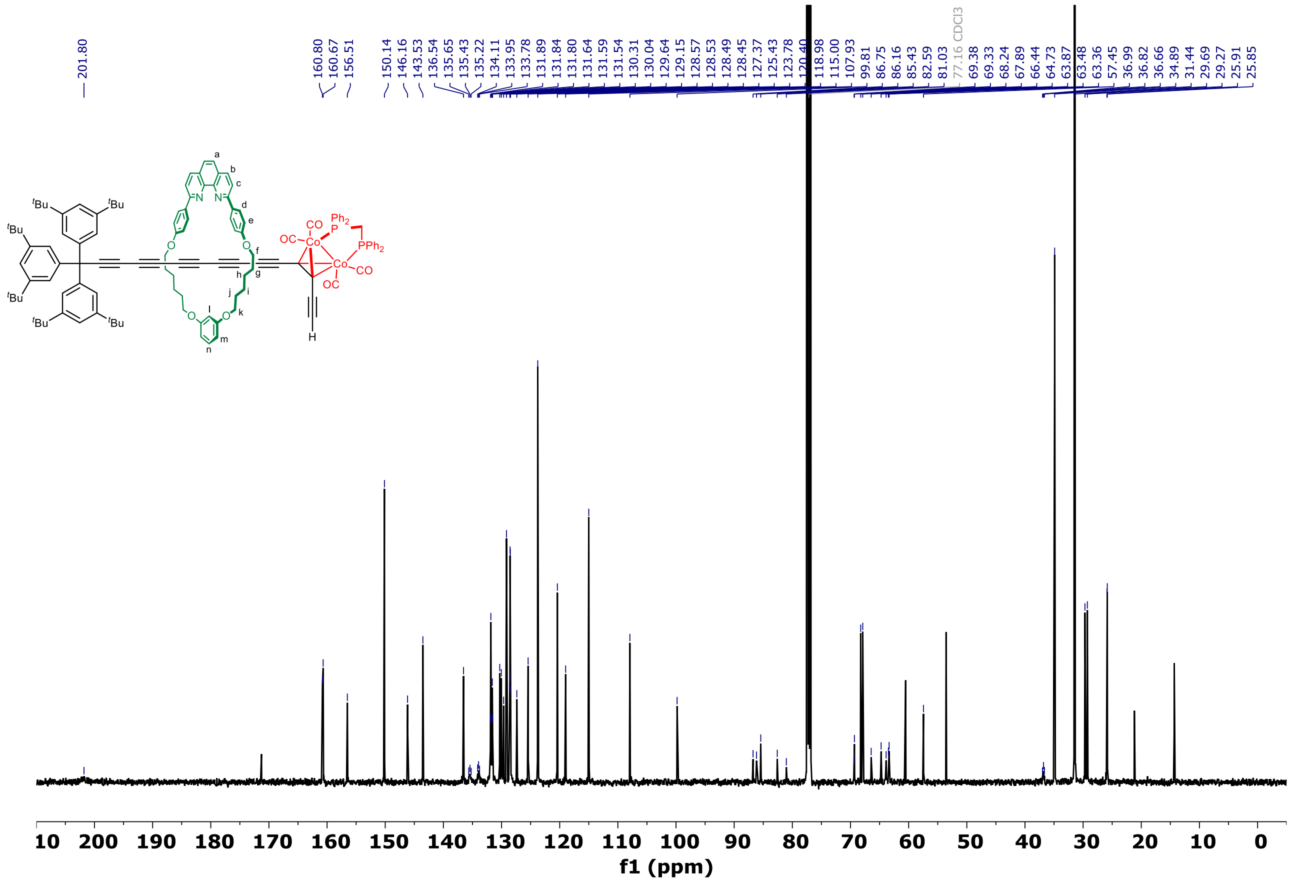

Figure S2: (top) ^{1}H NMR (500 MHz) and (bottom) ^{13}C NMR (126 MHz) spectra of deprotected rotaxane **5·M1** ($CDCl_3$, 298 K).

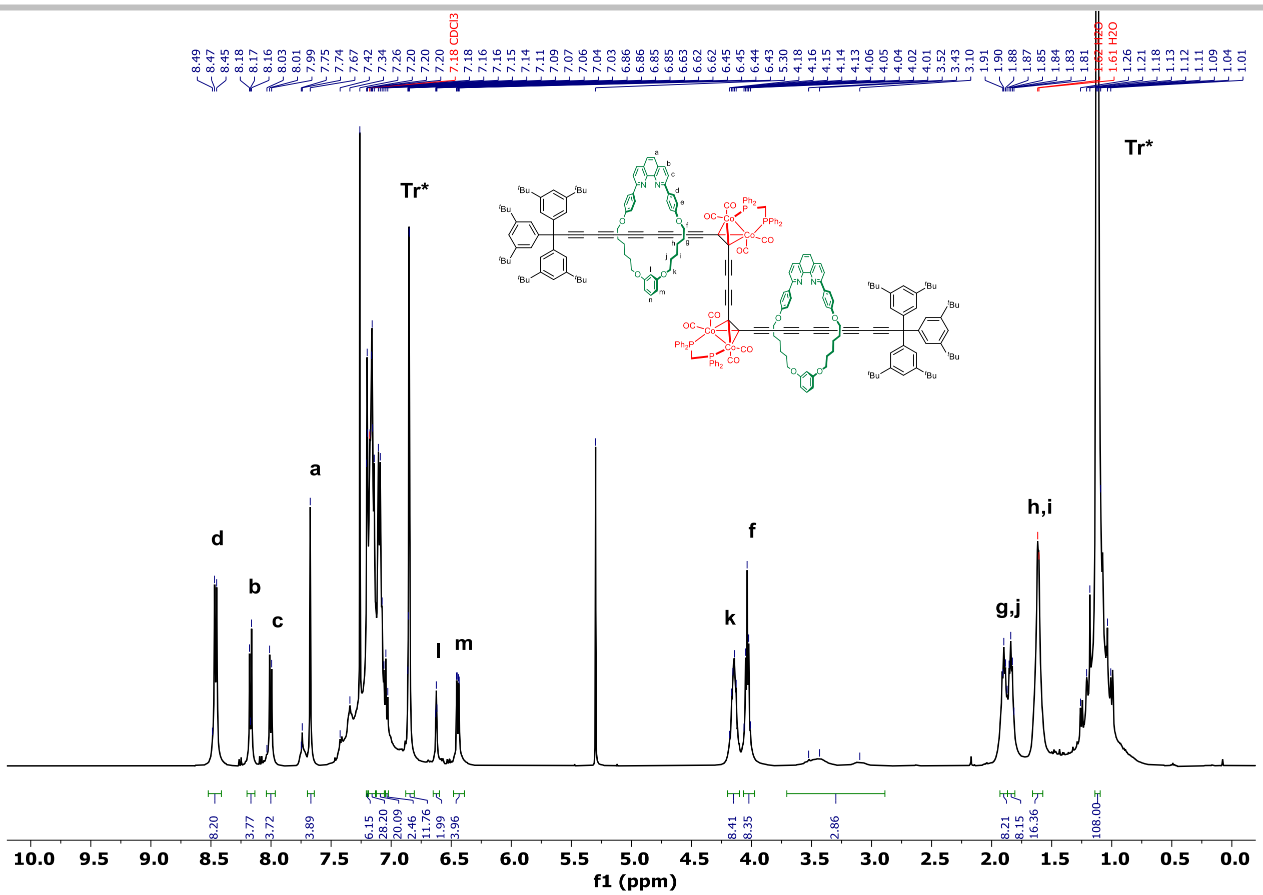

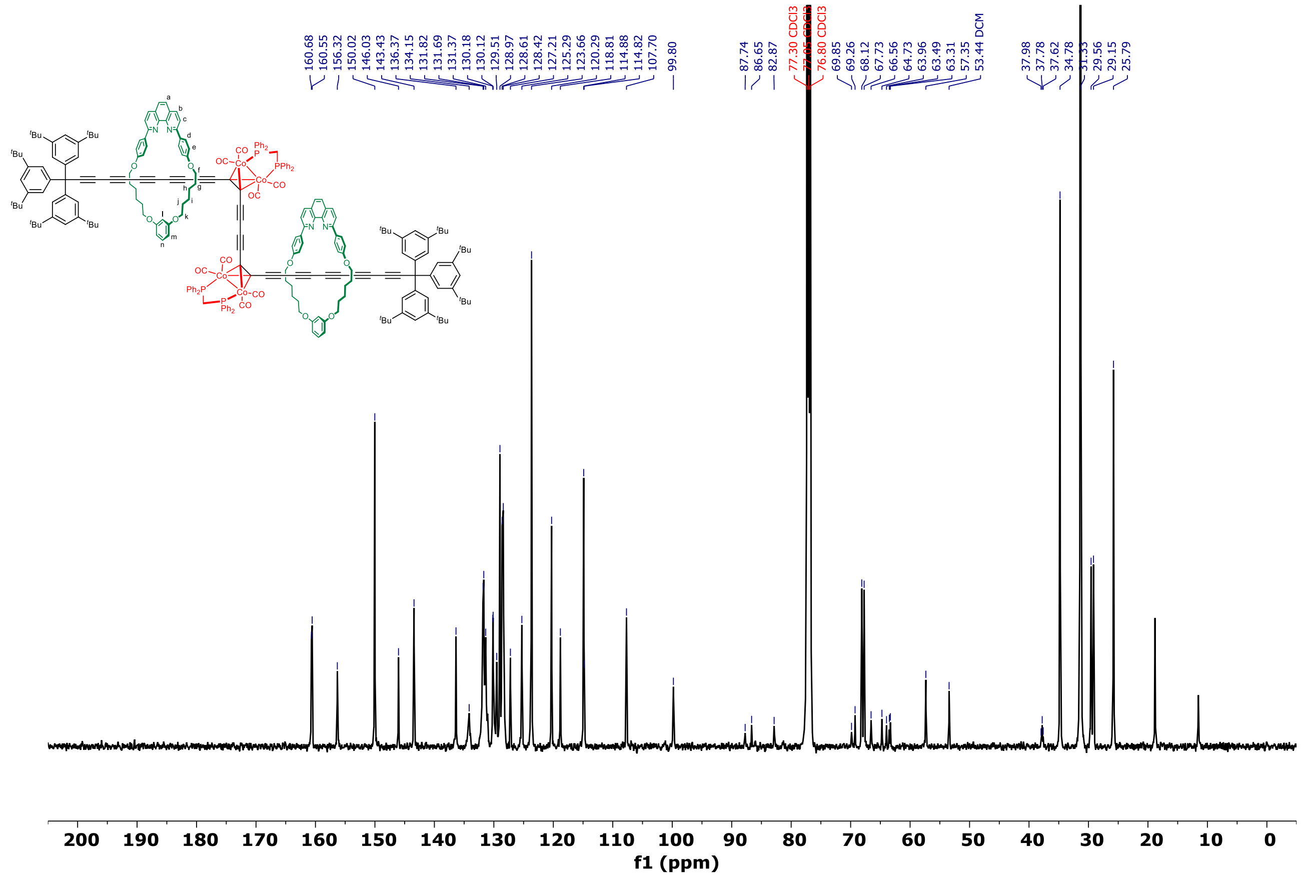

Figure S3: (top) ^{1}H NMR (500 MHz) and (bottom) ^{13}C NMR (126 MHz) spectra of supertrityl-cobalt [3]rotaxane **6**·(**M1**)$_2$ ($CDCl_3$, 298 K).

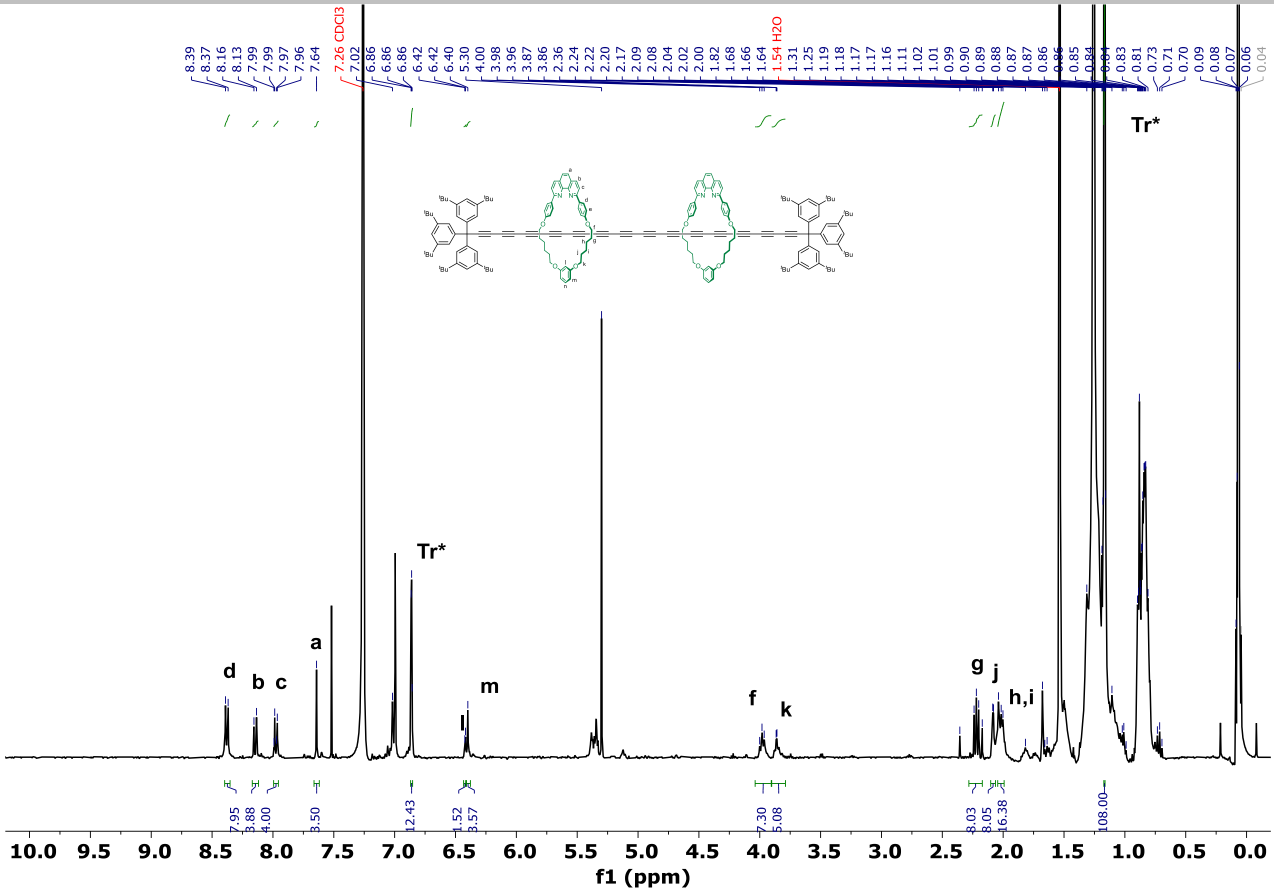

Figure S4: ^{1}H NMR (500 MHz) spectrum of supertrityl polyyne [3]rotaxane **1**·(**M1**)$_2$ ($CDCl_3$, 298 K).

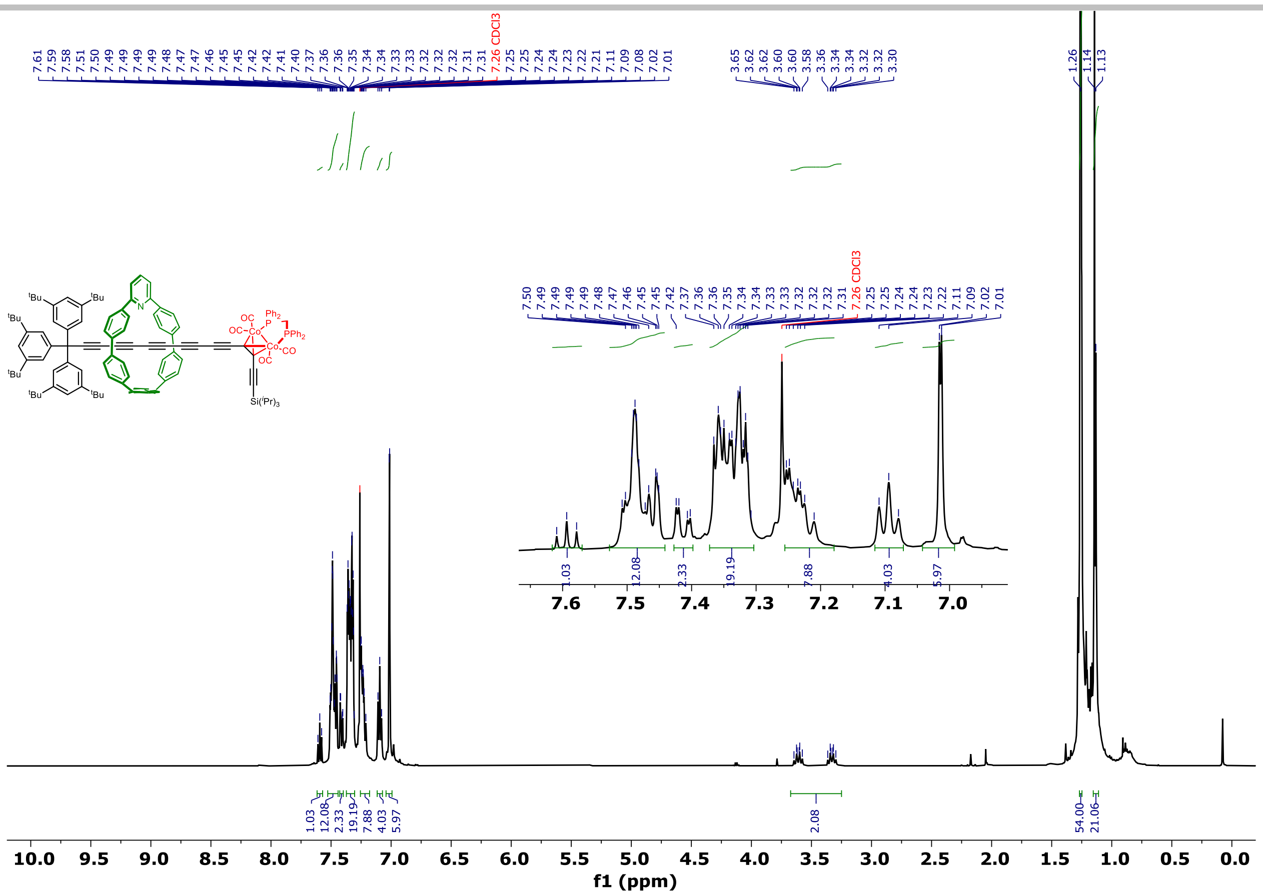

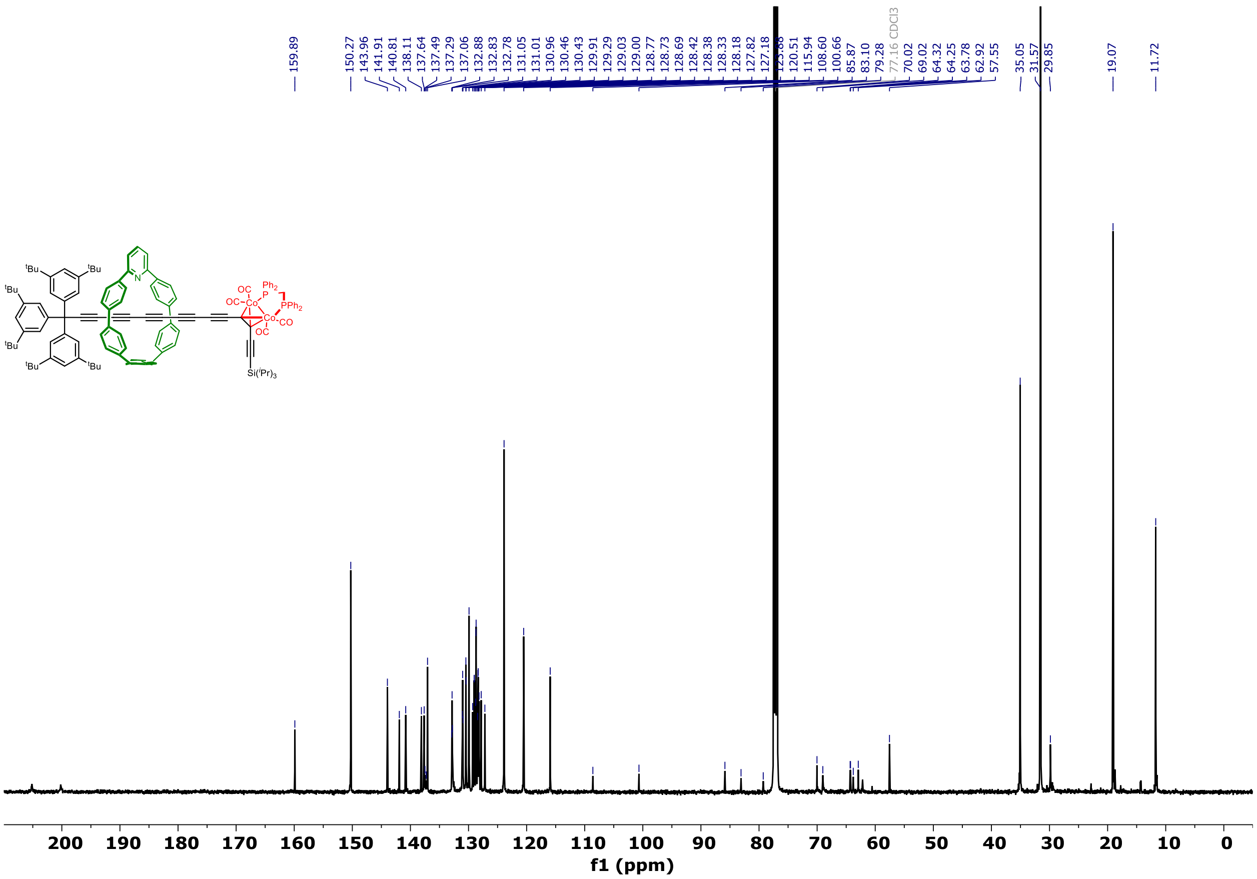

Figure S5: (top) ^{1}H NMR (500 MHz) and (bottom) ^{13}C NMR (126 MHz) spectra of supertrityl-cobalt [3]rotaxane **4·M2** ($CDCl_3$, 298 K).

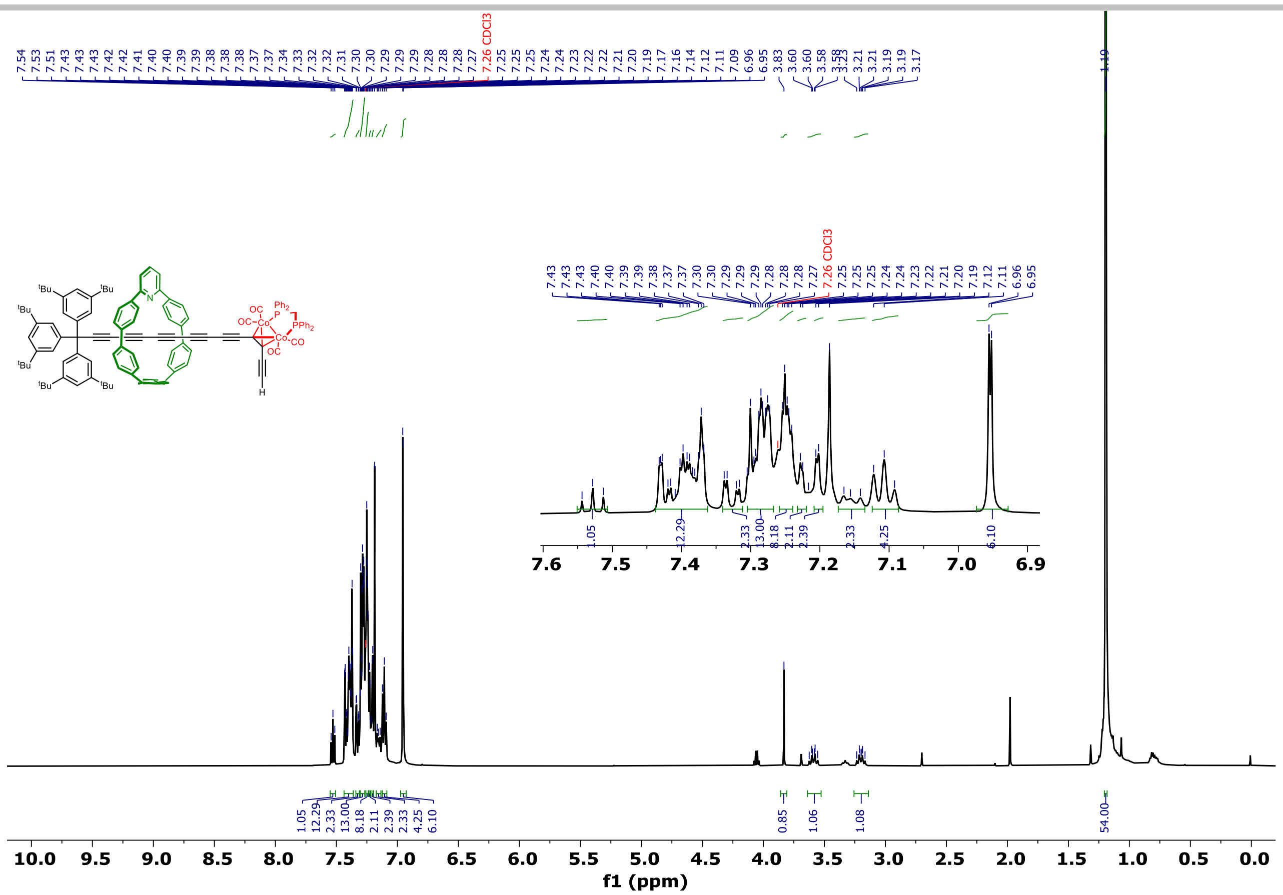

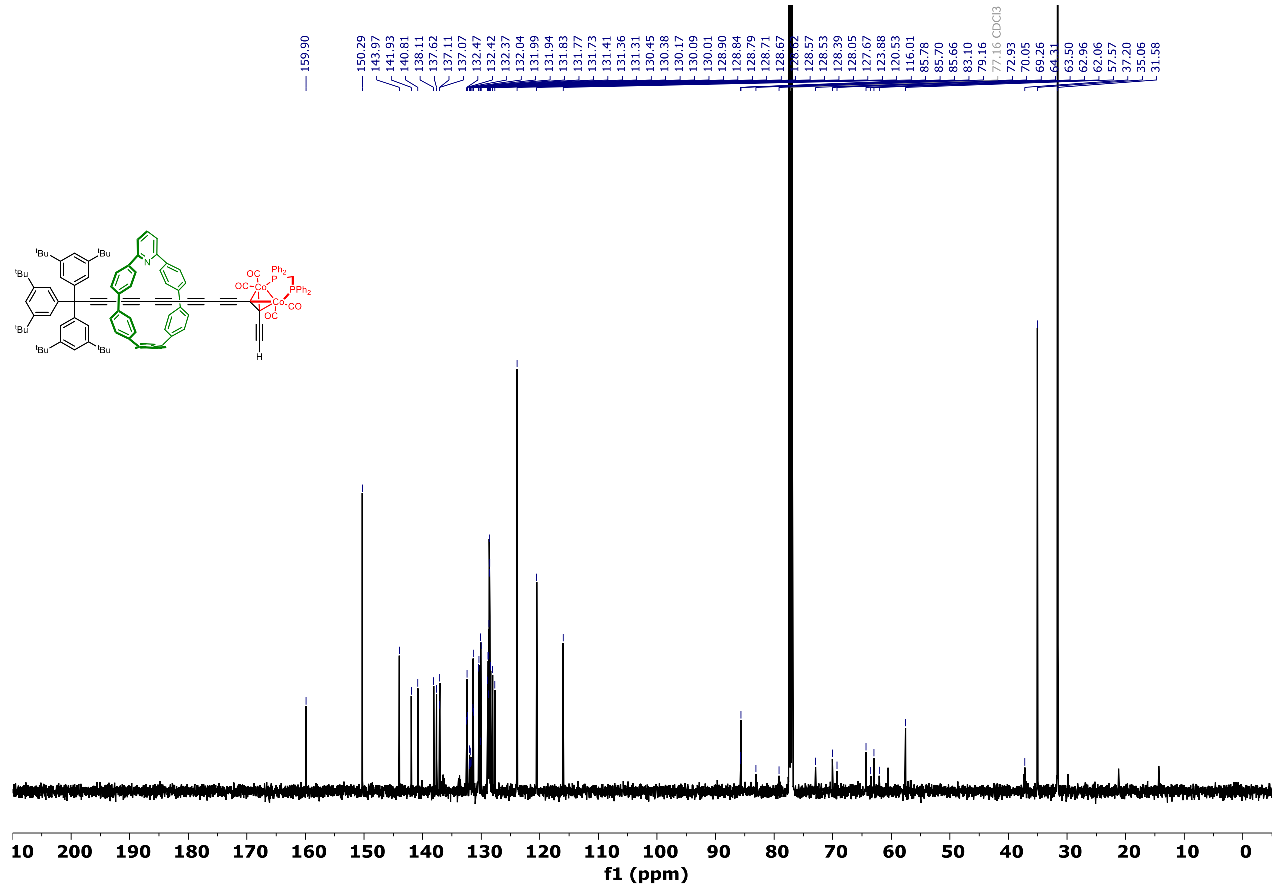

Figure S6: (top) ^{1}H NMR (500 MHz) and (bottom) ^{13}C NMR (126 MHz) spectra of deprotected nanohoop [2]rotaxane **5M·2** ($CDCl_3$, 298 K).

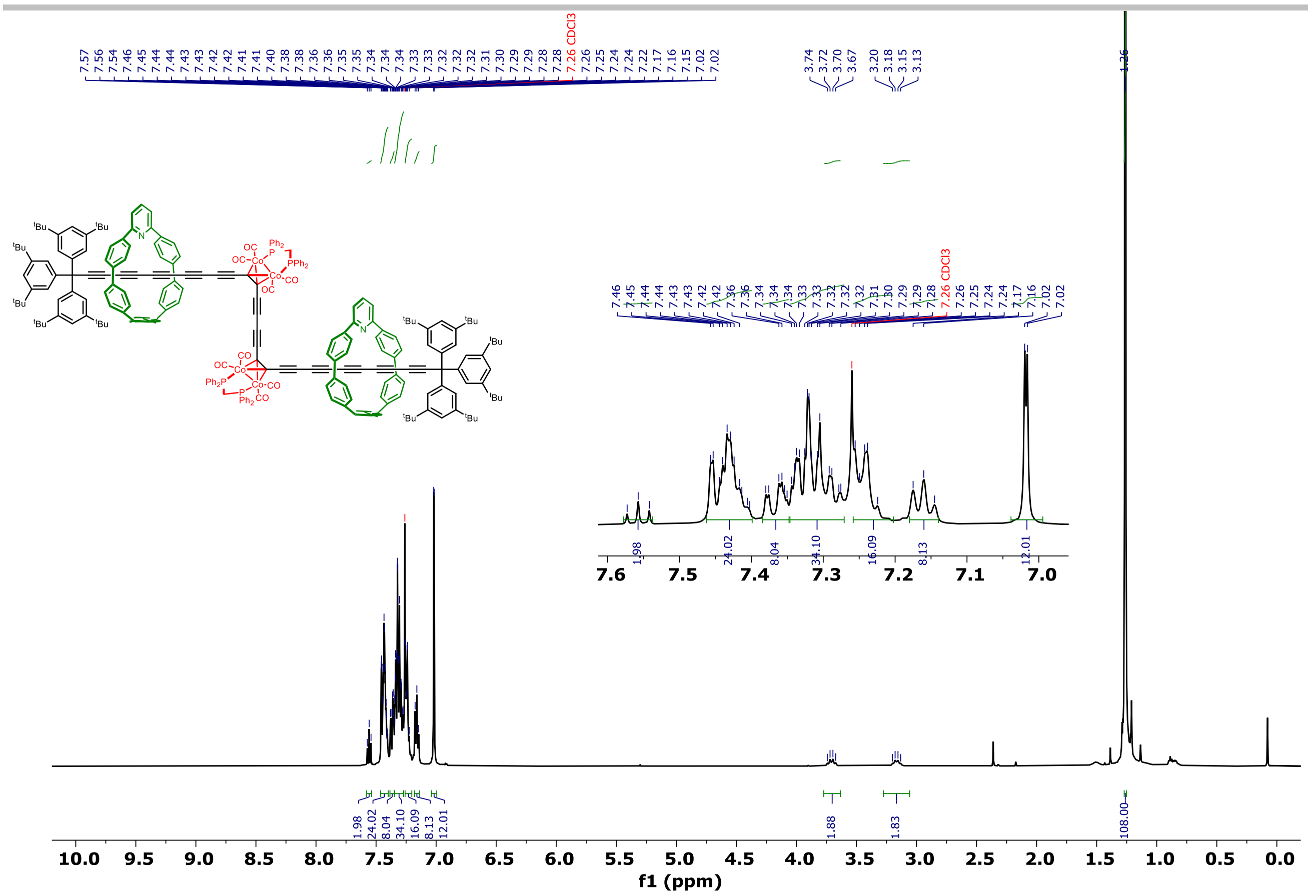

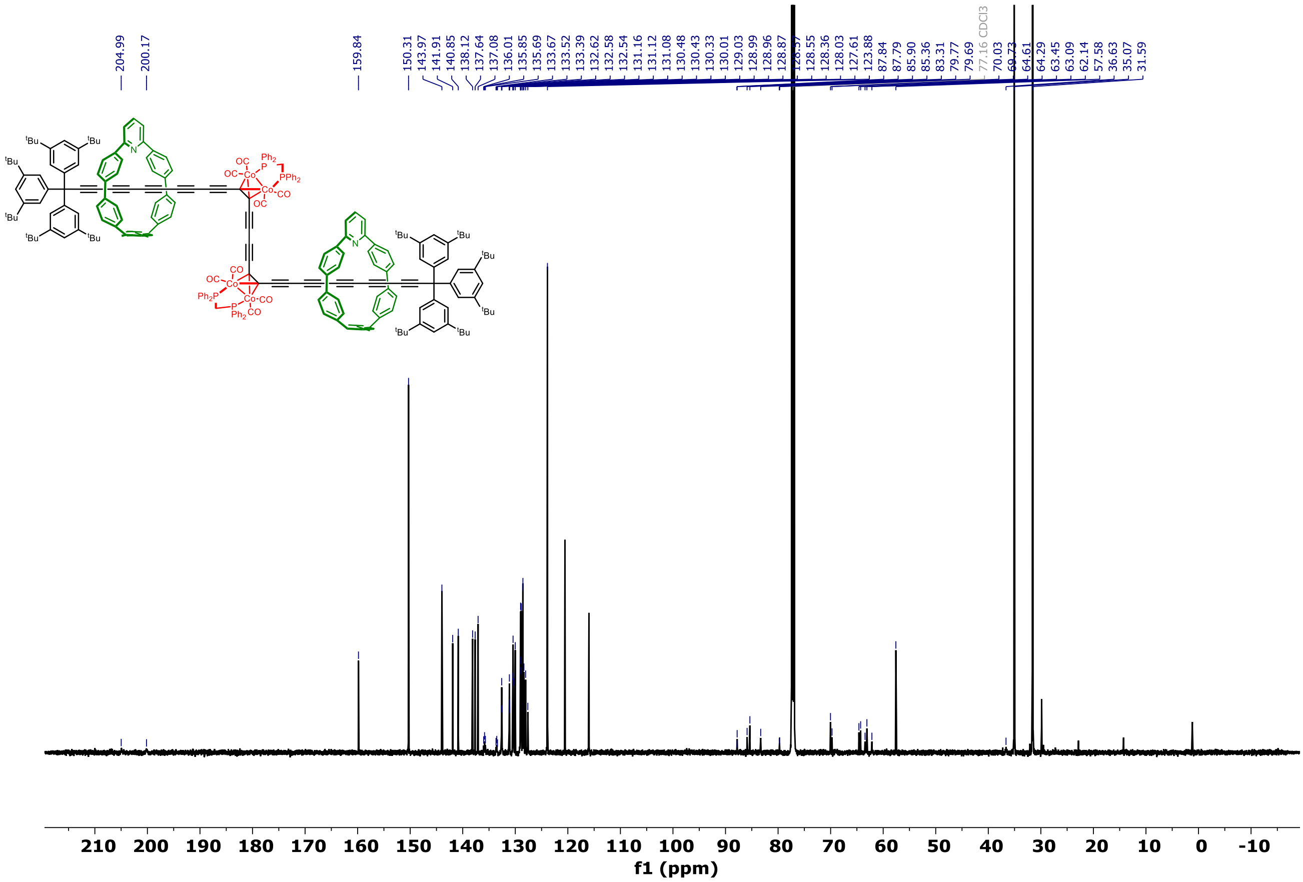

Figure S7: (top) ^{1}H NMR (600 MHz) and (bottom) ^{13}C NMR (151 MHz) spectra of masked nanohoop [3]rotaxane **6·(M2)**$_2$ ($CDCl_3$, 298 K).

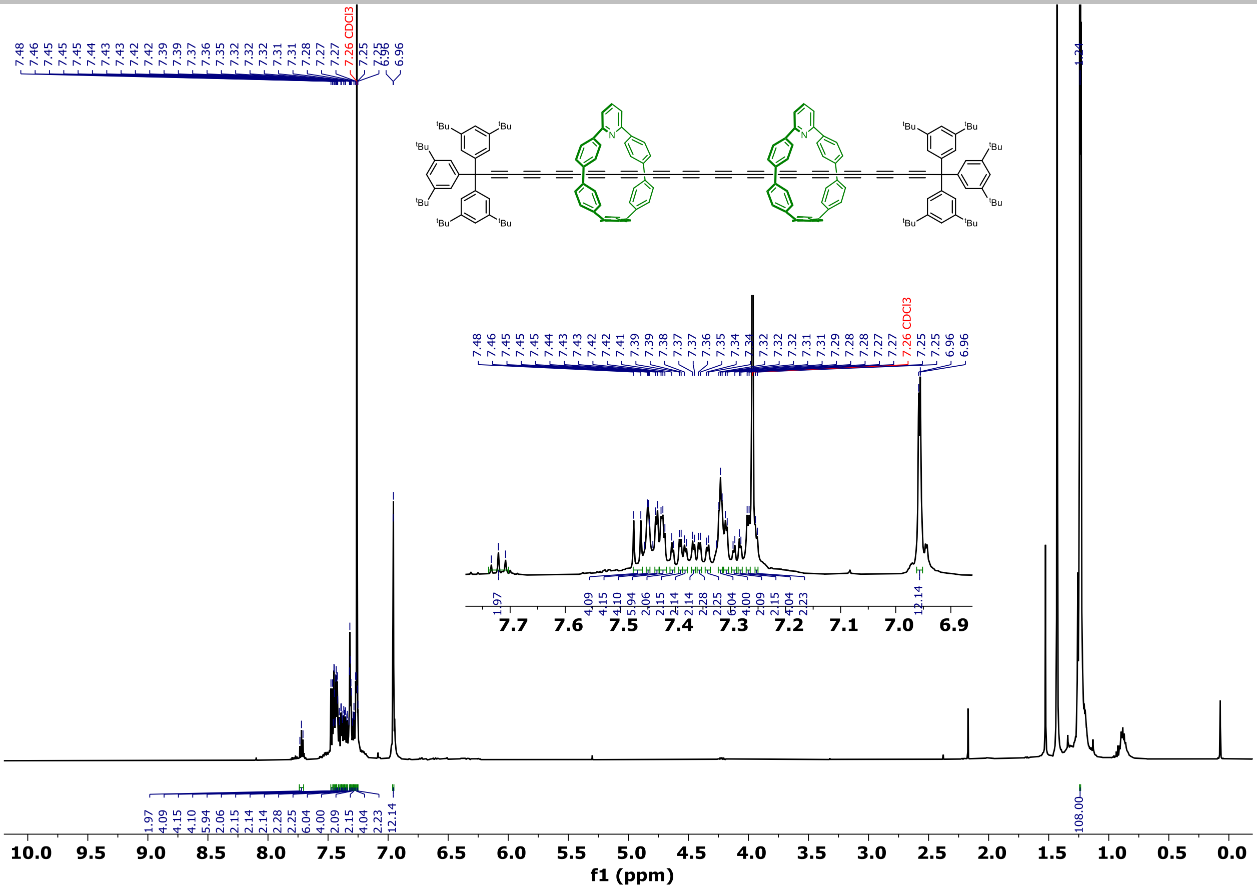

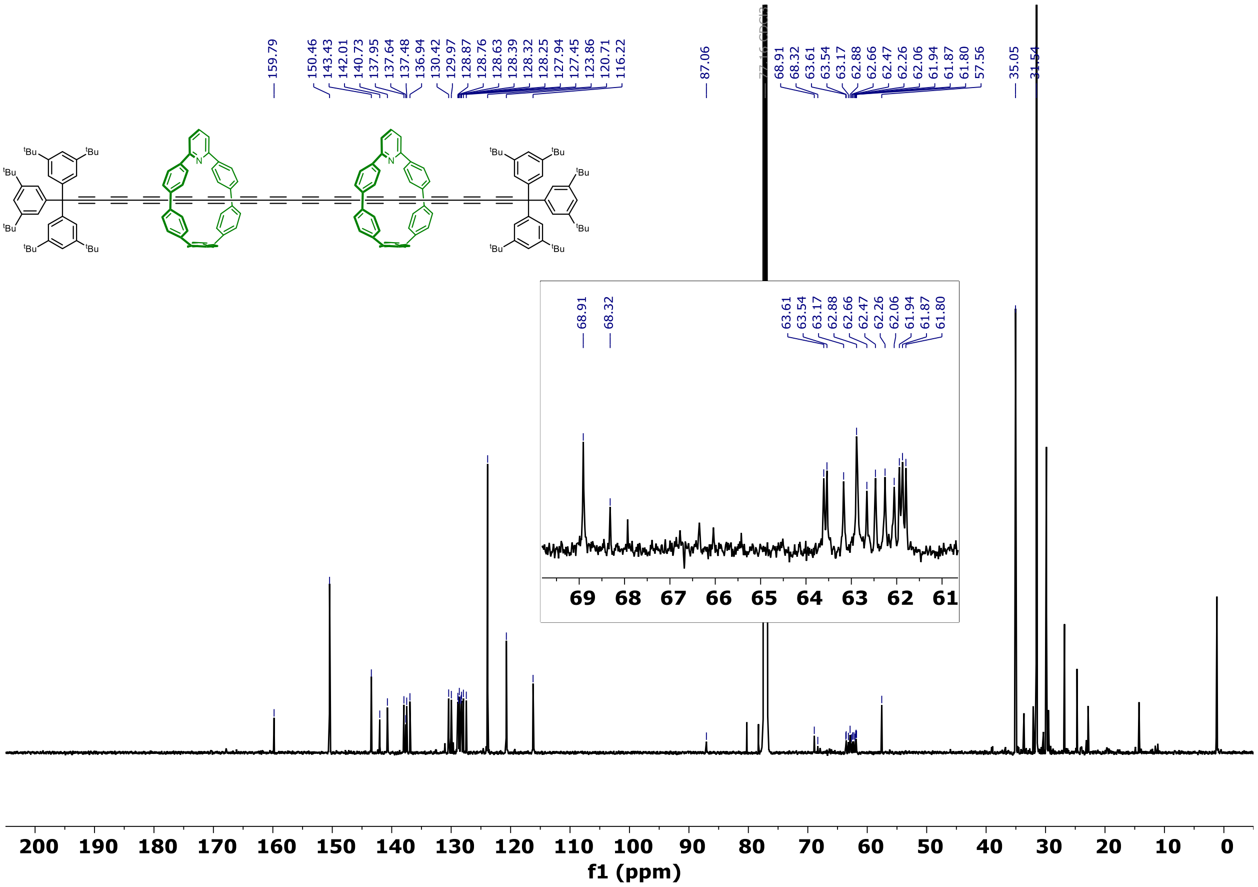

Figure S8: (top) ^{1}H NMR (600 MHz) and (bottom) ^{13}C NMR (151 MHz) spectra of nanohoop polyyne [3]rotaxane **1**·(**M2**)$_2$ ($CDCl_3$, 298 K).

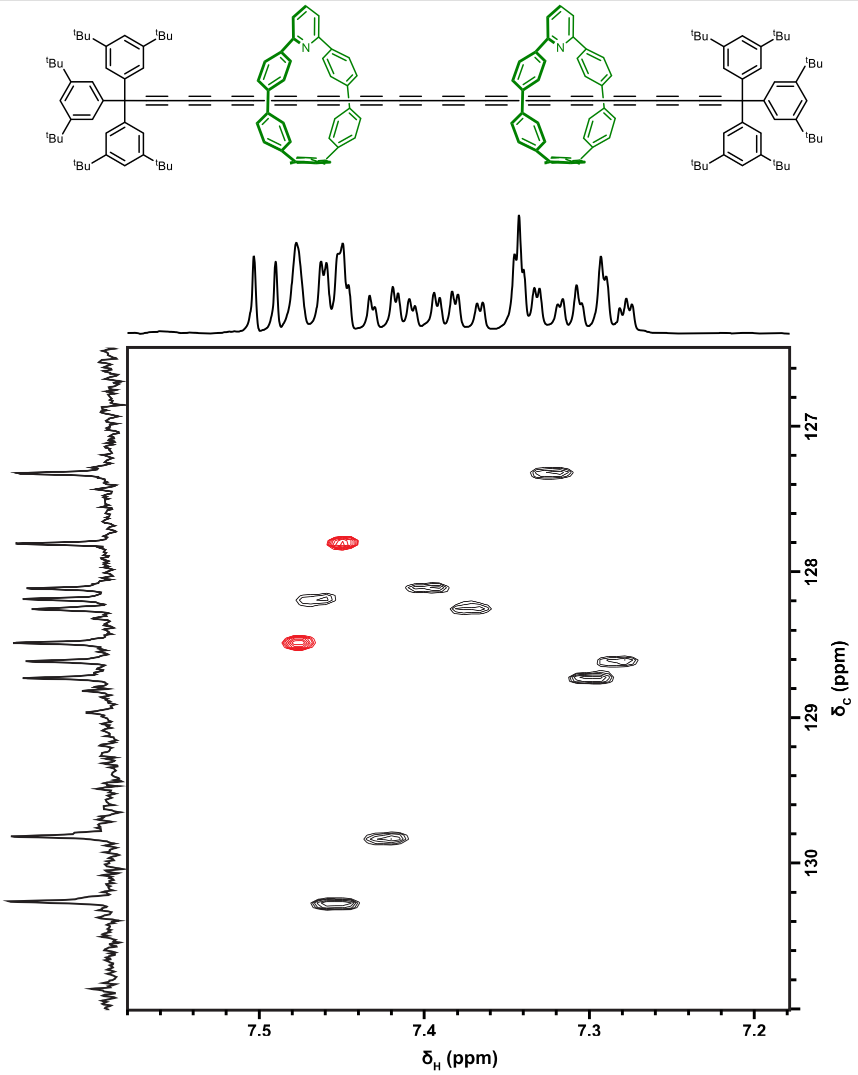

Figure S9: $^{1}H/^{13}C$ high-resolution HSQC spectrum ($CDCl_3$, 298 K at 700 MHz) of nanohoop polyyne [3]rotaxane **1·(M2)**$_2$. Cross peaks arising from the middle *para*-phenylene, furthest away from the pyridine unit, have been colored red. The ^{1}H reference spectrum shown has been diffusion edited to attenuate the overlapping $CHCl_3$ resonance. Editing used the 1D double stimulated echo sequence (DSTEBGP3S1D) with a diffusion time Δ of 100 ms, and gradient pulse duration δ of 1.5 ms at 30 G/cm.

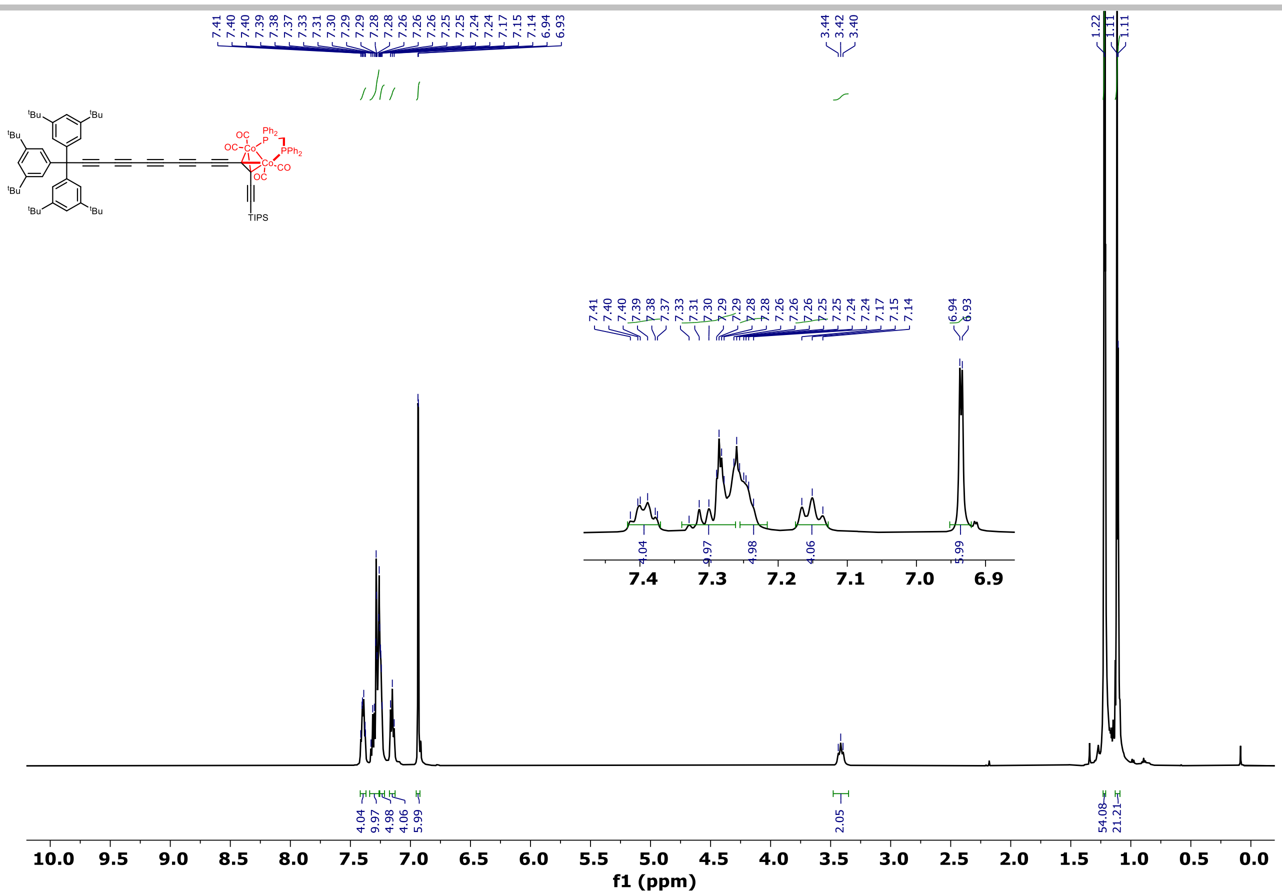

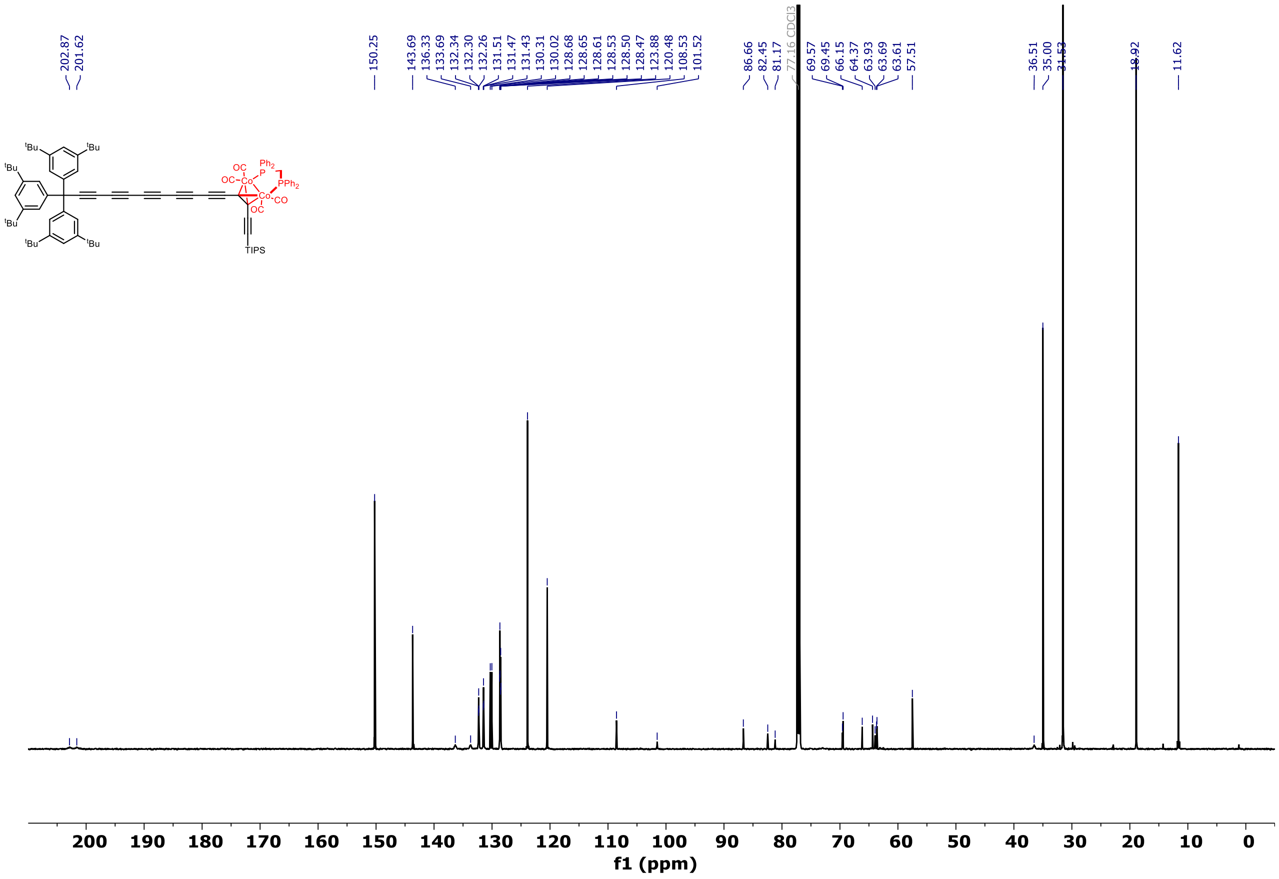

Figure S10: (top) ^{1}H NMR (500 MHz) and (bottom) ^{13}C NMR (151 MHz) spectra of TIPS-protected [2]rotaxane thread **4** ($CDCl_3$, 298 K).

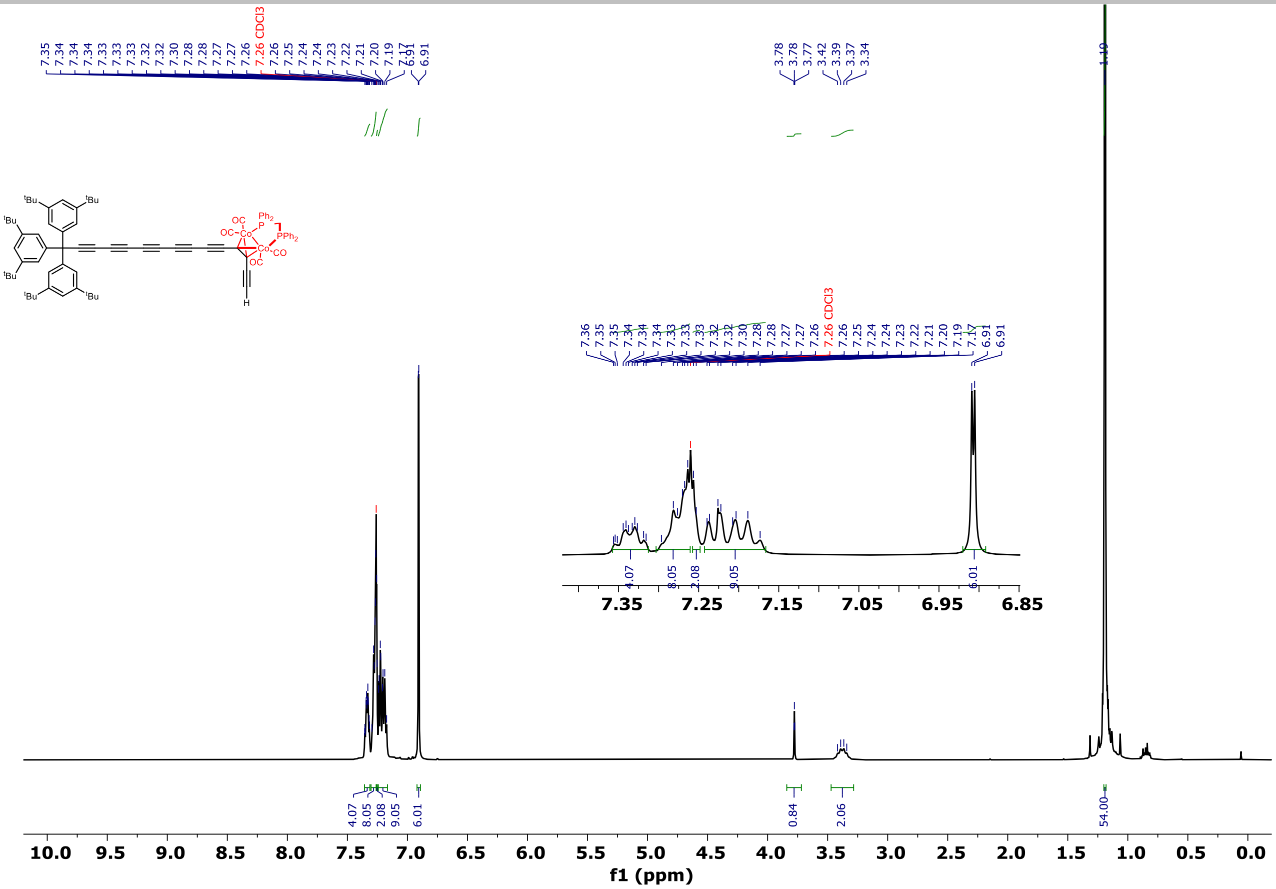

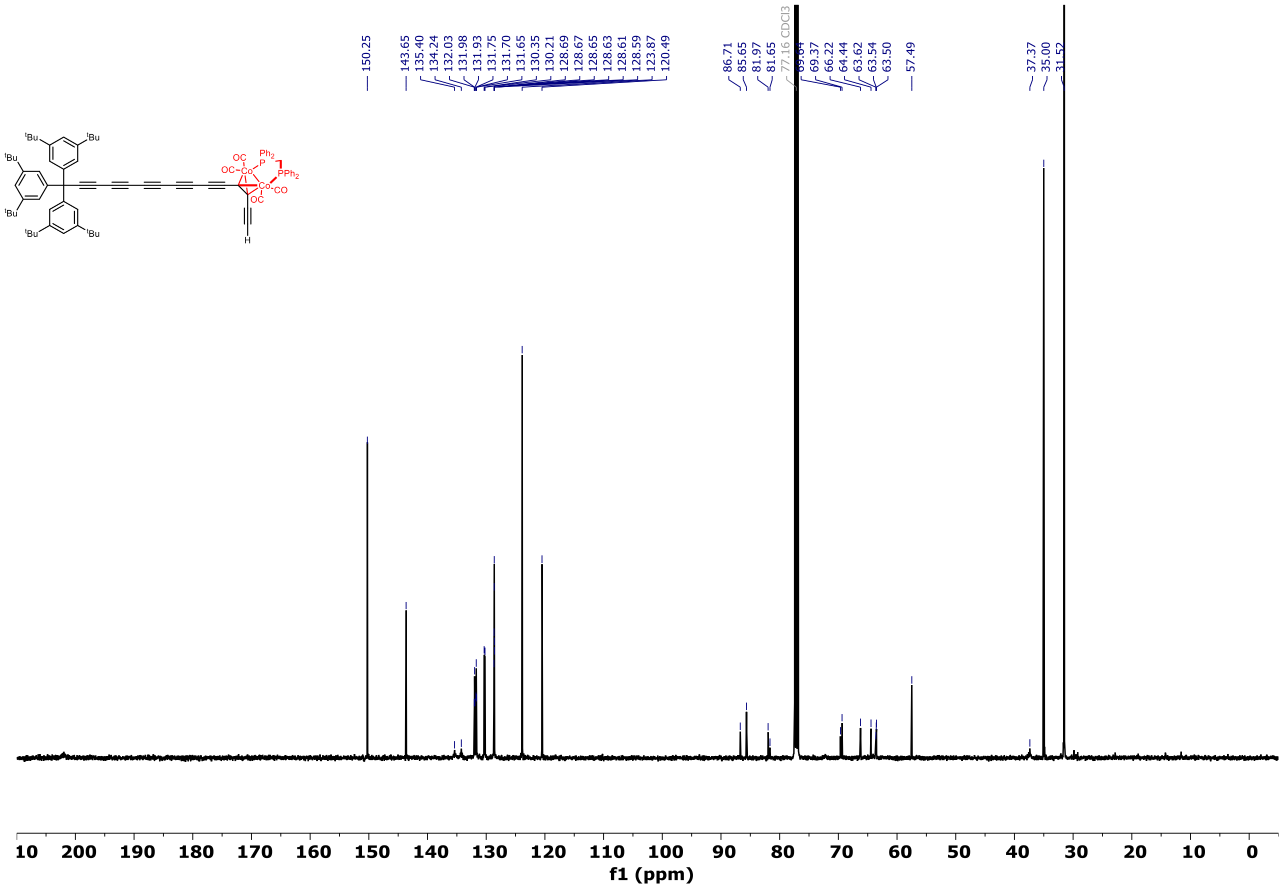

Figure S11: (top) ^{1}H NMR (500 MHz) and (bottom) ^{13}C NMR (126 MHz) spectra of deprotected [2]rotaxane thread **5** ($CDCl_3$, 298 K).

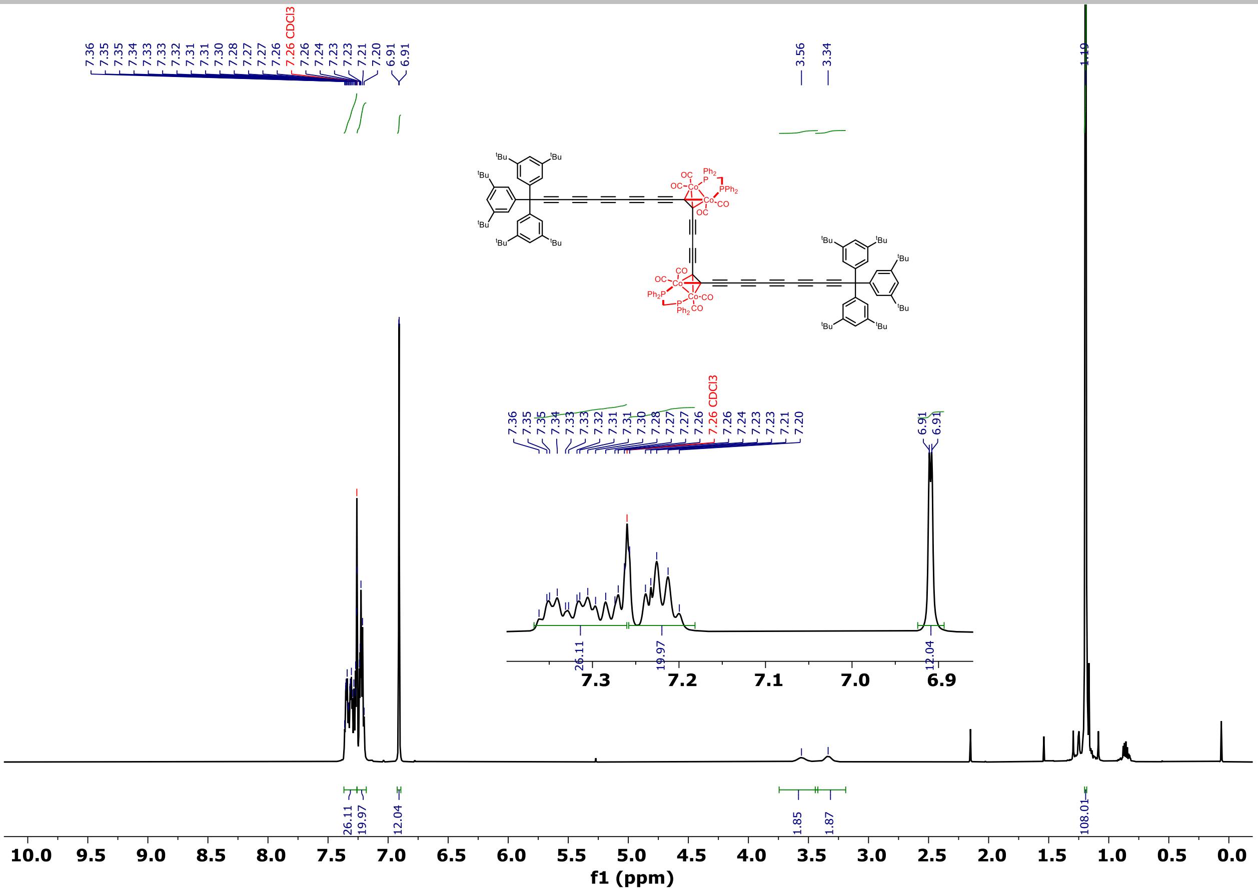

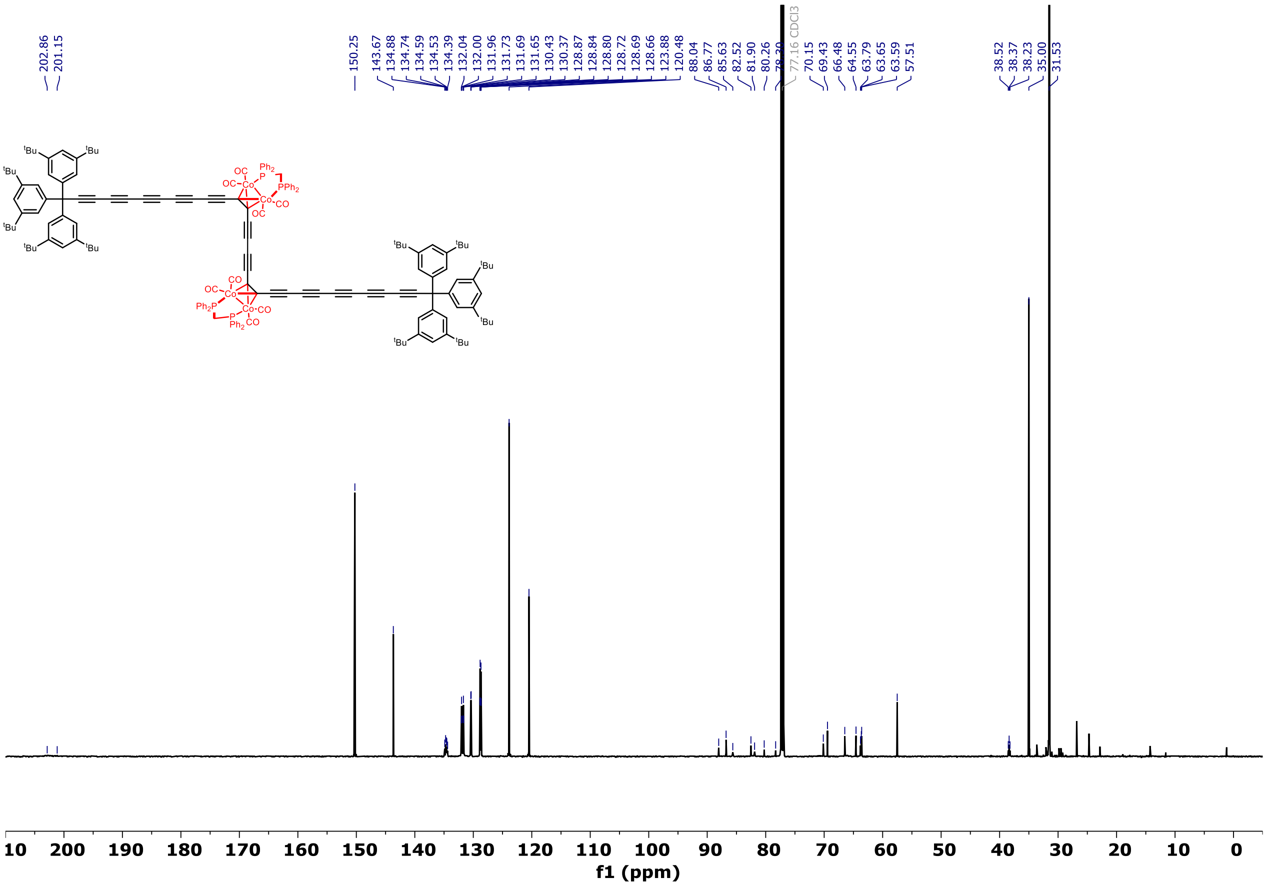

Figure S12: (top) ^{1}H NMR (500 MHz) and (bottom) ^{13}C NMR (126 MHz) spectra of masked [3]rotaxane thread **6** ($CDCl_3$, 298 K).

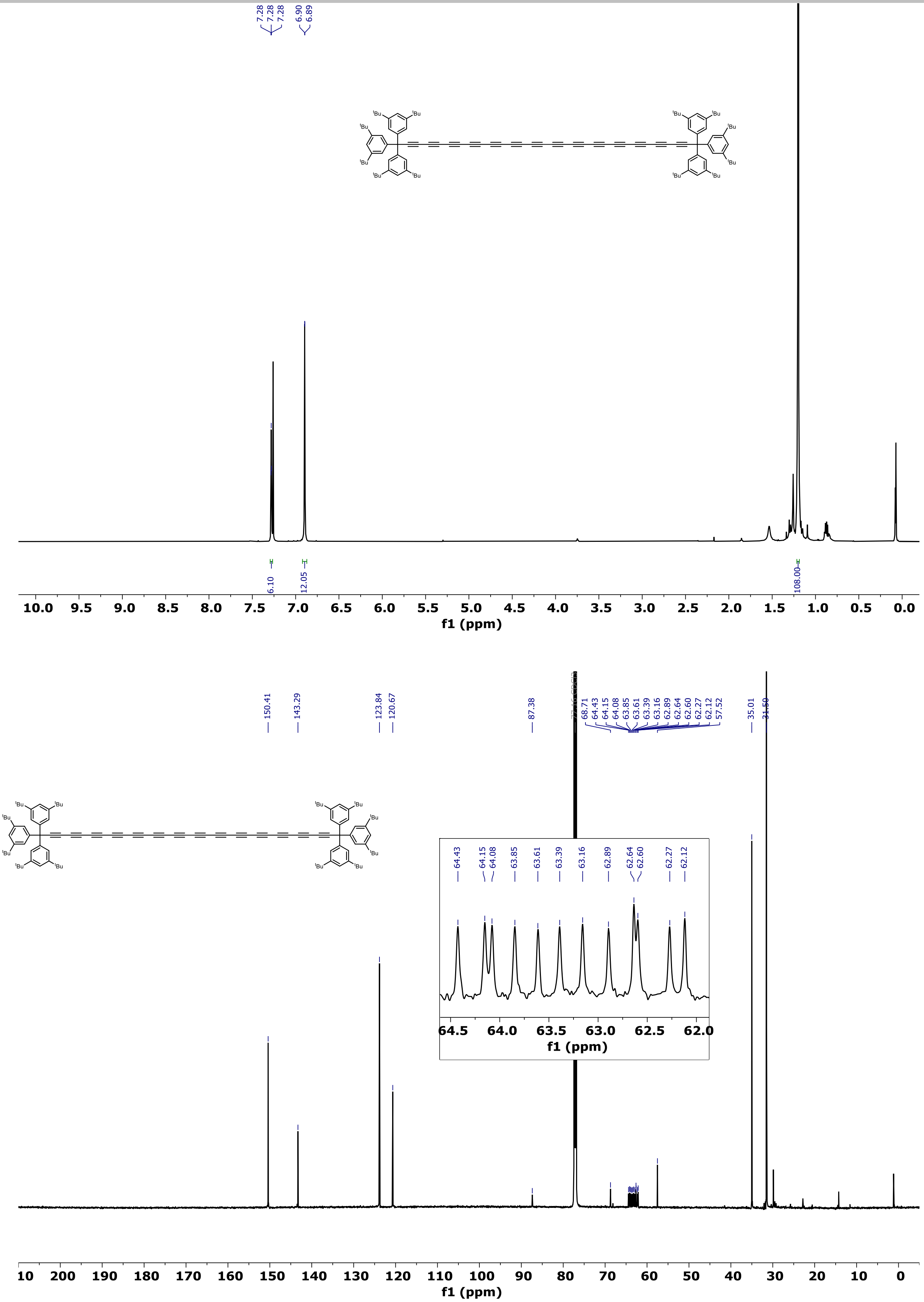

Figure S13: (top) ^{1}H NMR (600 MHz) and (bottom) ^{13}C NMR (151 MHz) spectra of polyyne dumbbell thread **1** ($CDCl_3$, 298 K).

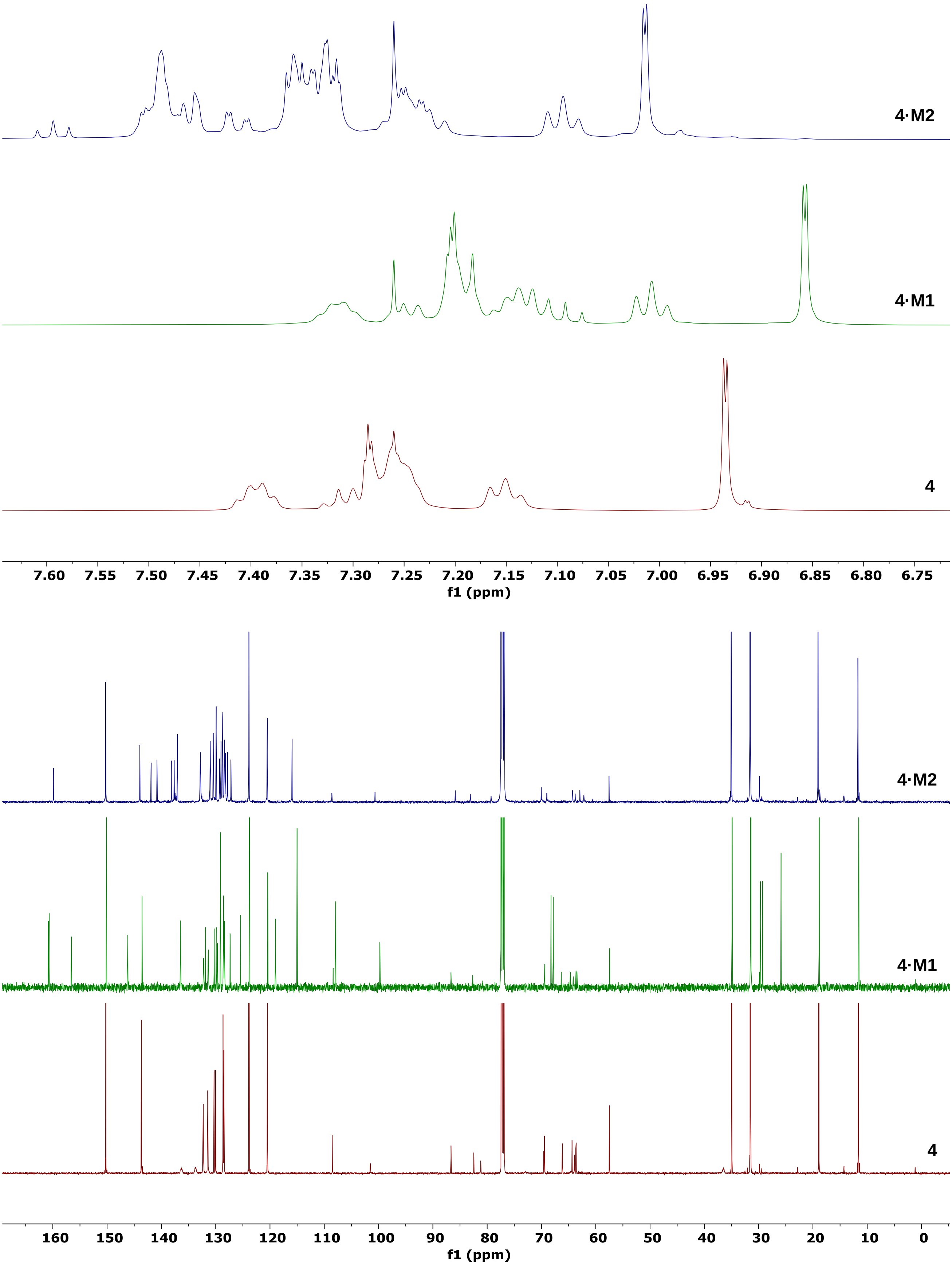

Figure S14: Stacked (top) ^{1}H NMR (500 MHz) and (bottom) ^{13}C NMR (126 MHz) spectra of (red) thread **4**, (green) [2]rotaxane **4·M1** and (blue) [2]rotaxane **4·M2** ($CDCl_3$, 298 K).

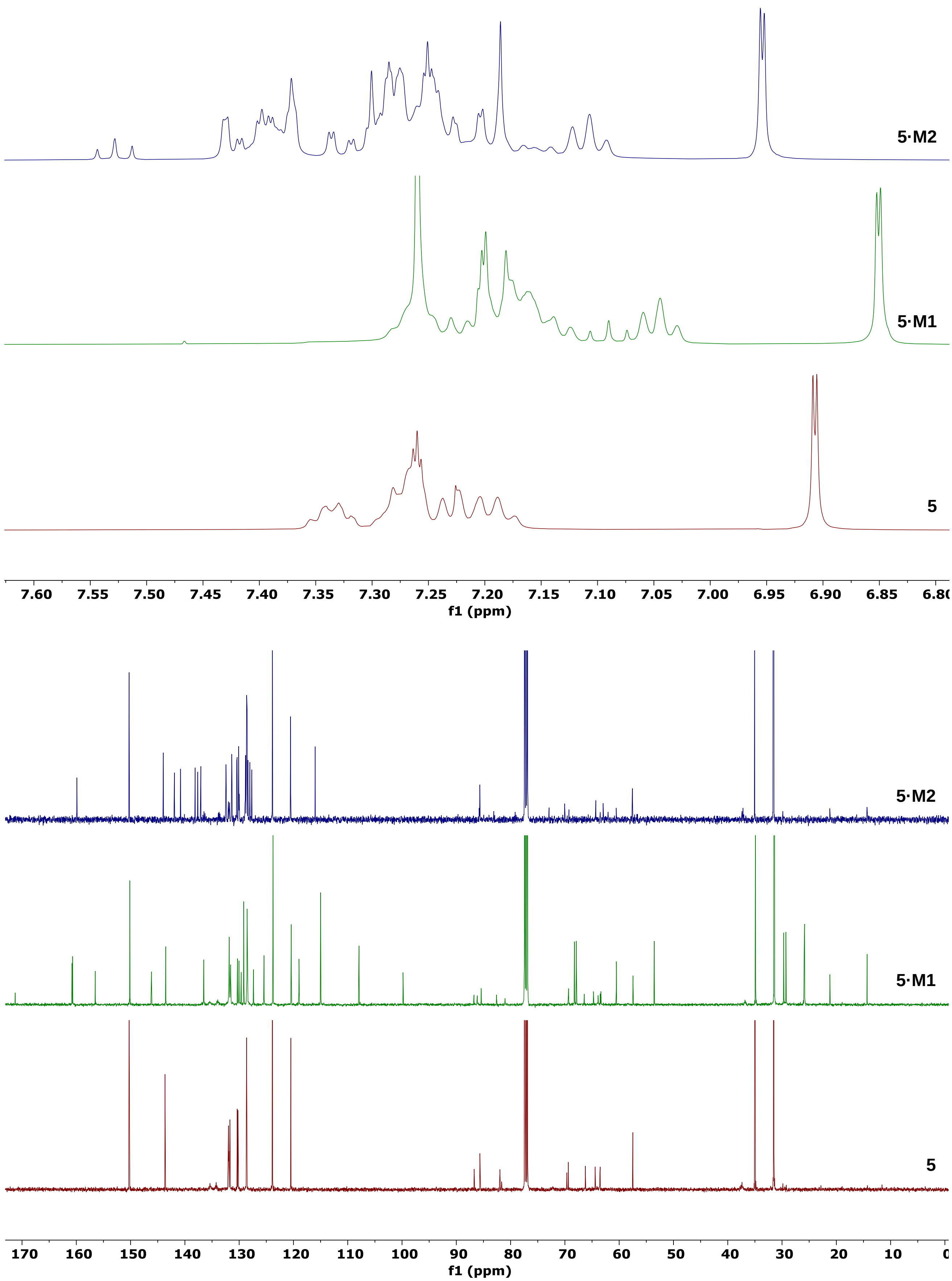

Figure S15: Stacked (top) ^{1}H NMR (500 MHz) and (bottom) ^{13}C NMR (126 MHz) spectra of (red) thread **5**, (green) [2]rotaxane **5·M1** and (blue) [2]rotaxane **5·M2** ($CDCl_3$, 298 K).

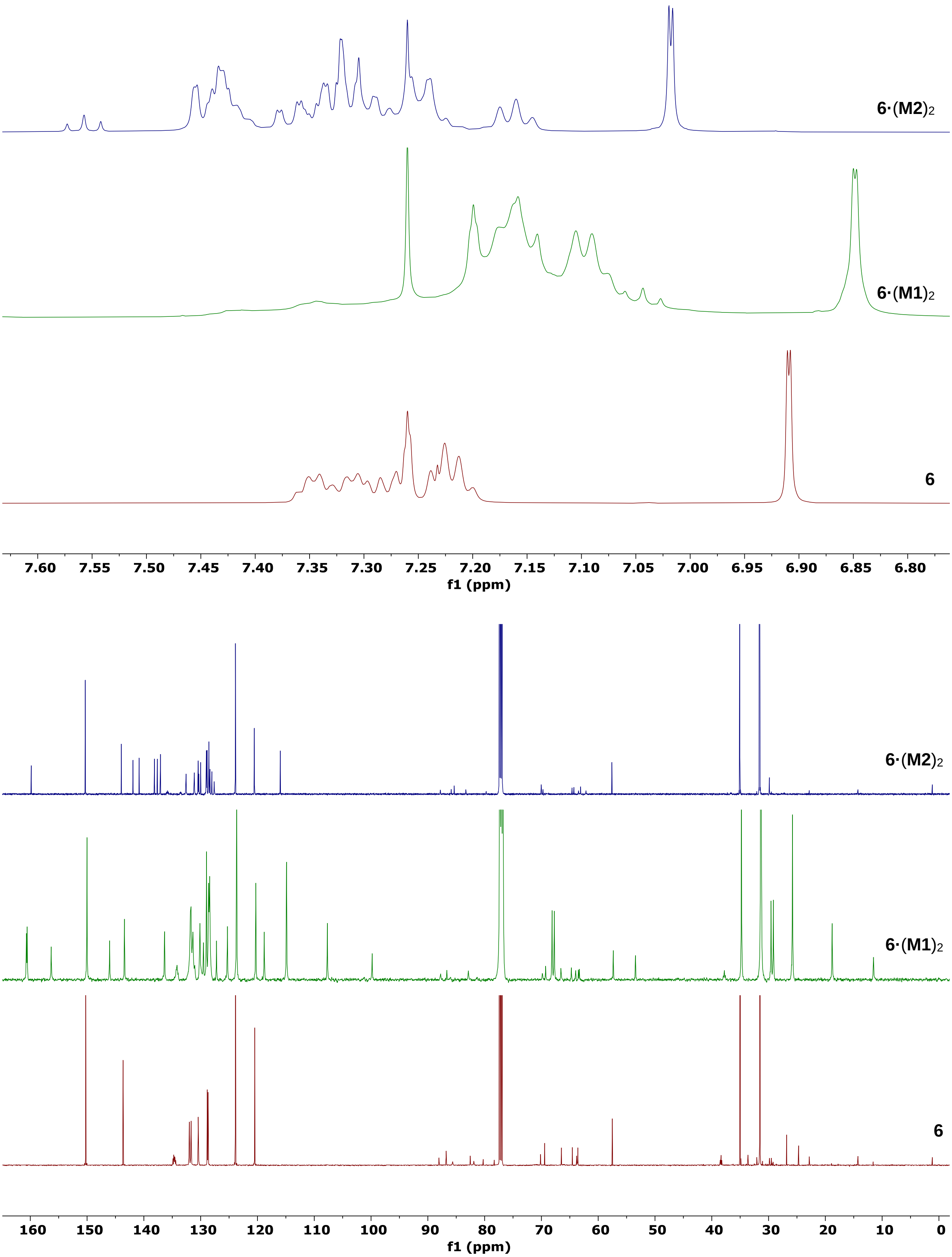

Figure S16: Stacked (top) ^{1}H NMR and (bottom) ^{13}C NMR spectra of (red) thread **6** (600/151 MHz), (green) [3]rotaxane **6**·(**M1**)$_2$ (500/126 MHz) and (blue) [3]rotaxane **6**·(**M2**)$_2$ (600/151 MHz) ($CDCl_3$, 298 K).

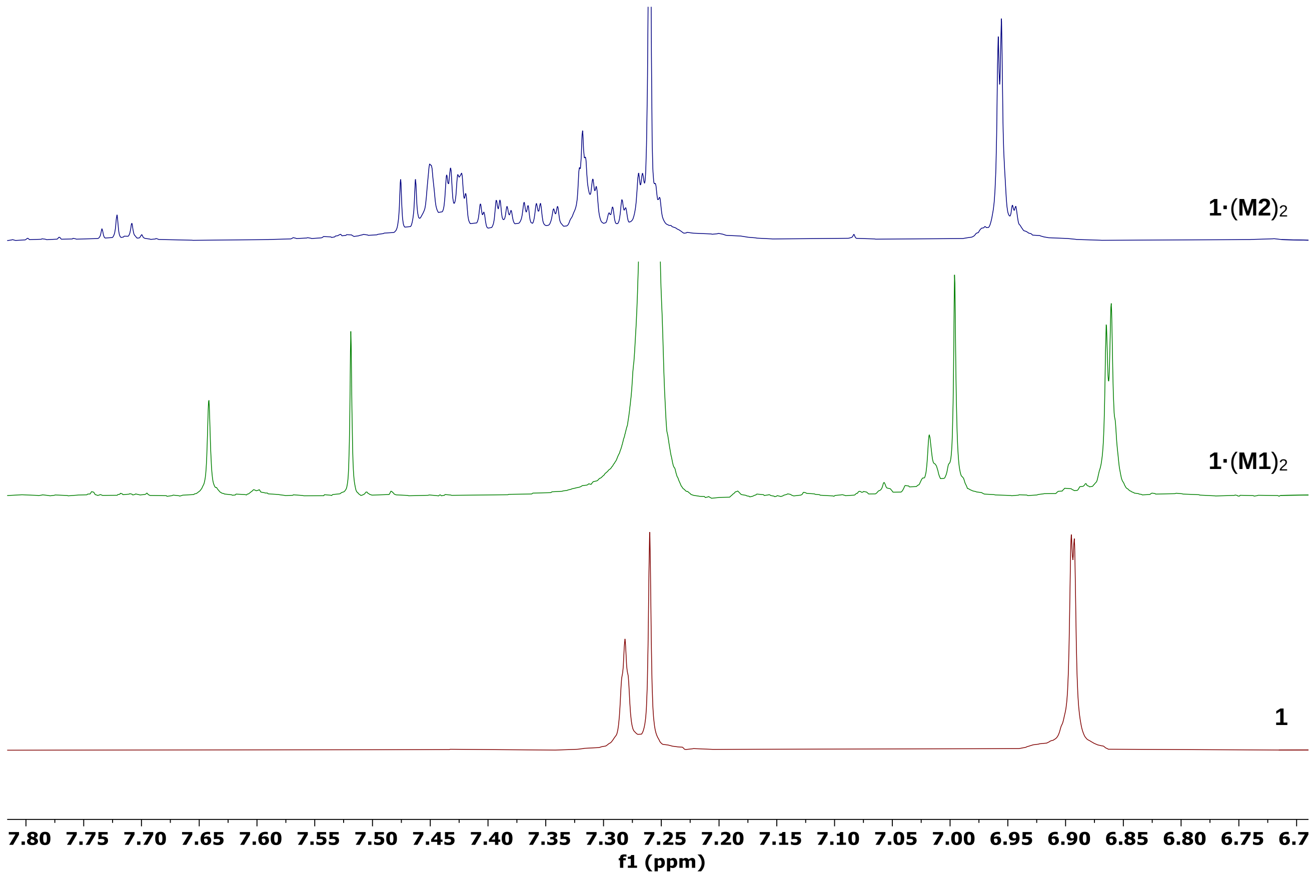

Figure S17: Stacked ^{1}H NMR spectra of (red) dumbbell thread **1** (600 MHz), (green) [3]rotaxane **1**·(**M1**)$_2$ (500 MHz) and (blue) [3]rotaxane **1**·(**M2**)$_2$ (600 MHz) ($CDCl_3$, 298 K).

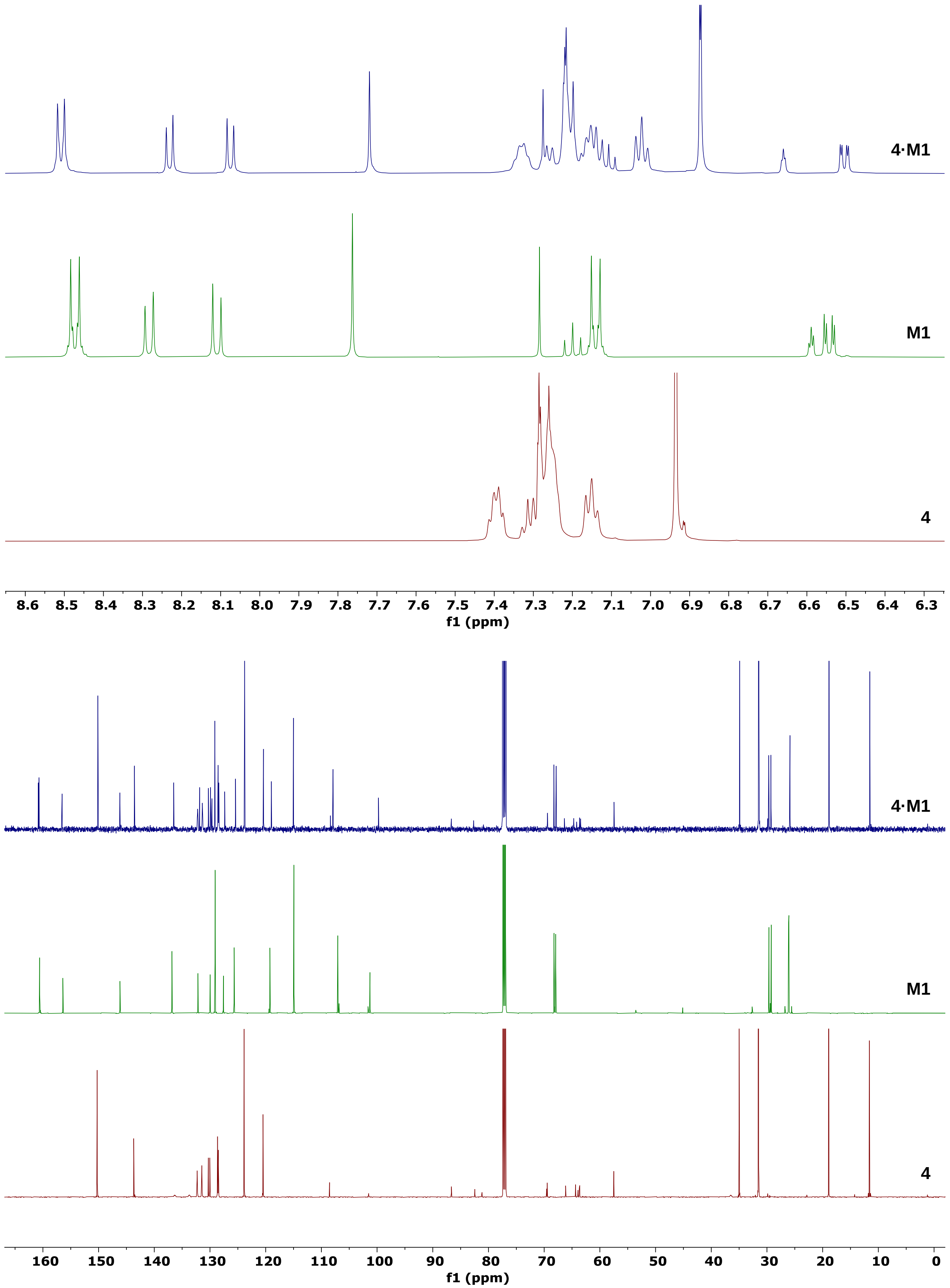

Figure S18: Stacked (top) ^{1}H NMR and (bottom) ^{13}C NMR spectra of (red) thread **4** (500/126 MHz), (green) macrocycle **M1** (600/151 MHz) and (blue) [2]rotaxane **4**·**M1** (500/126 MHz) ($CDCl_3$, 298 K).

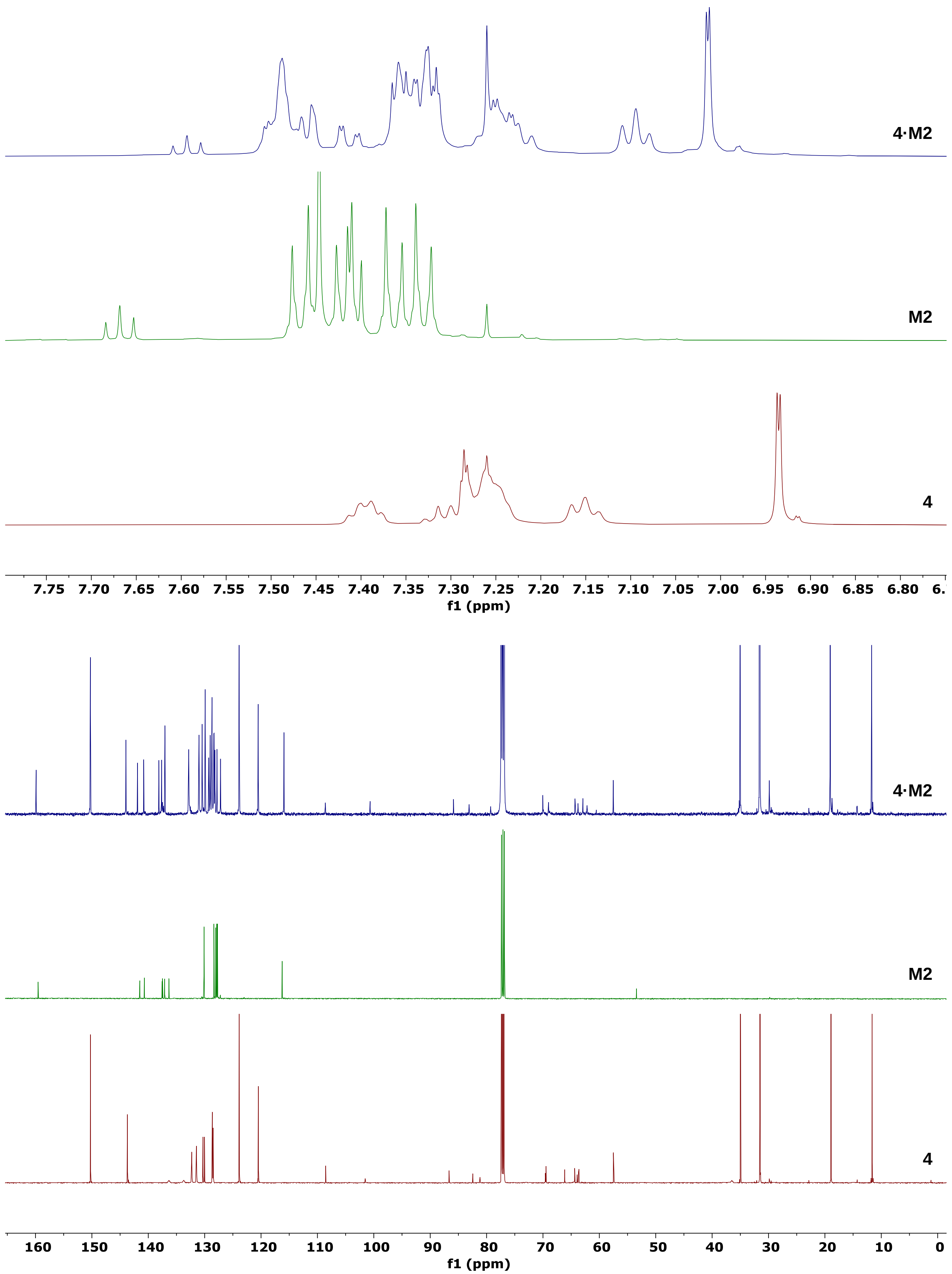

Figure S19: Stacked (top) ^{1}H NMR and (bottom) ^{13}C NMR spectra of (red) thread **4** (500/126 MHz), (green) nanohoop **M2** (600/151 MHz) and (blue) [2]rotaxane **4·M2** (500/126 MHz) ($CDCl_3$, 298 K).

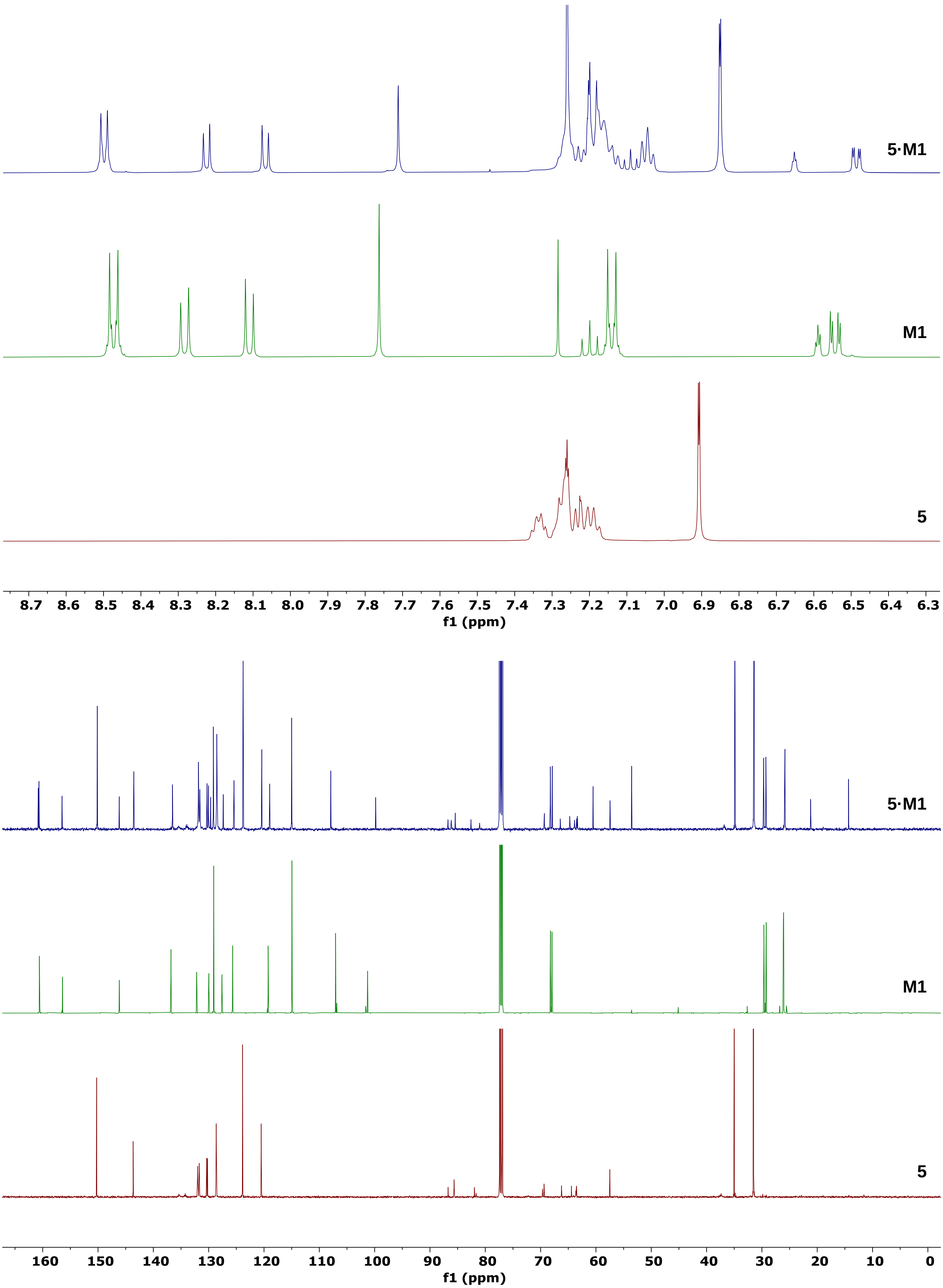

Figure S20: Stacked (top) ^{1}H NMR and (bottom) ^{13}C NMR spectra of (red) thread **5** (500/126 MHz), (green) macrocycle **M1** (600/151 MHz) and (blue) [2]rotaxane **5**·**M1** (500/126 MHz) ($CDCl_3$, 298 K).

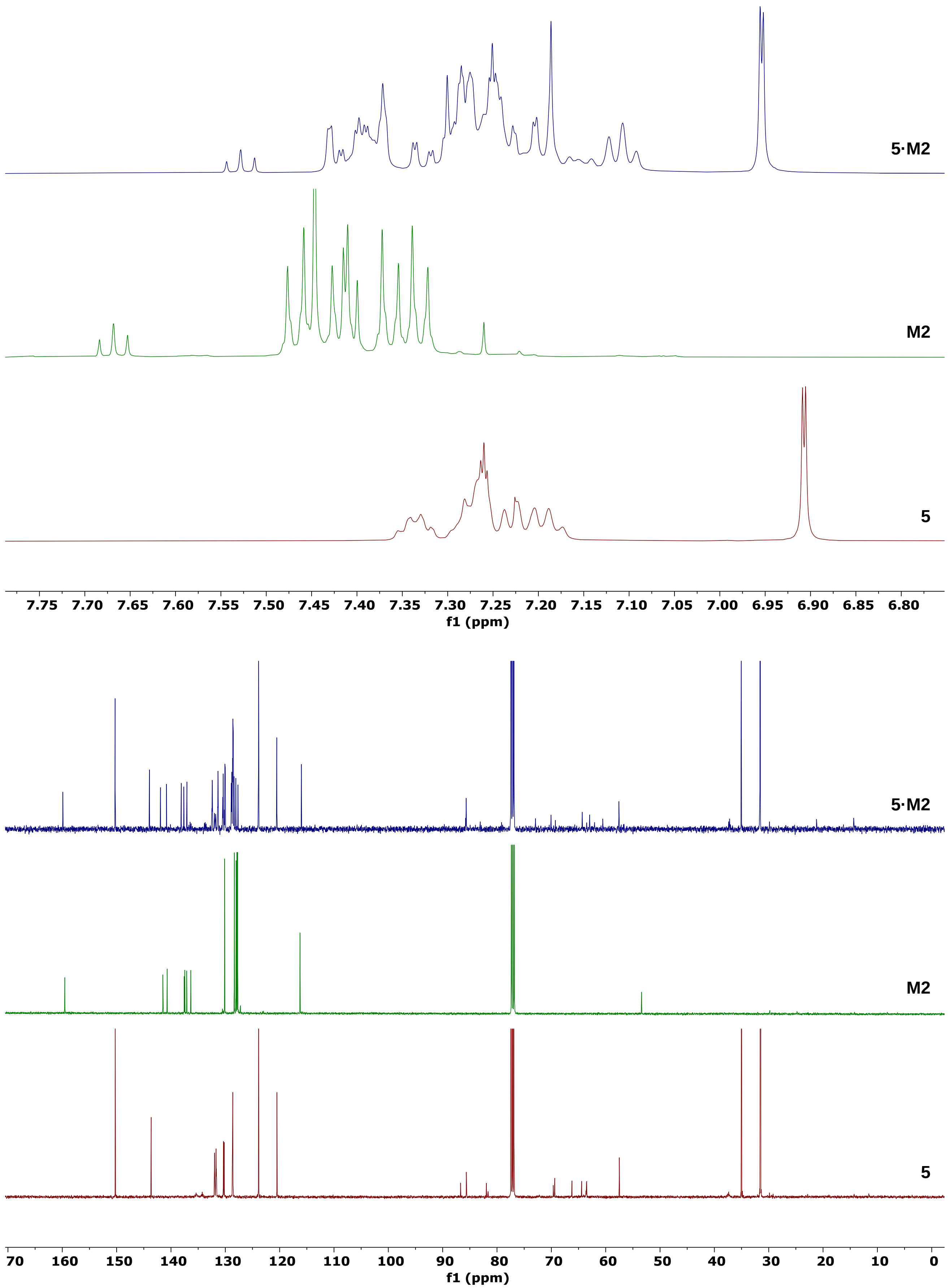

Figure S21: Stacked (top) ^{1}H NMR and (bottom) ^{13}C NMR spectra of (red) thread **5** (500/126 MHz), (green) nanohoop **M2** (600/151 MHz) and (blue) [2]rotaxane **5**·**M2** (500/126 MHz) ($CDCl_3$, 298 K).

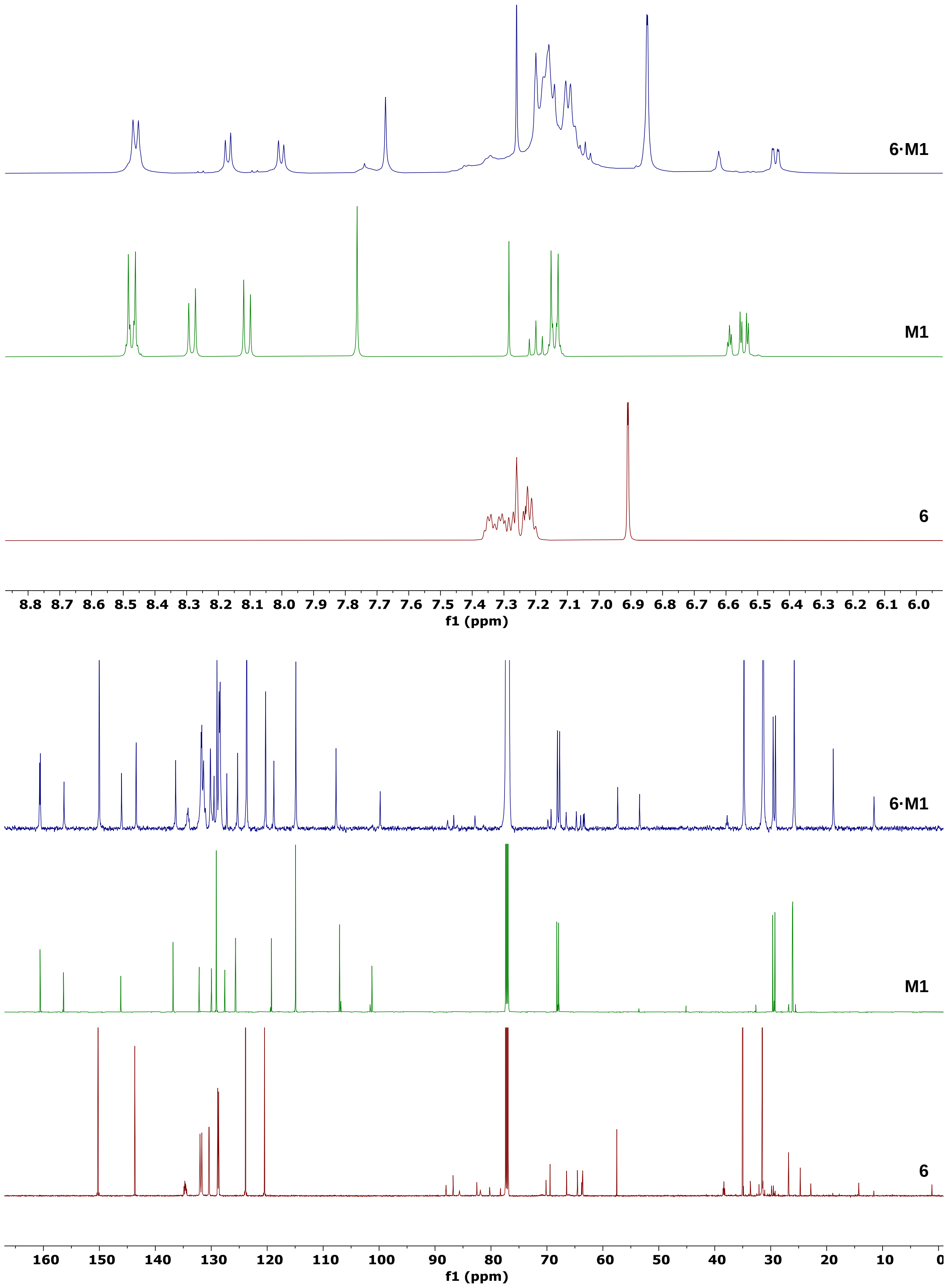

Figure S22: Stacked (top) ^{1}H NMR and (bottom) ^{13}C NMR spectra of (red) thread **6** (600/151 MHz), (green) macrocycle **M1** (600/151 MHz) and (blue) [3]rotaxane **6**·(**M1**)$_2$ (500/126 MHz) ($CDCl_3$, 298 K).

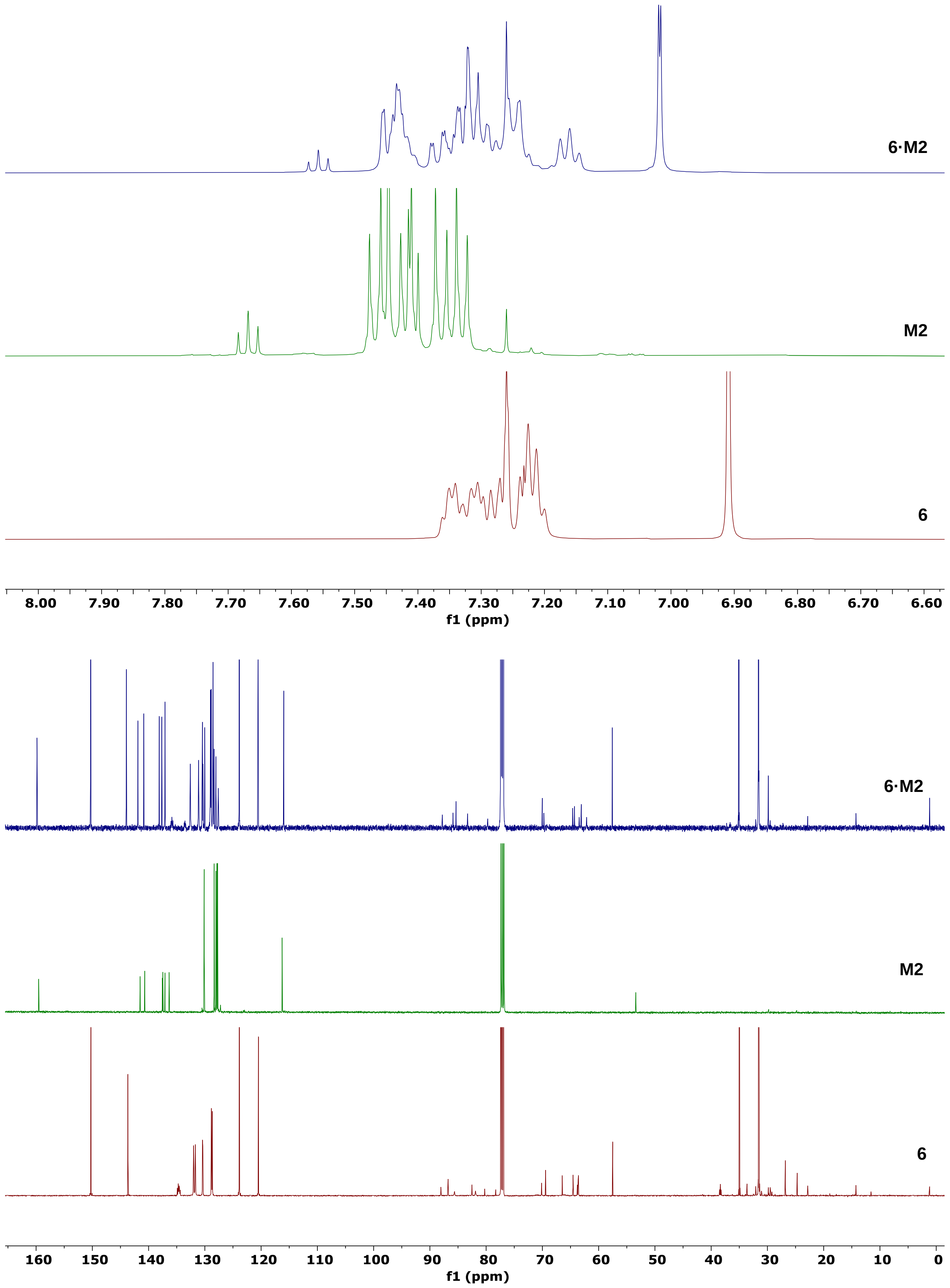

Figure S23: Stacked (top) ^{1}H NMR (600 MHz) and (bottom) ^{13}C NMR (151 MHz) spectra of (red) thread **6**, (green) nanohoop **M2** and (blue) [3]rotaxane **6**·(**M2**)$_2$ ($CDCl_3$, 298 K).

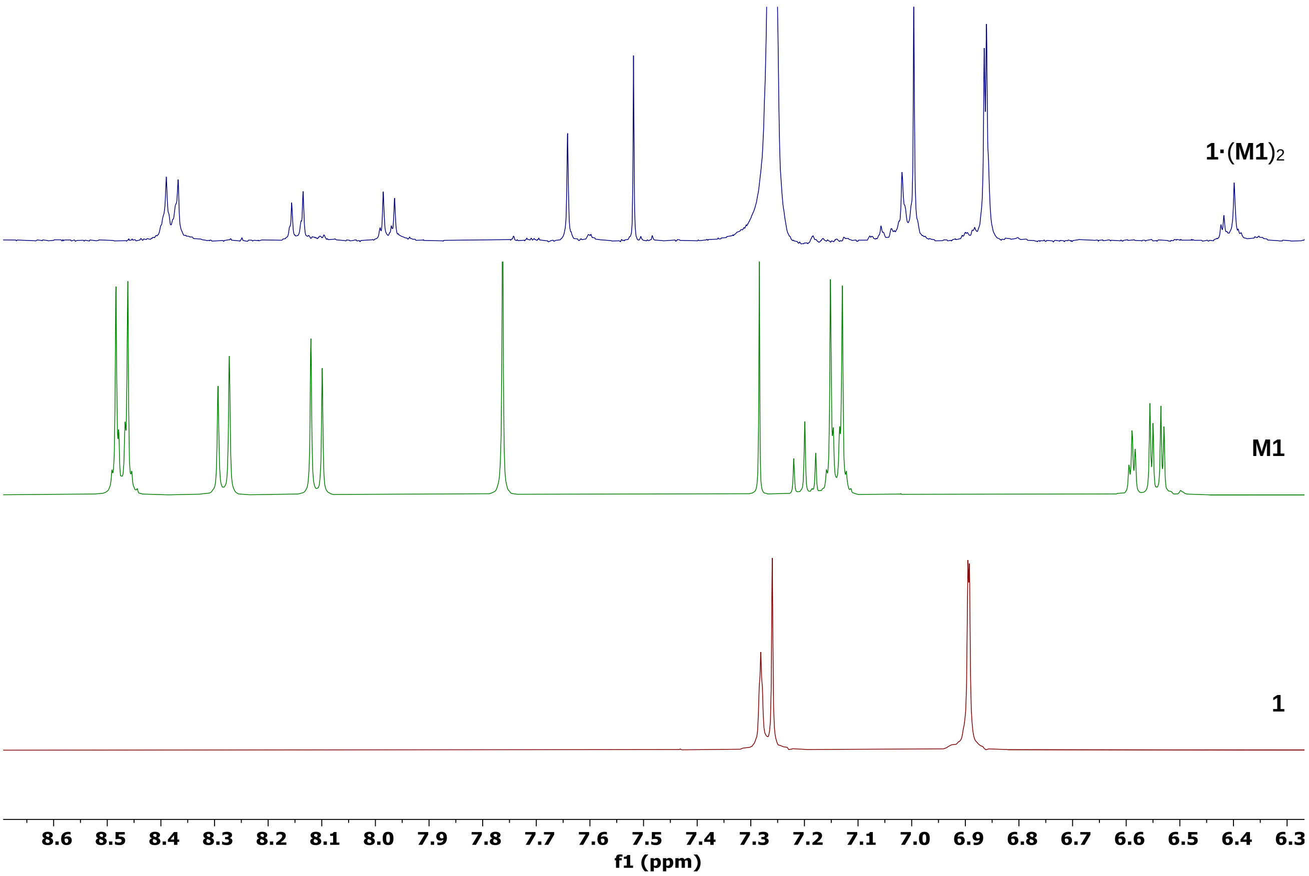

Figure S24: Stacked ^{1}H NMR spectra of (red) thread **1** (600 MHz), (green) macrocycle **M1** (600 MHz) and (blue) [2]rotaxane **1**·(**M1**)$_2$ (500 MHz) ($CDCl_3$, 298 K).

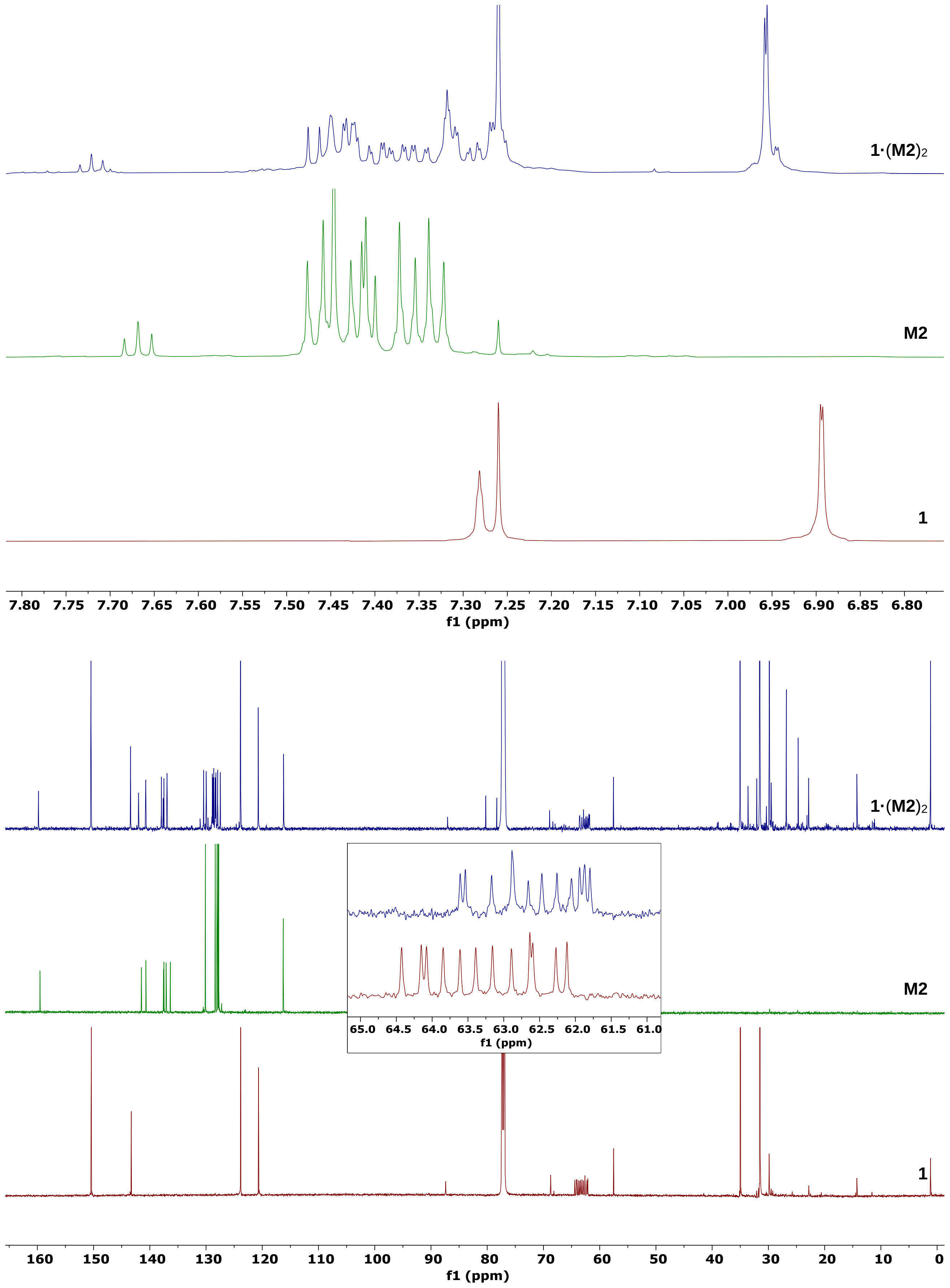

Figure S25: Stacked (top) ^{1}H NMR (600 MHz) and (bottom) ^{13}C NMR (151 MHz) spectra of (red) thread **1**, (green) nanohoop **M2** and (blue) [3]rotaxane **1**·(**M2**)$_2$ ($CDCl_3$, 298 K).

Absorption and Fluorescence Spectra

UV-vis Absorption Spectra

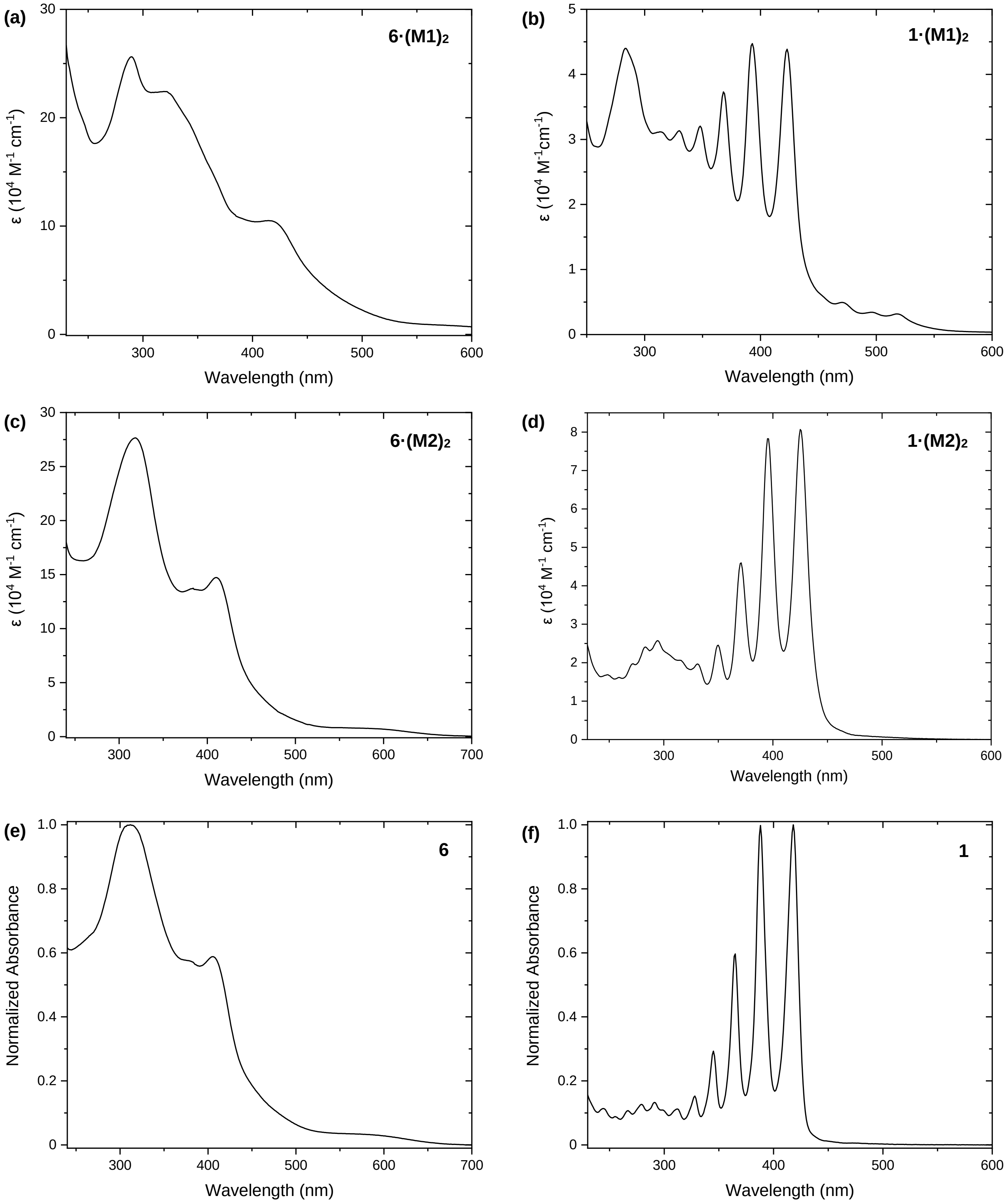

Figure S26: UV-vis spectra of (a) [3]rotaxane **6**·(**M1**)$_2$ ($CHCl_3$), (b) [3]rotaxane **1**·(**M1**)$_2$ (*n*-hexane), (c) [3]rotaxane **4**·(**M2**)$_2$ ($CHCl_3$), (d) [3]rotaxane **1**·(**M2**)$_2$ (*n*-hexane), (e) dumbbell **6** ($CHCl_3$) and (f) dumbbell **5** (*n*-hexane).

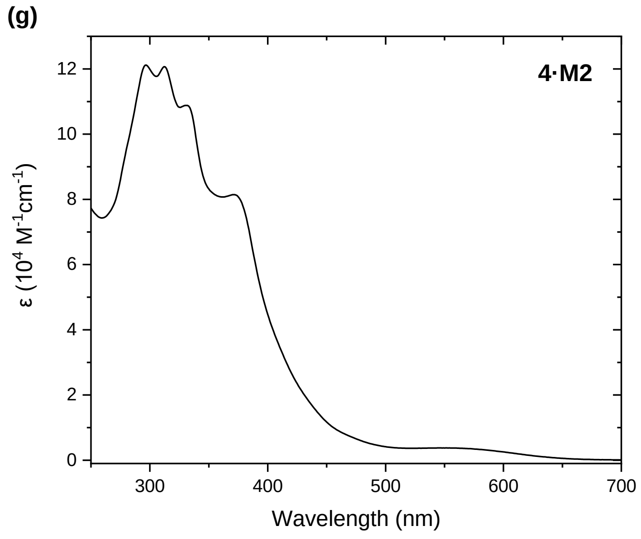

Figure S26 (*continued*)**:** UV-Vis spectrum of (g) [2]rotaxane **4•M2** ($CHCl_3$).

Fluorescence Spectra

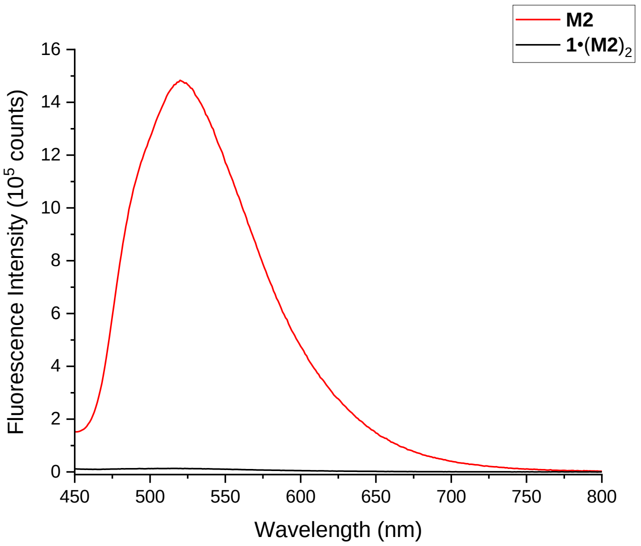

Figure S27: Emission spectra of (red) nanohoop **M2** and (black) nanohoop-shielded polyyne [3]rotaxane **1•(M2)**$_2$ (DCM, 298 K, λ_{ex} 320 nm, λ_{em} 520 nm). Sample concentrations of 4.31×10^{-7} M and 8.61×10^{-7} M for nanohoop **M2** and rotaxane **1•(M2)**$_2$, respectively.

Mass Spectra

(a)

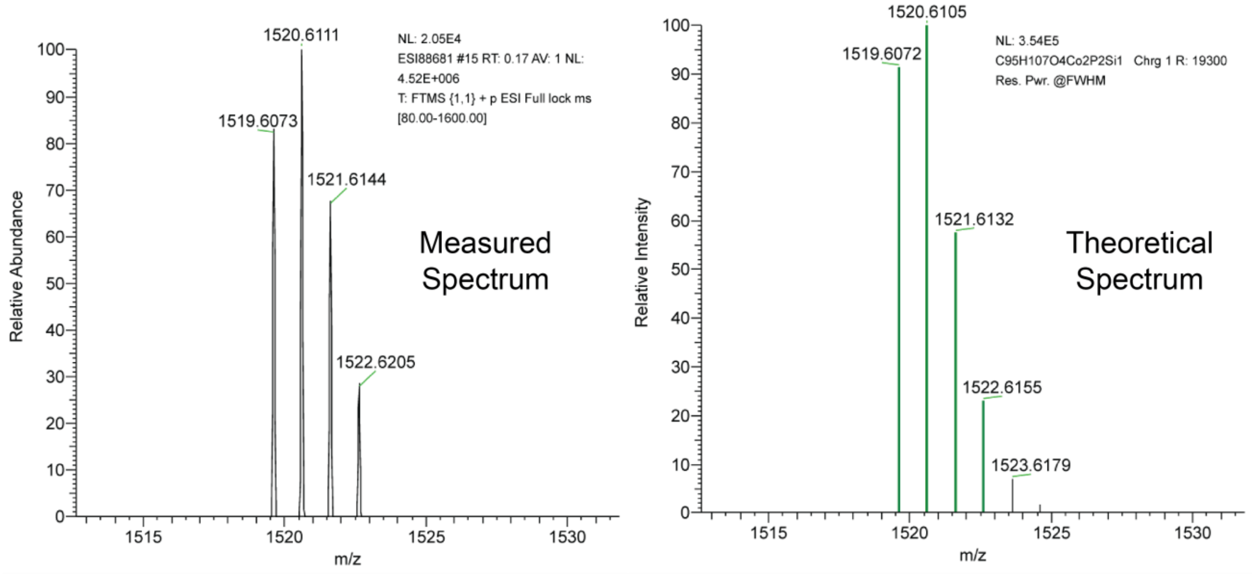

(b)

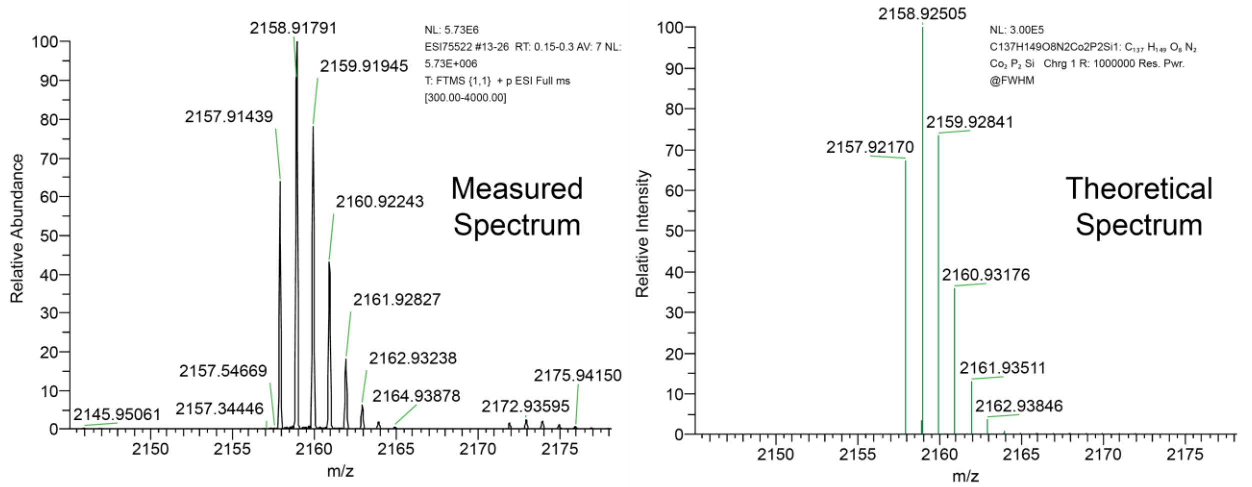

(c)

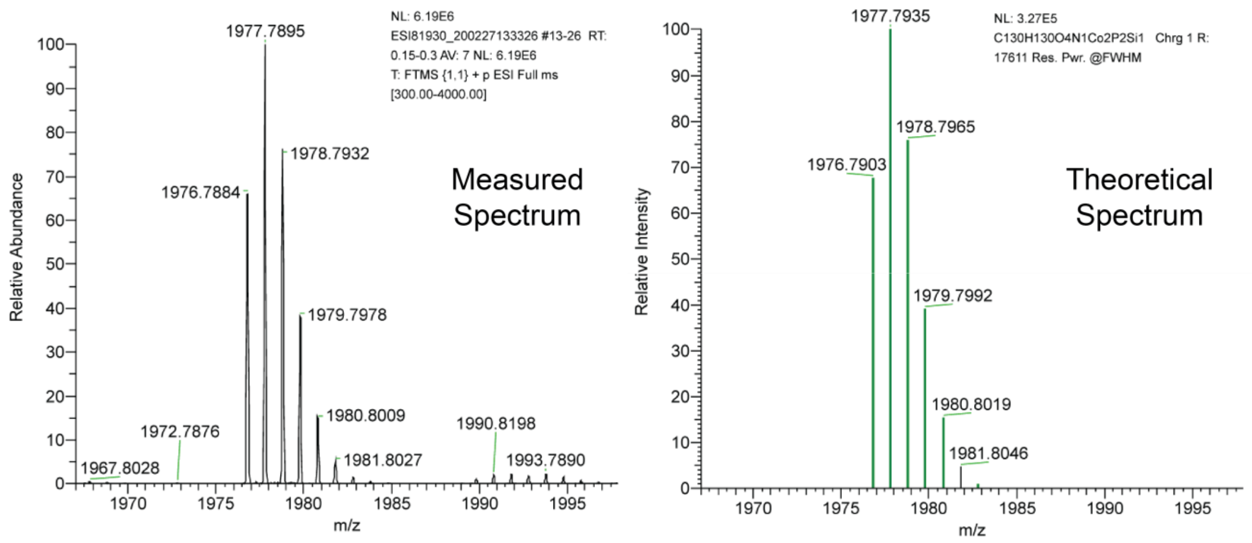

Figure S28: High-resolution mass spectra of (a) dumbbell **4**, (b) [2]rotaxane **4·M1** and (c) [2]rotaxane **4·M2**.

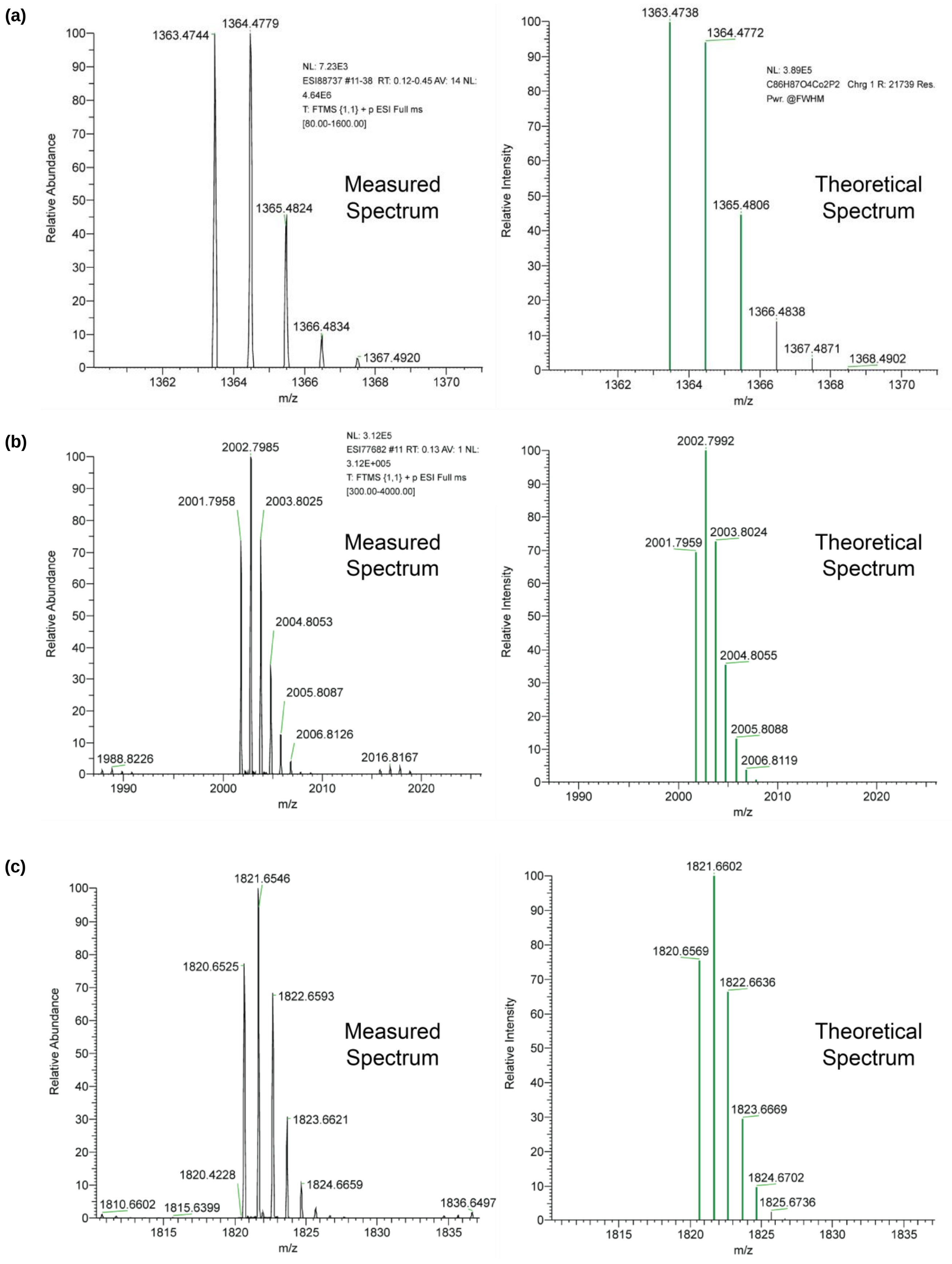

Figure S29: High-resolution mass spectra of (a) dumbbell **5**, (b) [2]rotaxane **5**·**M1** and (c) [2]rotaxane **5**·**M2**.

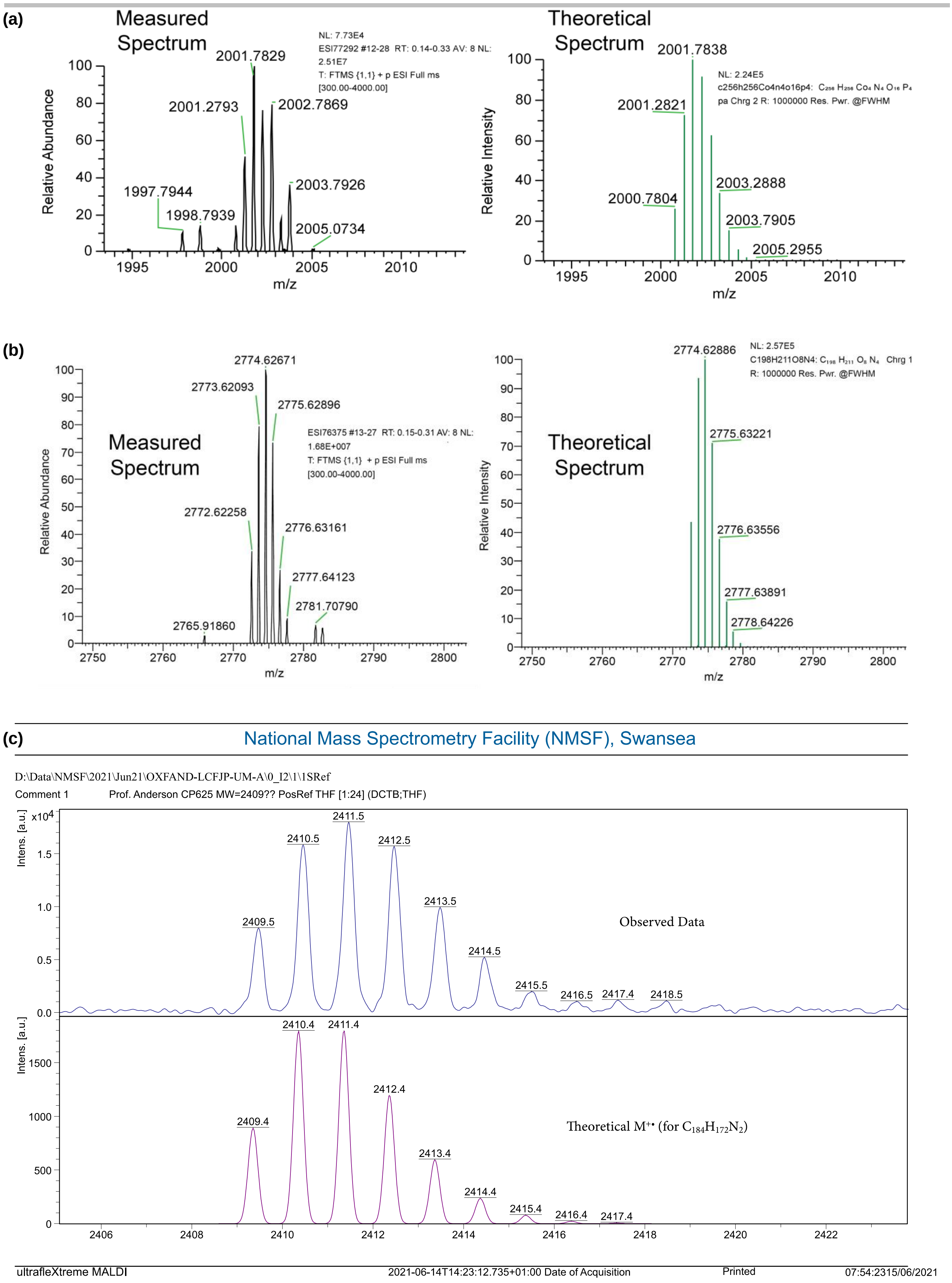

Figure S30: High-resolution (ESI) mass spectra of (a) [3]rotaxane **6**·(**M1**)$_2$, (b) polyyne [3]rotaxane **1**·(**M1**)$_2$ and (c) MALDI-TOF mass spectrum of polyyne [3]rotaxane **1**·(**M2**)$_2$ (DCTB matrix in THF used for MALDI-TOF).

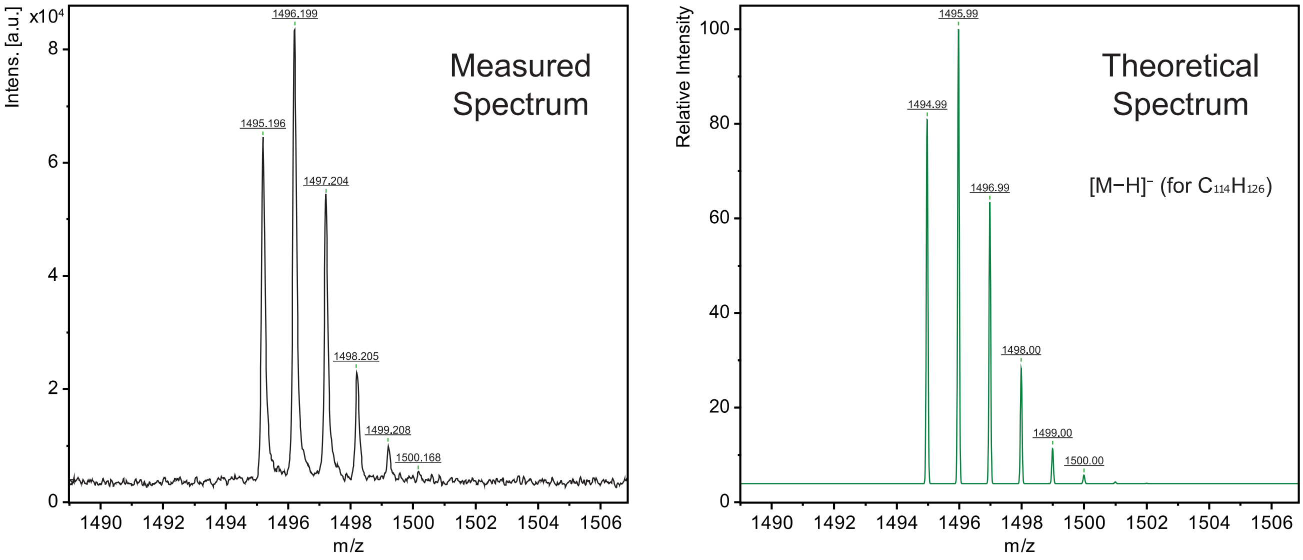

Figure S31: MALDI-TOF (DCTB matrix in THF) mass spectrum of polyyne thread **1**.

Decomposition Studies

Solution-state decomposition experiments were performed using two methods, both of which were found to give comparable kinetics.

Method 1: A 100 mL three-neck flask equipped with a PTFE stirrer bar, an argon inlet, a thermometer and a septum was placed in a large oil bath (to minimize thermal fluctuations during the experiment). Dry, degassed decalin (49.5 mL) was added and allowed to reach thermal equilibrium at 80 °C over a period of 2 h. A stock solution of sample (0.50 mL) was added to the flask, to give an initial absorption of 0.7 (path length 10 mm). Aliquots (1.5 mL) were taken at timed intervals from the initial addition (the t = 0 was taken immediately after addition) and immediately cooled to 25 °C using a water bath to prevent any further reaction. UV-vis spectra were then recorded for each aliquot. Baseline drift of the spectrometer was corrected based on an average of data points from 600-700 nm. The absorption maxima of the highest intensity signal (418 and 425 nm for the thread **1** and nanohoop [3]rotaxane **1**·(**M2**)$_2$, respectively) was plotted against time, then an exponential decay fitted to extract the rate constant of decay.

Method 2: Dry decalin (4 mL) and sufficient stock solution of sample was combined in a dry Schlenk tube, to give an initial absorption of 0.7 (path length 10 mm). The mixture was thoroughly degassed over four freeze-pump-thaw cycles, then charged with argon. Part of this solution (2.0 mL) was transferred to a dry Schlenk cuvette equipped with a PTFE stirrer bar under an argon atmosphere. The cuvette was placed into the UV-vis spectrometer, pre-heated to 80 °C using a Peltier. Once the temperature of the cuvette has equilibrated, measurements were acquired at 2 minute intervals. The data were processed using the procedure described previously.

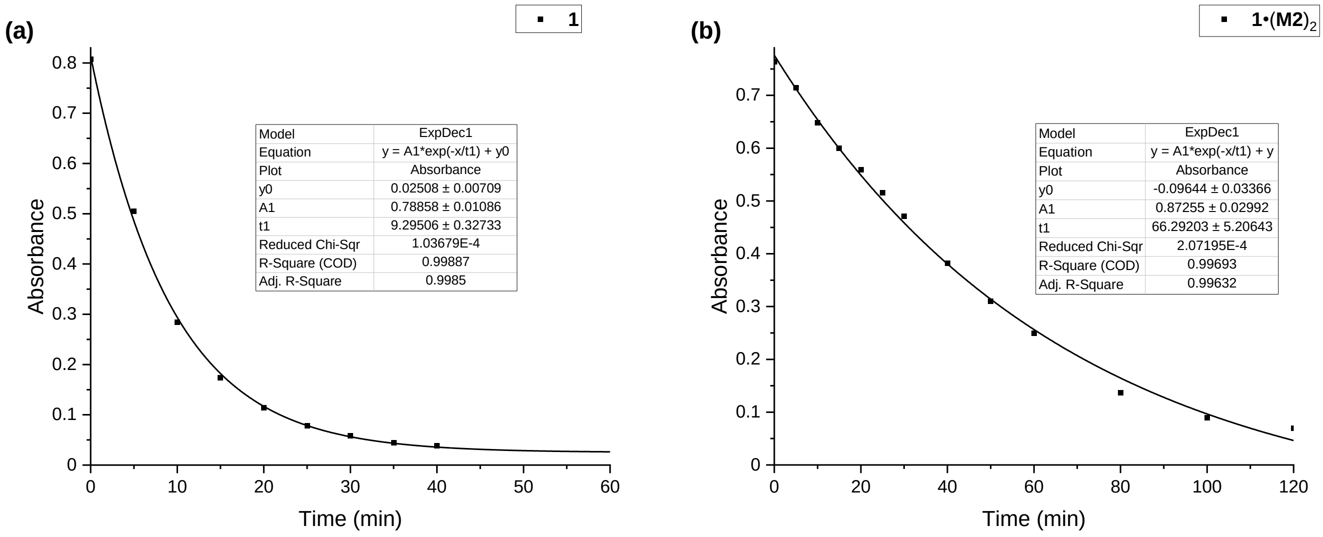

Figure S32: UV-vis decomposition studies following Method 1 (above) of (a) dumbbell **1** and (b) nanohoop polyyne [3]rotaxane **1**·(**M2**)$_2$. The absorbance of the lowest energy band was followed in each case. Data are fitted to a first order exponential decay, $A = (A_0 - A_f)\exp(-kt) + A_f$, where A, A_0 and A_f are the absorbance at time t, absorbance at t = 0 and absorbance at $t = \infty$, respectively, and k is the rate constant.

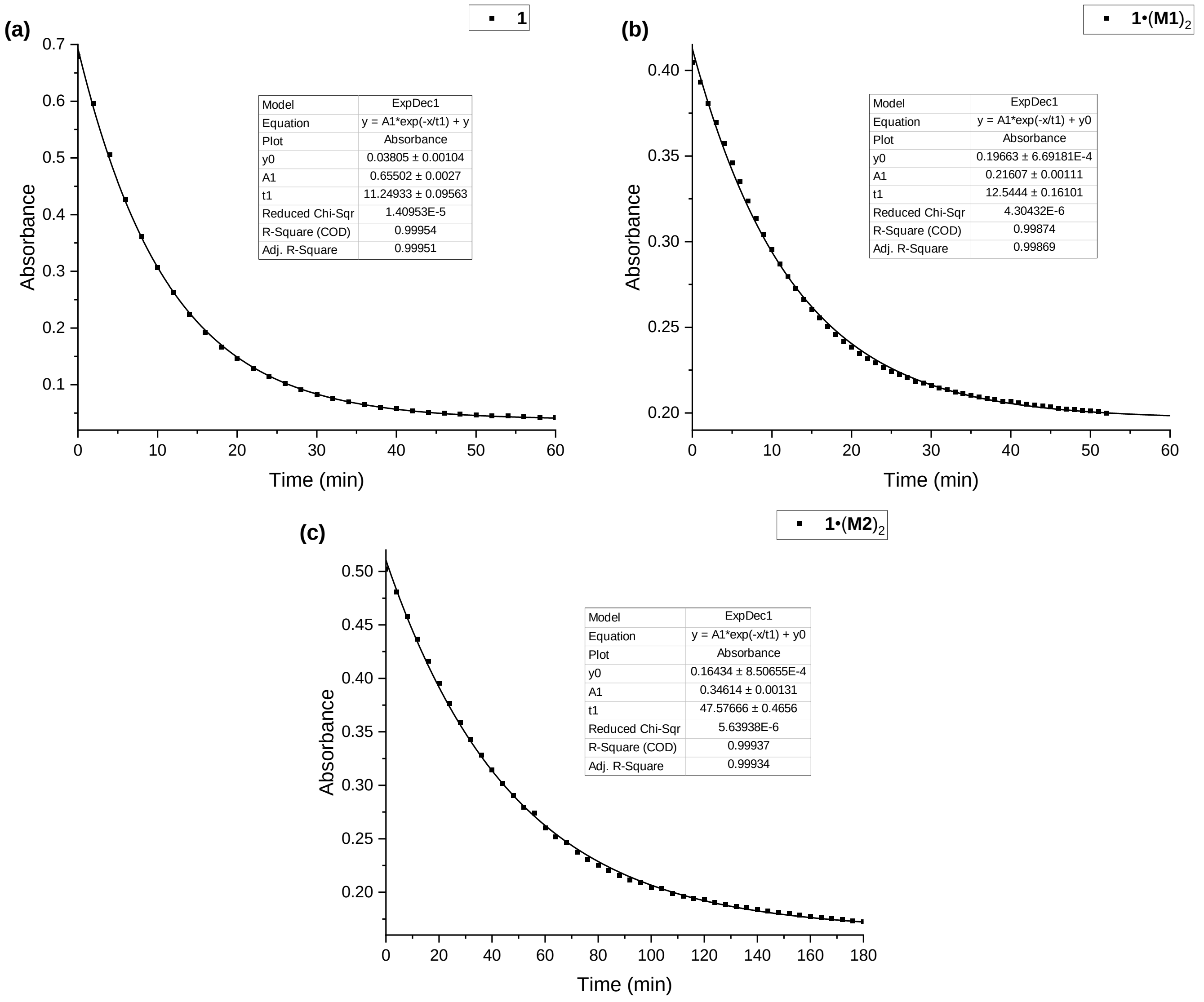

Figure S33: UV-vis decomposition studies following Method 2 (above) of (a) dumbbell **1**, (b) phenanthroline polyyne [3]rotaxane **1**·(**M1**)$_2$, (c) nanohoop polyyne [3]rotaxane **1**·(**M2**)$_2$. The absorbance of the lowest energy band was followed in each case. Data are fitted to a first order exponential decay, $A = (A_0 - A_f)\exp(-kt) + A_f$, where A, A_0 and A_f are the absorbance at time t, absorbance at $t = 0$ and absorbance at $t = \infty$, respectively, and k is the rate constant.

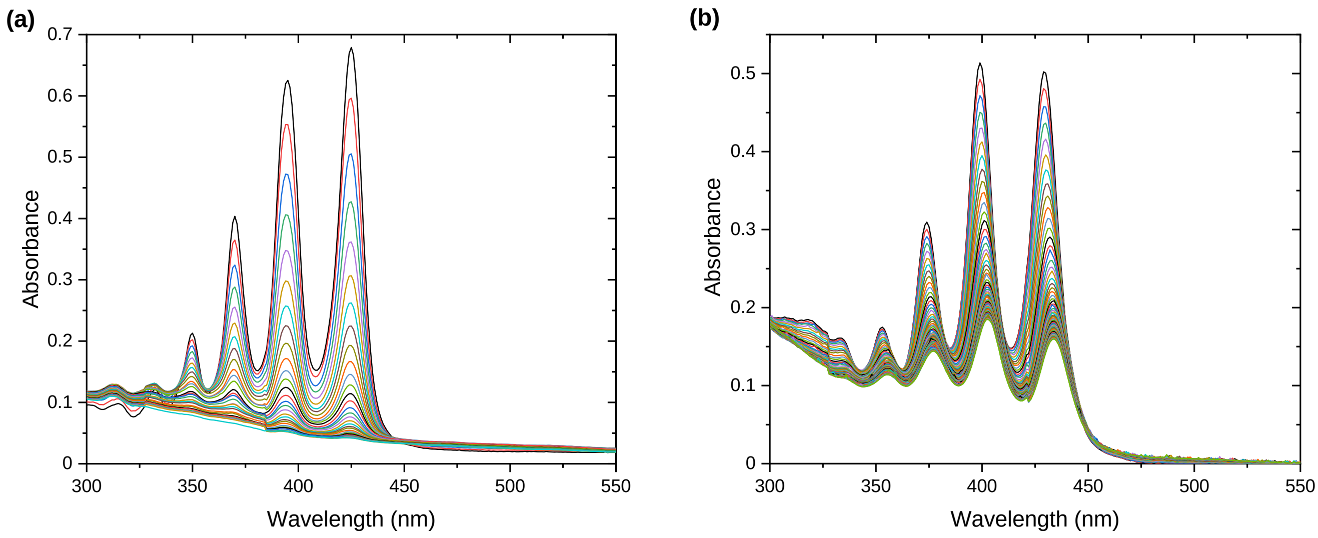

Figure S34: UV-vis spectra during the decomposition reaction following Method 2 (above) of (a) dumbbell **1** over a total period of 60 minutes, and (b) nanohoop polyyne [3]rotaxane **1**·(**M2**)$_2$ over a total period of 240 minutes. For (a) each line is separated by a 2-minute time interval, while for (b) spectra are shown for 4-minute intervals.

Differential Scanning Calorimetry

Filename: E:\DSC\Thread\CP727_250C.d6d
Operator ID:
Sample ID: CP727
Sample Weight: 2.200 mg
Comment: 100 °C to 250 °C, 10 °C/min

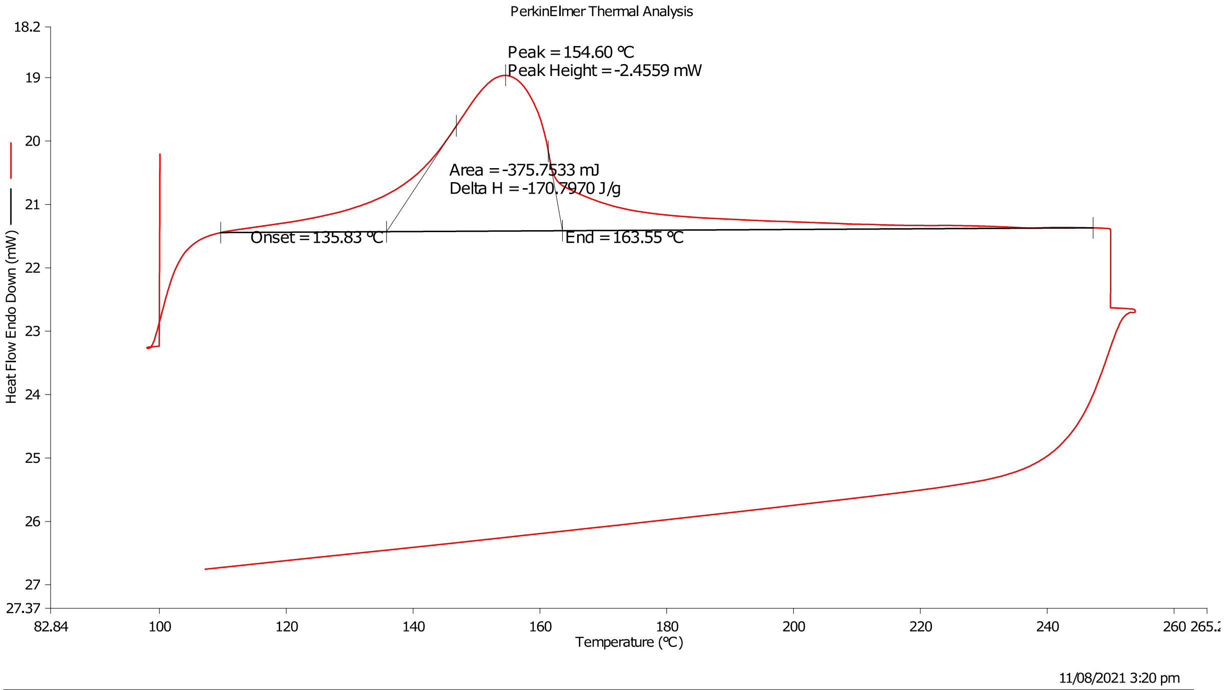

1) Hold for 1.0 min at 100.00°C
2) Heat from 100.00°C to 250.00°C at 10.00°C/min
3) Hold for 1.0 min at 250.00°C
4) Cool from 250.00°C to 100.00°C at 20.00°C/min

Figure S35: DSC trace of **1** showing a peak exotherm at 155 °C.

Filename: e:\dsc\[3]rot\cp[3]rot.d6d
Operator ID:
Sample ID: CP Nanohoop polyyne [3]rotaxane
Sample Weight: 1.700 mg
Comment: 100 °C to 200 °C, 10 °C/min

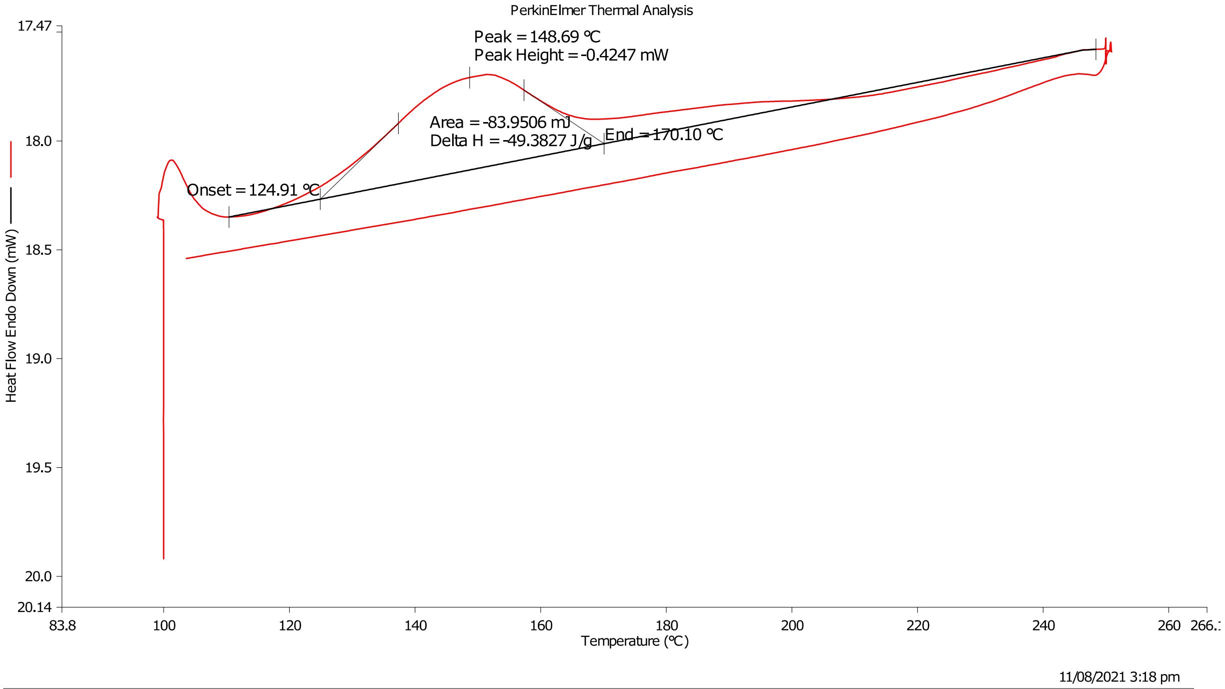

1) Hold for 1.0 min at 100.00°C
2) Heat from 100.00°C to 250.00°C at 10.00°C/min
3) Hold for 1.0 min at 250.00°C
4) Cool from 250.00°C to 100.00°C at 10.00°C/min

Figure S36: DSC trace of **1**·(**M2**)$_2$ showing a peak exotherm at 149 °C.

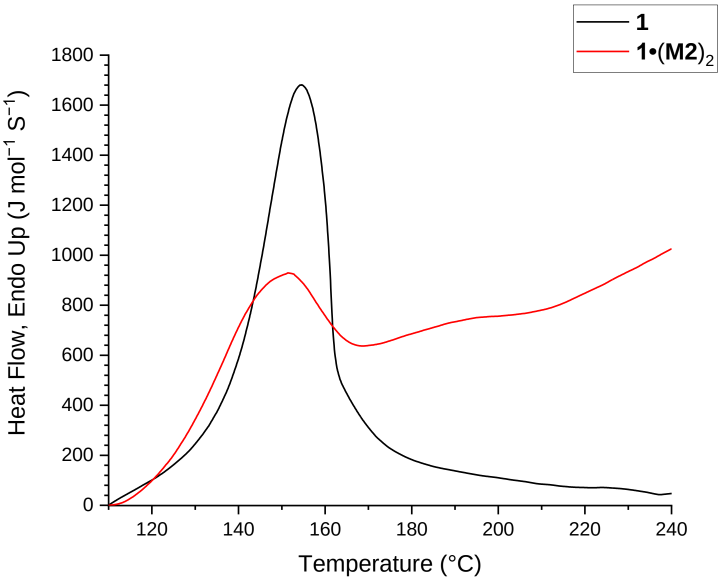

Figure S37: Overlaid traces of 14-yne thread **1** (black) and the corresponding nanohoop [3]rotaxane **1**·(**M2**)$_2$ (red). Traces have been baseline-corrected at T = 110 °C.

References

[1] R. N. Keller, H. D. Wycoff, *Inorg. Synth.* **1947**, *2*, 1–4.
[2] W. A. Chalifoux, R. R. Tykwinski, *Nat. Chem.* **2010**, *2*, 967–971.
[3] L. D. Movsisyan, M. Franz, F. Hampel, A. L. Thompson, R. R. Tykwinski, H. L. Anderson, *J. Am. Chem. Soc.* **2016**, *138*, 1366–1376.
[4] S. Eisler, N. Chahal, R. McDonald, R. R. Tykwinski, *Chem. Eur. J.* **2003**, *9*, 2542–2550.
[5] D. R. Kohn, P. Gawel, Y. Xiong, K. E. Christensen, H. L. Anderson, *J. Org. Chem.* **2018**, *83*, 2077–2086.
[6] S. Saito, K. Nakazono, E. Takahashi, *J. Org. Chem.* **2006**, *71*, 7477–7480.
[7] J. M. Van Raden, B. M. White, L. N. Zakharov, R. Jasti, *Angew. Chem. Int. Ed.* **2019**, *58*, 7341–7345.

Author Contributions

Initial synthetic work, including the preparation of compounds **2**, **3**, **4·M1**, **5·M1**, **6·M1** and **1·(M1)$_2$**, and crystallization of **4·M1**, was carried out by J.F.W., with guidance from P.G. Subsequently, C.W.P. repeated and optimized all these steps, prepared the **M2** rotaxanes and investigated the thermal stability of these compounds. C.E.O. and R.J. synthesized the nanohoop **M2**. A.L.T determined the crystal structure of **4·M1**. T.D.W.C. provided help and advice with NMR spectroscopy. C.W.P. and H.L.A. wrote the manuscript with input from the other authors.
